# Assessing Modes of Toxic Action of Organic Cations in *In Vitro* Cell-Based Bioassays: the Critical Role of Partitioning to Cells and Medium Components

**DOI:** 10.1021/acs.chemrestox.4c00527

**Published:** 2025-03-04

**Authors:** Eunhye Bae, Stephan Beil, Maria König, Stefan Stolte, Beate I. Escher, Marta Markiewicz

**Affiliations:** † Institute of Water Chemistry, Dresden University of Technology, D-01062 Dresden, Germany; ‡ Department of Cell Toxicology, Helmholtz Centre for Environmental Research-UFZ, D-04318 Leipzig, Germany; § Environmental Toxicology, Department of Geosciences, Eberhard Karls University Tübingen, D-72076 Tübingen, Germany

## Abstract

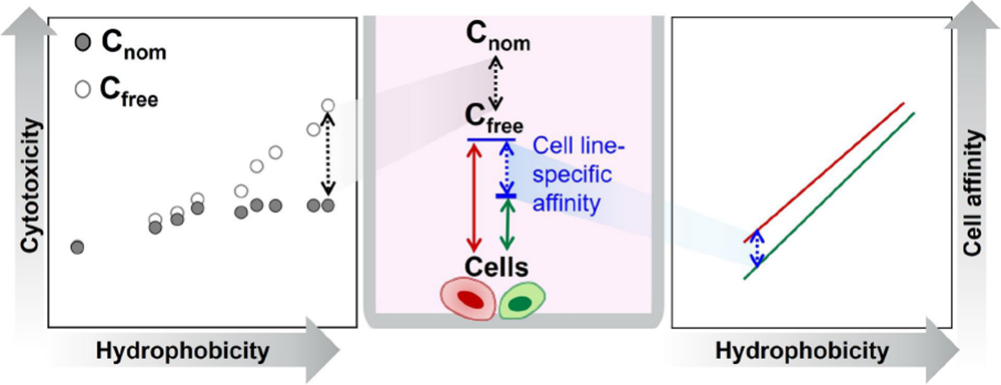

High-throughput cell-based bioassays can fulfill the growing need to assess the hazards and modes of toxic action (MOA) of ionic liquids (ILs). Although nominal concentrations (*C*_nom_) are typically used in an *in vitro* bioassay, freely dissolved concentrations (*C*_free_) are considered a more accurate dose metric because they account for chemical partitioning processes and are informative about MOA. We determined the *C*_free_ of IL cations in AREc32 and AhR-CALUX assays using both mass balance model (MBM) prediction and experimental quantification. Partition coefficients between membrane lipid–water (*K*_mw_), serum albumin–water (*K*_albumin/w_), and cell-water (*K*_cell/w_) as well as potential confounding factors (binding to a test plate and micelle formation) were determined to improve the MBM prediction. IL cations showed a higher affinity for both cell lines than that predicted by the MBM based on *K*_mw_ and *K*_albumin/w_. Their affinity for the AhR-CALUX cells was more than 1 order of magnitude higher than for the AREc32, signifying cell line-specific affinity. The MBM with an experimental *K*_cell/w_ accurately predicted *C*_free_. Evaluating cytotoxicity based on *C*_free_ eliminated the leveling off of toxicity observed for hydrophobic IL cations (side chain cutoff), suggesting that *C*_nom_ underestimates the effects of compounds with high affinity for the assay medium. Cell membrane concentrations calculated from *C*_free_ using *K*_mw_ were compared to the critical membrane burden to identify whether IL cations act as baseline toxicants. The IL cations carrying 16 carbons in the chain in the AREc32 assay and most of the IL cations in the AhR-CALUX assay were classified as excess toxicants. However, since the reasons for the deviation of experimental *K*_cell/w_ from MBM prediction remain unexplained, it is uncertain whether the cell membrane concentrations can be well predicted from *K*_mw_ used in this study. Therefore, future studies should aim to uncover the underlying causes of differing cell affinities observed across cell lines and model predictions.

## 1 Introduction

Ionic liquids (ILs) were initially recognized as versatile (and green) solvents but currently have a much broader field of applications, thanks to their advantageous physicochemical properties. With an increasing number of scientific publications and patent applications over the last two decades,^1^ ILs are currently being used in over 50 commercial or pilot-scale processes.^2^ Applications of ILs include catalysts for alkylation reactions (Chevron Corp.,^3^ or Well Resources Inc.^4^), dissolution for cellulose (Natural fiber welding)^5^ or plastic regeneration (Ioniqa),^6^ electrolytes for ion batteries (Solvionic),^7^ supercapacitors (ELYTE Innovations)^8^ or dye-sensitized solar cells (IoLiTec),^9^ and active pharmaceutical ingredients (Teikoku/IBSA Institut Biochimique).^10^ As market forecasts put the annual growth of ILs-dependent industries at 10% in the upcoming years,^11^ the production of ILs will undoubtedly grow further. Increased use of chemicals is often accompanied by their increased release into the environment and exposure of biota and humans. Indeed, recent work has reported the detection of relatively high concentrations of chemical entities likely to have originated from ILs in the environment^12−14^ and even in human blood.^15^ This raises concerns about the potential hazards of ILs to the environment and human health, which necessitate immediate attention.

For many years, efforts have been made to understand the hazards of ILs and relate them to their chemical structure in order to facilitate the design of environmentally benign ILs at the early commercial stage.^16−18^ However, most toxicity studies have focused on the assessment of apical endpoints, which allows for comparative proactive hazard assessment but provides only a limited understanding of the underlying mechanisms of toxicity. Meanwhile, with the development of new approach methodologies (NAMs) for rapid, non-animal hazard screening, the use of *in vitro* assays not only allows for higher throughput of testing but also provides better ways to link molecular targets of chemicals to adverse toxicological outcomes and to identify the modes of toxic action (MOA).^19^ However, the implementation of *in vitro* tests for toxicological risk assessment faces challenges in translating *in vitro* potencies to equivalent *in vivo* effects.^20^ Thus, recent studies have attempted to establish quantitative *in vitro-in vivo* extrapolation (QIVIVE) to link the *in vitro* concentrations and *in vivo* doses by incorporating pharmacokinetic and concentration-dependent bioactivity information.^21,22^ The nominal concentration (*C*_nom_) is predominantly used for QIVIVE because of its availability, while it does not account for various partitioning and loss processes that may alter the actual exposure concentrations available for uptake and exerting effects in the cellular assay.^23,24^ In such a case, the freely dissolved concentration in the medium (*C*_free_) is considered a better metric because the *C*_free_ can account for all possible “losses” in the bioassay and can be directly compared to the plasma concentration in the *in vivo* assay.

The relationship between *C*_nom_ and *C*_free_ depends on the chemical distribution and loss processes in an *in vitro* system, including evaporation,^25,26^ binding to test vessels,^27,28^ cell metabolism,^29^ partitioning to medium constituents,^30^ and cells.^27,31^ If these processes can substantially reduce the freely dissolved fraction of a chemical, reporting *in vitro* effect data based on *C*_nom_ may underestimate the true toxic potency of the chemical. However, *C*_free_-based exposure assessment in *in vitro* bioassays is severely limited due to analytical difficulties, particularly for tests conducted in a high-throughput system (HTS). Alternatively, a mass balance model (MBM) can be used to predict chemical distribution in *in vitro* bioassays by using partition coefficients between different phases present in the system (cells, medium, plate material) and the volumes of the respective system components (e.g., protein, lipid, water) in a set of equations.^32,33^ Recent efforts to determine chemical-specific partition coefficients and to quantify *C*_free_ in a bioassay medium have extended the applicability of the MBM from neutral organic chemicals^32^ to ionizable chemicals.^34,35^ However, few studies have applied the MBM to the permanently charged organic cations like IL. The limited availability of model inputs (e.g. experimental partition coefficients between membrane lipid and water or serum albumin and water) or the difficulties in measuring *C*_free_ may hinder the model development and its validation for IL cations. Their unique nature, derived from the positive charge or structural similarity to surfactants, may alter the partitioning behaviors in *in vitro* bioassays, for instance by aggregate formation^36^ or by developing the electrostatic interaction with negatively charged substrates,^37^ which should also be considered in the MBM. Therefore, filling the data gap and characterizing all potential partitioning processes are essential prerequisites for adapting the MBM to IL cations.

In addition, understanding the chemical distribution in bioassays enables the identification of MOA. For example, baseline toxicity is a non-specific effect caused by disturbance of membrane integrity and function due to chemical partitioning into biological cell membranes.^38^ Principally, the concentrations in cellular membranes of chemicals that cause baseline toxicity, which is referred to as the critical membrane burden (CMB), are constant irrespective of the test chemicals or test species.^39^ Since the membrane concentrations of chemicals with a specific MOA would generally be lower than the CMB for baseline toxicants,^38^ a comparison of the membrane concentrations with the CMB can quantify the degree of specificity of a chemical. Assuming that the internal cellular *C*_free_ is equal to the medium *C*_free_, the cellular membrane concentration can be estimated from the medium *C*_free_ using the membrane lipid–water partition coefficient. With this approach, Huchthausen et al. defined the CMB causing 10% cytotoxicity for reporter gene-based bioassays as 26 mmol/L_lip._^40^ This CMB value can serve as an anchor to distinguish baseline toxicants from excess toxicants, designating the latter for further testing to identify the specific MOA.

This study aims to characterize the partitioning and loss processes of IL cations in *in vitro* cell-based bioassays to extend and verify the applicability domain of the MBM. A series of homologous IL cations with side chains of varying length (1-methyl-3-alkylimidazolium chlorides (IM1-R Cl, R: 8–16 carbons) and alkyldimethylbenzylammonium chlorides (N11-R-1Ph Cl, R: 10–16 carbons) were selected as test compounds to evaluate the influence of hydrophobicity on chemical distribution. IL cations with various headgroup structures (e.g., phosphonium, pyridinium, piperidinium) were also included for the determination of partition coefficients. Partition coefficients between membrane lipid–water (*K*_mw_), albumin-water (*K*_albumin/w_), and cell-water (*K*_cell/w_) were determined to quantify the interactions of IL cations with each compartment of the *in vitro* bioassay and to provide reliable inputs to the MBM. Additionally, the possibility of micelle formation and the impacts of plastic plate binding were evaluated as possible confounding factors influencing the chemical distribution. Since *C*_free_ in the medium is the experimentally accessible dose metric compared to, e.g., total concentrations in cells or in cell membranes,^23^*C*_free_ in the bioassay medium was quantified and used to validate the accuracy of the MBM prediction. The experimentally determined *C*_free_ was further employed to evaluate the cytotoxicity of IL cations, demonstrating the importance of the *C*_free_-based exposure assessment in *in vitro* bioassays. Finally, the results were used to estimate the cellular membrane concentrations to evaluate whether IL cations act as baseline toxicants or have a more specific MOA.

## 2 Material and Methods

### 2.1 Chemicals

All IL cations used in this study are listed together with their CAS number, full name, abbreviation, chemical purity, and suppliers in Table S1 and their respective structures and molecular weights are listed in Table S2. For LC-MS/MS analysis, LC/MS grade methanol, acetonitrile, formic acid (VWR International GmbH, Germany), water (Fischer Scientific GmbH, Germany), ammonium formate (VWR International GmbH, Germany), and NH_4_OH (VWR International GmbH, Germany) were used. Nile red was purchased from MP Biomedicals GmbH, Germany. Phosphate buffered saline (PBS, 137 mM NaCl, 12 mM phosphate, pH 7.4) and dimethyl sulfoxide (DMSO) were purchased from Thermo Fisher Scientific (Schwerte, Germany). For the *in vitro* cell-based assays, Dulbecco’s modified Eagle’s medium (DMEM GlutaMax), fetal bovine serum (FBS), and 100 U/mL penicillin-streptomycin were purchased from Thermo Fischer Scientific (Schwerte, Germany). The AREc32 cells and AhR-CALUX cells were kindly provided by C. Roland Wolf, Cancer Research UK, and Michael Denison, UC Davis, USA, respectively.

### 2.2 Analytical Method

The concentrations of tested IL cations were quantified using a liquid chromatograph ExionLC coupled to a SCIEX triple quadrupole mass spectrometer (SCIEX TripleQuad 4500) operated in ESI(+) mode with a source temperature of 450 °C and an ion spray voltage set to 5500 V. The separation of the compounds was performed at a flow rate of 0.4 mL/min on a TSKgel Amide-80 column (150 × 2 mm, 3 μm particle size) at 40 °C. A solvent gradient composed of eluent A (1.25% formic acid, 5% acetonitrile, and 10 mM ammonium formate in water) and eluent B (1.25% formic acid, 5% water in acetonitrile) was used. The external calibration was prepared in a mixture of methanol and matrix (PBS or DMEM) 98:2 (v/v). The concentrations ranged from 100 ng/L to 25 μg/L. Further details on the LC and MS parameters can be found in the SI (Table S3).

### 2.3 Determination of Critical Micelle Concentration (CMC)

Due to the amphiphilic nature (i.e., a charged (polar) headgroup and an apolar chain), IL cations can aggregate into micelles at concentrations above the critical micelle concentration (CMC).^41^ As the formation of micelles could alter the distribution of compounds in the bioassay by creating another discrete phase,^36^ the CMC values need to be considered in studying the distribution of IL cations in the assay medium. As the CMC tends to decrease with increasing alkyl chain length of IL cations and salinity of the medium,^36^ homologues of 1-methyl-3-alkylimidazolium chlorides (IM1-R Cl, R:12–16) and benzalkonium chlorides (N11-R-1Ph Cl, R:10–16) were tested in PBS buffer.

The change in the fluorescence of 9-diethylamino-5-benzo[*a*]phenoxazinone (Nile red) between aqueous and hydrophobic/lipid environments was used to estimate the concentration at which the IL cation micelles spontaneously form in PBS. When the aqueous concentrations exceed the CMC, the Nile red associates with the hydrophobic domain of the micelles and shows increased fluorescence intensity.^39^ The inflection point on the fluorescence concentration curve indicates the CMC. A 1 g/L stock solution of Nile red was prepared in acetone in a nontransparent glass vial and stored in the dark at 4 °C for up to three months. The working solution (10 mg/L) was freshly prepared in PBS buffer for each new test. The solutions of the test chemicals prepared in PBS buffer were serially diluted in a 96-well black plate, spanning two to 3 orders of magnitude in concentration (100 μL/well). After adding 5 μL of the Nile red working solution to each well, the plate was gently vortexed and kept in the dark at room temperature for 30 min. The fluorescence of the Nile red was measured at Ex/Em = 552/636 nm using a microplate reader (BMG Labtech, Germany). The fluorescence was then plotted against the concentration of the IL cation. To quantitatively identify an inflection point, two linear regions of the fluorescence intensity versus concentration curve were identified: premicelle formation (concentration below the inflection point) and postmicelle formation (concentration above the inflection point). A linear regression was fitted to each segment, and the CMC was calculated as the concentration at which the two regression lines intersected.

### 2.4 Sorption to a Plastic Plate

Sorption to a 96-well plastic plate was investigated for a homologous series of IM1-R Cl and N11-R-1Ph Cl with chains ≥12 carbons, as the IL cations with a shorter alkyl chain showed negligible plastic sorption in a preliminary test (data not shown). The tests were conducted in both serum-free and serum-containing mediums to assess the influence of serum on sorption to plastic. A tissue culture (TC)-treated 96-well plate with a flat bottom (Corning-3596) was used for the test. The serum-containing medium used in the AREc32 and AhR-CALUX bioassays comprised DMEM GlutaMax supplemented with 10% fetal bovine serum (FBS) and 100 U/mL penicillin-streptomycin. In the serum-free medium, 10% FBS was replaced with LC/MS grade water. All tests were performed in the absence of cells to avoid any potential chemical losses due to cellular uptake.

Glass dosing vials were prepared by spiking methanolic stock solutions of the tested IL cations in the medium. The medium from the dosing vial was sampled to determine the initial medium concentration at 0 h (*C*_medium_ at *t*_0h_). The test chemicals were added to a well plate in serial dilutions in triplicate, resulting in a final volume of 150 μL in each well (a final methanol content < 0.5% in all wells). The dosing concentration spanned up to 3 orders of magnitude and included nominal concentrations at 10% cytotoxicity (IC_10,nom_) reported in a previous study.^42^ The dosed multi-well plate was then sealed and incubated at 37 °C for 24 h. After 24 h of incubation, the medium was sampled to quantify the medium concentrations of the compounds at 24 h (*C*_medium_ at *t*_24h_). Samples taken from the serum-free medium were diluted with 80% (v/v) methanol and filtered through a 0.2 μm syringe filter made of regenerated cellulose. Samples from the serum-containing medium were diluted with 80% (v/v) acetonitrile containing 0.1% formic acid in an HPLC vial to precipitate serum constituents. Following centrifugation at 10,000 rpm for 10 min, 200 μL of the supernatant was transferred to a new HPLC vial and used for quantification by LC/MS/MS. The difference between the initial medium concentration (*C*_medium_ at *t*_0h_) and the medium concentration after 24 h (*C*_medium_ at *t*_24h_) was used to calculate the amount of chemical sorbed on the plastic plate (*q*_e_, μmol/cm^2^). *q*_e_ (μmol/cm^2^) was expressed as the number of moles of chemical per surface area (*A*, cm^2^) which was calculated to be 1.21 cm^2^ for a volume of 150 μL (eq 1) to provide a sorption isotherm as a function of *C*_medium_ at *t*_24h_.
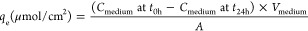
1

### 2.5 Partitioning to Bioassay System Components

#### 2.5.1 Membrane Lipid–Water Partition Coefficients (*K*_mw_)

The solid-supported lipid membranes (SSLM), commercially available from Sovicell GmbH (Leipzig, Germany) as TRANSIL, were used to determine the *K*_mw_ of 17 IL cations covering a wide range of structures. According to the product certificate of analysis, the SSLM constitutes 96% purified egg yolk phosphatidylcholine (POPC), different types of phospholipids (e.g., 1% lysophosphatidylcholine, 1% sphingomyelin, and 0.1% phosphatidylethanolamine), as well as 0.5% cholesterol, 0.2% fatty acids, and 0.5% triglycerides. In these systems, egg yolk POPC bilayers are noncovalently bound to porous silica beads. The standard TRANSIL Intestinal Absorption bead suspension was used for IL cations with predicted log *K*_mw_ values higher than 1.5, while the TRANSIL Intestinal Absorption bead suspension for low-affinity compounds was used for more hydrophilic compounds (predicted log *K*_mw_ values <1.5). Both products were obtained as suspensions. The dry weight of the SSLM suspension and its lipid content were provided in the product certificate of analysis as follows: 243 mg/mL_suspension_ (dry weight) and 12.0 ± 0.6 μL_lipid_/mL_suspension_ (lipid content) for the standard TRANSIL Intestinal Absorption beads; 225 mg/mL_suspension_ (dry weight) and 68.6 ± 0.6 μL_lipid_/mL_suspension_ (lipid content) for the TRANSIL Intestinal Absorption beads for low-affinity compounds. Experiments were performed in duplicate as previously described.^43,44^ All stock solutions were prepared in PBS buffer containing a maximum of 10% DMSO and diluted with PBS to contain <1% DMSO in the TRANSIL assay. The volume of SSLM bead suspension added was adjusted to provide approximately 20 to 70% binding based on the predicted *K*_mw_ value. The molar ratio of lipid to test compound sorbed in the lipid phase (lipid/sorbed compound) was maintained above 60 to prevent membrane saturation according to the product protocol.^45^ Membrane saturation may lead to non-linearity in partitioning and underestimation of log *K*_mw_. To examine the impact of exceeding the molar ratio cut-off (>60) on *K*_mw_, a few imidazolium ILs that had previously been tested at molar ratios <60 were additionally retested.^43,46^ Control vials without SSLM beads (containing chemicals at the same nominal concentration in PBS buffer) served as a reference to compensate for any non-specific losses. Five-point isotherms were obtained for compounds with log *K*_mw_ > 1.5. For low-binding compounds with log *K*_mw_ < 1.5, only one or two points could be measured reliably (sorbed fractions of 20–50%).^43,44^

The test solutions mixed with the appropriate volume of SSLMs were incubated in HPLC vials at 37 °C for 30 min on a shaker at 600 rpm. After centrifugation at 10,000 rpm for 10 min, the supernatant was diluted with methanol (the final methanol content ≥ 80% (v/v)) in glass vials and analyzed by LC/MS/MS (*C*_supernatant,TRANSIL_). For compounds tested in the standard TRANSIL Intestinal Absorption beads suspension for five-point isotherms, the log *K*_mw_ values were determined from the slopes of each sorption isotherm. For low binders (log *K*_mw_ < 1.5), the log *K*_mw_ at each concentration point was calculated according to eq 2 and averaged:
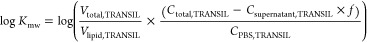
2*V*_total,TRANSIL_ represents the total volume of the sample and *V*_lipid,TRANSIL_ is the volume of added lipid known from the product certificate of analysis. *C*_total,TRANSIL_ and *C*_supernatant,TRANSIL_ are the concentrations of the test compounds in the controls and in the supernatant, respectively. The volume correction factor *f*, defined as the ratio between the PBS volume in the sample and *V*_total,TRANSIL_, was introduced to compensate for the volume effects caused by a large amount of the SSLM beads added.^44^ The density of the SSLM beads was assumed to be 2.1 g/cm^3^.^47^

Considering the structural variability of IL cations as well as the limitations of experimental methods to measure *K*_mw_ (e.g., log *K*_mw_ between approximately 1.5 and 5.5 can be measured using the standard TRANSIL Intestinal Absorption kit^43^), it is of great importance to be able to reliably predict *K*_mw_ at lower cost and with higher throughput. Therefore, experimental *K*_mw_ data for IL cations compiled from this study and other literature sources were used to develop a prediction model. Four different approaches for log *K*_mw_ prediction were investigated: (i) correlation with an octanol–water partition coefficient (log *K*_ow_), (ii) correlation with a chromatographic retention factor measured on an RP-18 column (log *k*_0_),^48^ (iii) polyparameter linear free energy relationship (pp-LFER) prediction, and (iv) COSMOmic prediction (see Texts S1 and S2). pp-LFER models developed for log *K*_ow_ and log *k*_0_ prediction of ILs were used to predict those values when no experimental values were available. Details are given in Texts S1 and S2. The root-mean-square error (RMSE) was calculated to assess the accuracy of each model prediction.

#### 2.5.2 Albumin–Water Partition Coefficients (*K*_albumin/w_)

The TRANSIL HSA binding kit (Sovicell GmbH, Leipzig, Germany) was used to assess the affinity of IL cations to human serum albumin (HSA). The kit is composed of a 96-well plate made of 12 strips with 8 wells, each strip including six wells with decreasing amounts of HSA-coated silica beads in PBS and two reference wells with PBS only. The same kit but including 2.5 times higher HSA amounts than the standard kit was used for low-affinity compounds, such as IL cations with an alkyl chain length of up to 8 carbons. Experiments were carried out in accordance with the protocol of the manufacturer. After thawing the plate, 15 μL of the compound solution, which was prepared in PBS containing a maximum of 10% DMSO, was added to each well of the plate, resulting in a final concentration of test compound of 5 μM (final DMSO < 1%). The plate was then placed on a shaker at 600 rpm and incubated at 25 °C for 20 min. After centrifugation at 10,000 rpm for 10 min, the supernatant was diluted with methanol (final methanol content ≥ 80% v/v) in glass vials and analyzed by LC/MS/MS to quantify the amounts bound to HSA. All compounds were tested in two independent runs. Dissociation constants (*K*_d_) values were calculated from the slopes of the isotherms and converted to albumin–water partition coefficients (*K*_albumin/w_) by eq 3.^49^ Given that the binding to serum albumin was far below saturation, the albumin–water partition coefficients (*K*_albumin/w_) can be calculated as a protein association constant (*K*_a_) divided by the molecular weight of albumin (approx. 67 kg/mol) as described in Endo et al.^49^ or, in our case, as a reciprocal of the product of protein dissociation constant (*K*_d_) and molecular weight of albumin using eq 3. In addition to the measured data, *K*_d_ values for seven compounds were obtained from the literature. For consistency, only *K*_d_ values measured at 25 °C and pH 7.4 were collected. If more than one data point was available for a given compound, then the mean value was used.
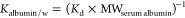
3

#### 2.5.3 Cell–Water Partition Coefficients in the Serum-Free Medium

The binding of IL cations to cells was investigated using AREc32 and AhR-CALUX cells in serum-free medium. The experimental workflow is shown in Figure S1A. To quantify the binding to cell biomass and the non-specific binding to the plate material, plates with cells (cell plate) and without cells (control plate) were prepared in parallel. The TC-treated 96-well plate (Corning-3610) was used for the tests with the AREc32 cells, while the Poly-d-Lysine (PDL) 96-well plate (Corning-354651) was used for the tests with the AhR-CALUX cell. The 19,000 cells of the AREc32 cell line and 18,000 cells of the AhR-CALUX cell line per well were seeded on a cell plate in 100 μL of assay medium (DMEM Glutamax supplemented with 10% FBS, 100 U/mL penicillin, and 100 μg/mL streptomycin). These cell plates were incubated for 24 h at 37 °C and 5% CO_2_ to allow cells to attach prior to chemical dosing.

A dosing vial (4 mL HPLC glass vial) was prepared by spiking the stock solutions of the test compounds in methanol to the assay medium. Subsequently, test solutions in the dosing vial were transferred to a 96-well deep plate (dosing plate) in duplicate by a serial dilution (the final methanol content in the test well <0.5%) to have four nominal concentrations differing by a factor of 8. For chemical dosing to the cell plate, 100 μL of the assay medium present in the cell plate was removed, and subsequently, 200 μL of test solutions were transferred from the dosing plate to the cell plate. For chemical dosing to the control plate, 200 μL solution was directly transferred from the dosing plate to the empty control plate. Both plates were then incubated for 24 h at 37 °C and 5% CO_2_. Cell confluency was measured before dosing and after 24 h of exposure using a live cell imaging system (IncuCyte S3, Essen BioScience, Ann Arbor, Michigan, USA). The remaining solution in the dosing plate used for the cell plate and the control plate was collected to quantify the initial chemical concentrations in the cell plate (*C*_cell plate_ at *t*_0h_) and in the control plate (*C*_control plate_ at *t*_0h_). After 24 h of chemical exposure, 100 μL of the supernatant from each cell plate and control plate was transferred to an HPLC vial containing methanol (the final methanol content (v/v) ≥ 80%) to quantify the concentrations in the aqueous phase in the cell plate (*C*_cell plate_ at *t*_24h_) and in the control plate (*C*_control plate_ at *t*_24h_) using LC/MS/MS.

In principle, the chemical amounts in an *in vitro* cell-based system can be described by a mass balance (eq 4) with the total amount (*n*_total_), the amounts in medium (*n*_medium_), in cells (*n*_cell_), and in a plate (*n*_plate_).
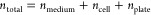
4

The chemical amounts that sorbed to the plastic plate (*n*_plate_) can be derived from changes of chemical amounts in the control plate (*n*_control plate_) over 24 h by eq 5:
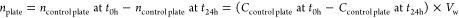
5

The chemical amounts in the medium of the cell plate at *t*_0h_ and *t*_24h_ correspond to *n*_total_ and *n*_medium_, respectively. Therefore, *n*_cell_ can be calculated by rearranging eq 4 with *n*_plate_ derived from eq 5 into eq 6
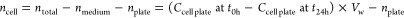
6

Since the medium in this test (PBS buffer) does not contain lipids and proteins, the chemical amounts that were quantified from the cell plate medium at *t*_24h_ (*n*_cell plate_ at *t*_24h_) can be introduced into eq 7 to calculate the *K*_cell/w_ at each concentration point.
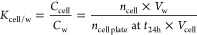
7*V*_w_ and *V*_cell_ correspond to the volume of the water (200 μL) and cells in the system. The cell volume (1.0 × 10^–5^ μL/cell for AREc32 and 3.53 × 10^–6^ μL/cell for AhR-CALUX cell line) reported in Henneberger et al.^50^ was multiplied by the mean cell number in the assay (the average between the number of seeded cells and the final cell number after 24 h of exposure, unless the final cell number was lower in which case the initially seeded amount was used) to derive *V*_cell_. Concentration points showing high non-specific binding to plastic plate (*n*_control plate_ at *t*_24h_/*n*_control plate_ at *t*_0h_) > 40% were excluded because in these cases the binding to plastic became a dominant sorptive process, resulting in an underestimation of the partitioning to cells.

#### 2.5.4 Cell Binding Experiments in the Serum-Containing Medium and Cytotoxicity

Due to the method limitations in measuring *K*_cell/w_ in the serum-free medium (e.g., substantial non-specific binding to a plastic plate occurring for hydrophobic compounds), *K*_cell/w_ was derived from the bioassay medium containing 10% FBS. Since test compounds are nonvolatile chemicals, the decrease in medium concentration during 24 h cell exposure can be attributed to cell metabolism, binding to the plastic plate, or partitioning into cells. Cell metabolism is less relevant for imidazolium ILs because both the AREc32 and AhR-CALUX were not capable of metabolizing them in our previous study.^42^ However, N11-R-1Ph Cl was metabolized by both cells. In the AhR-CALUX cells, the side chain of N11-R-1Ph Cl was oxidized to a greater extent, which resulted in reduced cytotoxicity compared to other structurally similar ILs (e.g., IM1-R Cl with the same alkyl chain length). As cell metabolism causes loss of parent compounds, which we could not quantify in this study, we did not attempt to calculate *K*_cell/w_ for N11-R-1Ph Cl in AhR-CALUX cells. Although transformation products of N11-R-1Ph Cl were also detected in the AREc32 assay, the extent of transformation was much lower and cytotoxicity remained unaffected.^42^ Therefore, the *K*_cell/w_ for AREc32 cells was calculated for N11-R-1Ph Cl. In addition, concentration losses due to plastic binding were expected to be of minor importance because of the counteracting effects of the medium serum.^28^ Nevertheless, they were evaluated by including a control plate in each experiment (described below) and were considered in the derivation of *K*_cell/w_.

The experimental workflow is shown in Figure S1B. The 10,000 cells/well for the AREc32 assay and 9000 cells/well for the AhR-CALUX assay were plated in 100 μL of assay medium on a TC-treated 96-well plate (Corning-3610) for AREc32 cell line and a Poly-d-Lysine (PDL) 96-well plate (Corning-354651) for the AhR-CALUX cell line and incubated for 24 h at 37 °C and 5% CO_2_ to let cells attach, followed by the measurement of the confluency of the cells as described before. For chemical dosing, the stock solutions in methanol were spiked into the assay medium in a 4 mL HPLC vial (dosing vial). From the dosing vials, a six-point serial dilution was carried out in duplicate in a 96-deep well plate (dosing plate). 100 μL of each well was then transferred from the dosing plate to the cell plates, leading to a final volume of 200 μL in each well (MeOH < 0.5%). A control plate without cells was prepared in analogy to the cell plate to evaluate the chemical losses due to the plate binding. After the cell and control plates were incubated for another 24 h at 37 °C and 5% CO_2_, the cell confluency was measured again in the cell plate and used to determine the % inhibition of cell viability by comparing the confluency of the exposed cells to that of the unexposed cells. It was then used to draw a concentration–response curve (CRC) and determine the concentrations causing 10% cytotoxicity (see the Section 2.7).

The initial medium concentrations in the cell plate (*C*_total medium_ at *t*_0h_) and in the control plate (*C*_control plate_ at *t*_0h_) were determined by measuring the concentrations of the dosing solutions left in the corresponding dosing plates. At the end of the test, 50 μL from each well of the cell plate and the control plate were transferred to an HPLC vial to determine the total medium concentration at *t*_24h_ in the cell plate (*C*_total medium_ at *t*_24h_) and in the control plate (*C*_control plate_ at *t*_24h_). 80% (v/v) methanol was added to the collected samples to extract chemicals associated with the medium serum by precipitating protein. After centrifugation at 10,000 rpm for 10 min, the supernatants were collected in HPLC vials for LC/MS/MS measurement. *n*_plate_ was derived from the concentration changes in the control plate by eq 5 using the volume of the total medium (*V*_medium_) of 200 μL instead of *V*_w_ and introduced into eq 8 to derive the chemical amounts associated with cells (*n*_cell_).
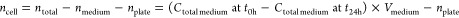
8*V*_cell_ is the volume of cells in a given assay and is calculated as described before.

*K*_cell/w_ is the ratio of the concentration in cells (*C*_cell_) to the freely dissolved concentration in the medium (*C*_free_) and can be calculated by eq 9 using the freely dissolved fraction in the medium (*f*_free_).
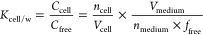
9*f*_free_ can be modeled using the volume fraction of water (Vf_w,medium_), protein (Vf_protein,medium_), lipid (Vf_lip,medium_) in medium, volume of cells (*V*_cell_) and medium (*V*_medium_) with corresponding partition coefficients in eq 10. Vf_w,medium_, Vf_protein,medium_, and Vf_lip,medium_ for test medium were taken from Qin et al.^51^ and are listed in Table S4.
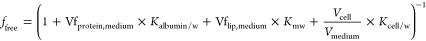
10*K*_cell/w_ can then be derived from eq 11 by combining eqs 9 and 10.
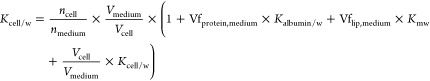
11

If the contribution of cell partitioning to *f*_free_ is negligible, which is often the case when the medium is supplemented with FBS, eq 11 can be simplified to eq 12. Since eqs 11 and 12 provided the same *K*_cell/w_ within this study, eq 12 was preferably used.
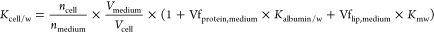
12*n*_total_ corresponds to the chemical amounts quantified from *C*_total medium_ at *t*_0h._*n*_medium_ is obtained from eq 4 by subtracting *n*_cell_ and *n*_plate_ from *n*_total_.

### 2.6 Determination of the Unbound Fraction in the Assay Medium Using Rapid Equilibrium Dialysis (RED)

The unbound fraction (*f*_u_) of the IL cation in the assay medium was evaluated using the Rapid Equilibrium Dialysis (RED) device (Thermo Fischer Scientific, Waltham, MA, USA) in accordance with the product protocol. Briefly, solutions of IL cation in the DMEM medium containing 10% FBS (% methanol <0.5%) were incubated for 24 h at 37 °C in an HPLC vial to reach equilibrium with the medium constituents. Subsequently, 300 μL of this solution and 550 μL of PBS were transferred to the sample and buffer chambers of the RED inserts, respectively. The solution remaining in the HPLC vial was used to quantify the total concentration added to the RED system (*C*_total,RED_) and the corresponding chemical amounts (*n*_total,RED_). After being sealed, the RED plate was incubated at 37 °C for 6 h at 600 rpm on an orbital shaker. Note that a preliminary kinetic test with three time points (6, 8, and 16 h) was performed with N11-12-1Ph Cl and IM1-16 Cl, and the dialysis system reached equilibrium within 6 h. After incubation, 100 μL of the sample chamber (*C*_medium,RED_) and buffer chamber (*C*_PBS,RED_) were transferred to an HPLC vial containing methanol (final methanol content (v/v) ≥ 80%) and centrifuged at 10,000 rpm for 10 min to separate the protein. The assay was performed in triplicate at three to four different concentrations, covering the test ranges used in the bioassays that could still be reliably quantified. IM1-8 Cl and N11-16-1Ph Cl were tested only at two concentration points because of their low affinity (IM1-8 Cl, little difference between medium bound and unbound fractions) or high affinity (N11-16-1Ph Cl, unbound fraction close to LOQ).

The fraction of IL cations not bound to medium constituents (*f*_u_) was calculated by dividing the concentrations in the buffer chamber (*C*_PBS,RED_) by the concentrations in the medium chamber (*C*_medium,RED_) using eq 13.
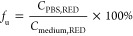
13

A one-way analysis of variance (ANOVA) for a linear trend was carried out to evaluate the linearity between the *f*_u_ and concentration. The recovery was evaluated by comparing the sum of the compound amounts in medium (*n*_medium,RED_) and buffer chamber (*n*_PBS,RED_) with the total amount (*n*_total,RED_) (eq 14).
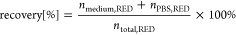
14

The *f*_u_ was then multiplied by *C*_total medium_ at *t*_24h_, the medium concentrations measured at the end of 24 h exposure in Section 2.5.4, to derive the freely dissolved concentration in the assay medium (*C*_free,medium_) (eq 15). Each *C*_total medium_ at *t*_24h_ was converted into *C*_free,medium_ to draw the CRC based on *C*_free,medium_.
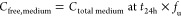
15

### 2.7 Cytotoxicity Data Processing

Cytotoxicity was expressed as % inhibition of cell viability compared to unexposed cells from the confluency measurements. Inhibitory concentrations causing 10% cell inhibition (IC_10_) and corresponding standard error (SE) were calculated from the slope of the linear portion of the concentration–response curve (CRC) using eqs 16 and 17.^52^
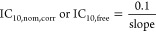
16
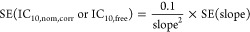
17

To account for the concentration loss due to plate binding, the nominal concentrations were corrected in eq 18 with differences between *n*_total medium_ at *t*_0h_ and *n*_plate_ (*C*_nom,corr_) that were quantified in the test for *K*_cell/w_ determination in the serum-containing medium.
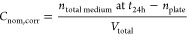
18

Therefore, the nominal concentrations causing 10% cytotoxicity (IC_10,nom,corr_) were derived from the CRC based on *C*_nom,corr_ (eq 16) while the cytotoxicity in the freely dissolved concentrations (IC_10,free_) was derived from the CRC based on *C*_free,medium_ (eq 15).

### 2.8 Mass Balance Model for Predicting Distribution of IL Cations and Estimating Cell Membrane Concentration

The *C*_free,medium_ can be predicted from *C*_nom,corr_ and *f*_free_ in eq 19 from binding to cells and medium components, assuming that protein and lipid are the dominant sinks for a chemical in medium and that interactions with protein and lipids are defined by distribution coefficients *K*_albumin/w_ and *K*_mw_.^33,35^ The modeled *C*_free,medium_ was then compared with the measured *C*_free,medium_ (eq 15) to verify the accuracy of the model.
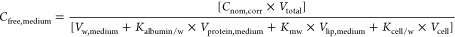
19*K*_cell/w_ was also modeled using eq 20 with the volume fractions of proteins, lipids, and water of the cells (Vf_protein,cell_, Vf_lip,cell_, and Vf_w,cell_) for the AREc32 and AhR-CALUX assay^51^ (Table S5) and compared with the experimental *K*_cell/w_.
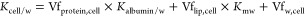
20

The experimentally determined IC_10,free_ and *K*_mw_ were used to derive the concentrations in the cell membrane (IC_10,membrane_). Given that internal and external freely dissolved concentrations are equal, the cellular membrane concentration IC_10,membrane_ can be directly derived from multiplying IC_10,free_ by *K*_mw_ (eq 21).^40^
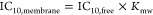
21

## 3 Results and Discussion

### 3.1 Determination of Critical Micelle Concentrations (CMC)

As many IL cations have structural elements that are characteristic of ionic surfactants, i.e., a charged (polar) headgroup and an apolar chain, they can form micelles in aqueous solutions upon exceeding a concentration threshold known as the critical micelle concentration (CMC).^41^ Since micelle formation influences the partitioning of the surfactant into bilayers,^53^ the partition coefficient of the surfactant monomer should be determined below the onset of micellization. Therefore, the CMCs of the long-chain homologues were measured. The measured CMCs are summarized in Table S6, together with literature values for IM1-10 Cl and IM1-12 Cl. The CMC value of IM1-12 Cl measured by isothermal titration calorimetry (2.9 mM)^54^ and by a fluorescent probe (1.9 mM) in this study (Table S6) were comparable, demonstrating the robustness of the fluorescent probe-based approach. The CMC decreased with the side chain elongation for both IM1-R Cl and N11-R-1Ph Cl, confirming that increasing the size of the hydrophobic domain of the IL cations favors micelle formation (Table S6 and Figure S2).^36^ Nominal concentrations were used to derive CMC as significant binding to plastic plates occurred only at concentrations above the CMC for all compounds tested except IM1-16 Cl, for which 50% losses were found at concentrations (40 μM) below the CMC, and N11-16-1Ph Cl, for which losses reaching up to 70% were observed in the serum-free medium at the CMC (details in Section 3.2). Therefore, the CMCs of these two compounds should be treated as approximate CMC. Nevertheless, a linear relationship between the logarithm of the CMC and the number of carbon atoms in the alkyl chain has been reported for surface active agents^55^ and has also been observed for a homologous series of the IL cations (Figure S2H), allowing the estimation of CMC values in the homologous series. The CMC for the IL cations with a side chain shorter than that of decyl was extrapolated from this linear relationship. Additionally, it was observed that the CMC values in PBS were approximately 1 order of magnitude lower than those measured in pure water,^36^ confirming the dependence of the CMC on the salinity of the solution and the need to know the CMC values in the test medium used for toxicity tests. Therefore, in all subsequent experiments, the test concentration range was set below the CMC measured in PBS to avoid micelle formation.

### 3.2 Chemical Sorption to Plastic Plates and the Sorption-Reducing Effect of Medium Serum

Chemical sorption to plastic materials can significantly deplete the test concentration in *in vitro* assay systems, which are typically performed in multiwell plastic plates, often made up of polystyrene. The loss of test chemicals resulting from plastic plate sorption can be counteracted by the medium serum, as the desorption of the chemicals from the medium serum to the medium is faster than the transfer to the plastic.^30,31^ This phenomenon has been termed “serum-mediated passive dosing (SMPD)” because the medium serum can act as a passive dosing reservoir.^28^ However, Groothuis et al.^56^ showed that the reduction of concentrations of hydrophobic cationic surfactants was still significant at low nominal concentrations, even in media containing 10% serum albumin. Therefore, the chemical exposure stability in a multiwell plate setup and the ability of serum to counteract test compound losses due to plastic sorption were evaluated in a wide range of hydrophobicity and nominal concentrations using IM1-R Cl and N11-R-1Ph Cl with varying alkyl chain lengths.

**Figure 1:**
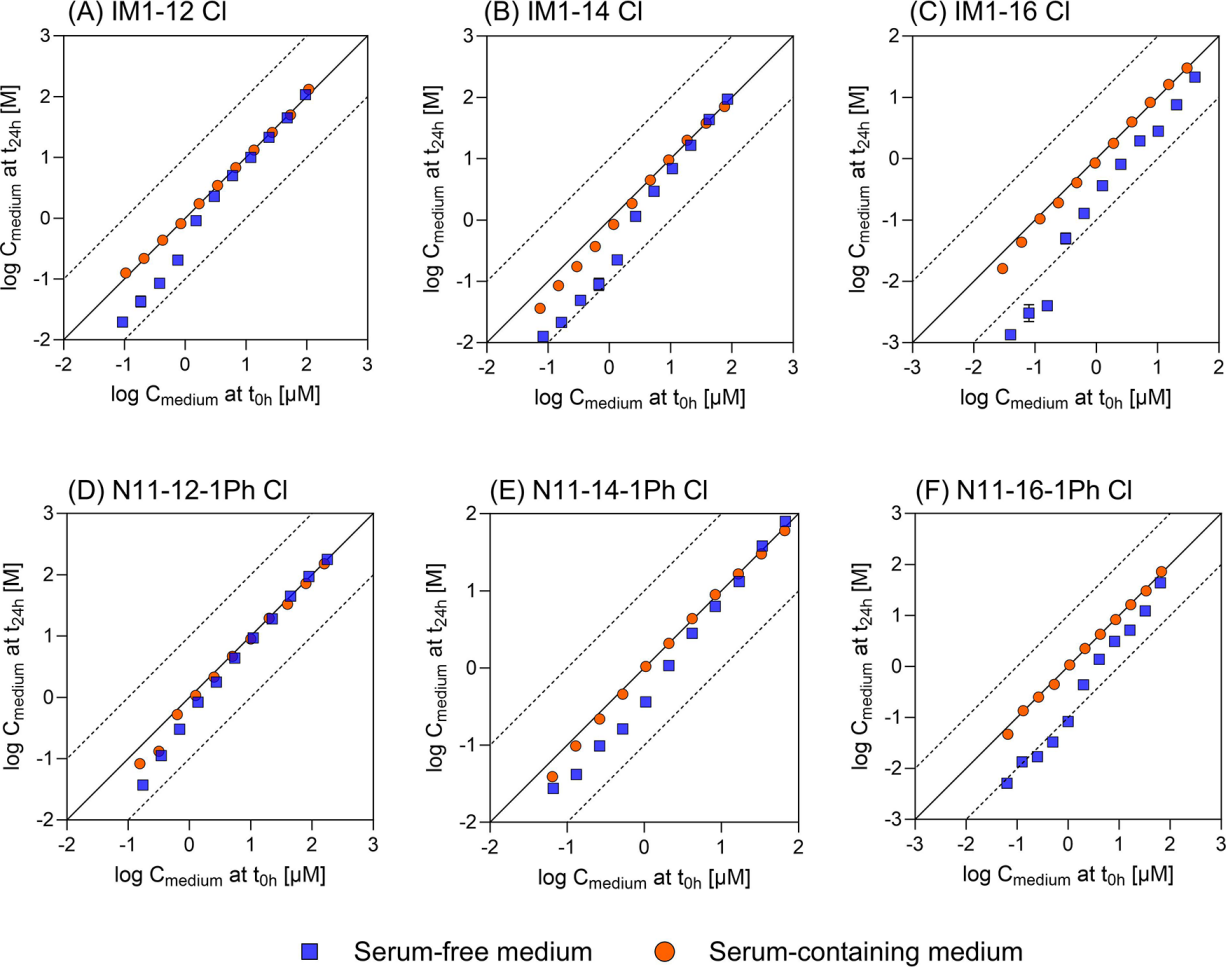
Differences between the initial medium concentration (*C*_medium_ at *t*_0h_) and the medium concentration after 24 h of incubation (*C*_medium_ at *t*_24h_) for cations of ionic liquids (ILs) in a 96-well plate. Sorption to the plastic plate was evaluated in the serum-free medium (blue square) or the serum-containing medium (orange circle). The solid line indicates the scenario where plate binding does not cause changes in medium concentrations (*C*_medium_ at *t*_0h_ = *C*_medium_ at *t*_24h_). The dotted lines correspond to the 1 order of magnitude deviation from the solid line.

In the serum-free medium, the reduction of the medium concentration began to occur for the IL cations with an alkyl chain of ≥12 carbons, reaching the greatest concentration losses for the IL cations carrying the longest side chain (blue squares in Figure 1). It confirms that plastic binding is predominantly influenced by the hydrophobicity of the IL cation. The sorption was concentration-dependent with hardly any concentration losses at high concentrations (saturation of sorption sites) while concentration losses reached 98% for IM1-16 Cl at the lowest concentrations, resulting in *C*_medium_ at *t*_24h_ being more than 1 order of magnitude lower than *C*_medium_ at *t*_0h_ (Figure 1). Two regions with different slopes were observed in the sorption isotherms for the majority of the IL cations (Figure S3). This phenomenon has been reported for the adsorption of ionic surfactants on oppositely charged sites and can be explained by the “four-region theory”.^57,58^ At low adsorption densities, the electrostatic attraction between the charged surface and the surfactant ions is the main driver of the adsorption (Region I); the lateral interaction between hydrophobic chains facilitates adsorption, which is visible as the increased slope in this region (region II). As the adsorption density increases, the hydrophobic interaction of side chains starts dominating the adsorption, and second layers of ionic surfactant are formed on the previously adsorbed layer with surfactant head groups facing the solution (region III), finally, the adsorption isotherm reaches a plateau region where micelles are formed (region IV).^37^ A decrease in the slope of the isotherms in Figure S3 may indicate a transition from Region II to Region III while regions I (for all IL cations) and IV (for N11-14-1Ph Cl and N11-16-1Ph Cl) appear absent due to the limited concentration range tested.

In the presence of 10% FBS in the medium, the changes in medium concentrations over 24 h remained within 1 order of magnitude compared to *C*_medium_ at *t*_24h_ (orange circles in Figure 1), indicating that the medium serum largely counteracted the concentration depletion resulting from plastic binding. The greatest chemical loss was observed for IM1-14 Cl and IM1-16 Cl (Figure 1B,C) reaching up to 50% at the lowest test concentration. For these compounds, disregarding plastic sorption leads to an underestimation of toxic effects when cytotoxicity is reported only in terms of nominal concentration. For example, the IC_10_ of IM1-16 Cl in the AhR-CALUX assay was observed at the concentration level at which the chemical loss was greater than 30%.^42^ Therefore, the chemical adsorption on the plate (*n*_plate_) in a control plate without cells was quantified in parallel to assays for cytotoxicity and used to correct the nominal concentration (eq 18).

### 3.3 Determination of Membrane Lipid–Water Partition Coefficients

The *K*_mw_ values of IL cations were determined either experimentally or by prediction models (e.g., correlation with log *K*_ow_ or log *k*_0_ as well as pp-LFER, and COSMOmic predictions in Text S1 and Text S2). In addition to the 17 IL cations that were tested in this study, the experimental *K*_mw_ of 12 IL cations were compiled from the literature. The *K*_mw_ values for IM1-R Cl (R:8–16) and N11-R-1Ph Cl (R:10–16) are listed in Table 1 and the results for all other compounds are given in Table S7. The sorption isotherms for 6 cations of ILs obtained using the TRANSIL Intestinal Absorption beads are depicted in Figure S4. The log *K*_mw_ increased monotonously with the elongation of the alkyl side chain in all homologous groups (Figure S5) as reported also for ionic surfactants.^59,60^ Previously, a non-linear relationship between the log *K*_mw_ and the chain length was observed for IL homologues with long side chains when tested at a ratio of lipid molecules of SSLMs to IL cations sorbed on the lipid of less than 60. However, when the ratio was sufficiently high (>60), this leveling-off was no longer observed, suggesting that the side chain cut-off in *K*_mw_ is an experimental artifact. It is conceivable that upon reaching a high density of the IL cations in the lipid membrane, the membrane becomes saturated and unstable, which may alter the binding behavior.^44,45^ Accumulation of charged molecules like IL cations or ionic surfactants in the membrane may also cause charge buildup and result in electrostatic repulsion between the sorbate molecules. IM1-12 Cl was tested at various lipid/[IM1-12]^+^ cation molar ratios (4–123) by increasing the concentration of IM1-12 Cl from 0.5 to 50 μM at fixed lipid volume (Figure S6). At a molar ratio below 51, the isotherm levels off, resulting in a decrease of log *K*_mw_ from 4.73 to 3.93 when the molar ratio decreased from 51 to 4 (Figure S6).

**Table 1:** Partition Coefficients of IM1-R Cl (R:8-16) and N11-R-1Ph Cl (R:10-16) for Membrane Lipid–Water (*K*_mw_), Albumin–Water (*K*_albumin/w_), and Cell–Water (*K*_cell/w_)

			log *K*_cell/w_ [*L*_w_/*L*_cell_]	
chemical	log *K*_mw_ [*L*_w_/*L*_lip_]^a^	log *K*_albumin/w_ [*L*_w_/*L*_protein_]^a^	AREc32	AhR-CALUX
IM1-8 Cl	2.06^b^	0.47^e^	1.99 ± 0.09^h^	2.19 ± 0.14^h^
IM1-10 Cl	3.58 ± 0.07	1.92 ± 0.03	2.16 ± 0.20^i^	3.48 ± 0.29^i^
IM1-12 Cl	4.44 ± 0.03	2.67^f^	3.08 ± 0.15^i^	3.93 ± 0.18^i^
IM1-14 Cl	5.56 ± 0.09	3.08 ± 0.04	4.11 ± 0.28^i^	5.08 ± 0.13^i^
IM1-16 Cl	6.66^c^	3.70 ± 0.02	5.39 ± 0.20^i^	5.76 ± 0.15^i^
N11-10-1Ph Cl	4.01^d^	2.13 ± 0.03	2.41 ± 0.23^i^	
N11-12-1Ph Cl	5.25 ± 0.07	3.08 ± 0.02	4.27 ± 0.18^i^	
N11-14-1Ph Cl	5.92^c^	3.65 ± 0.02	4.73 ± 0.18^i^	
N11-16-1Ph Cl	6.94^c^	4.47^g^	5.90 ± 0.14^i^	

a Partition coefficients log *K*_mw_ and *K*_albumin/w_ measured in this study.

b log *K*_mw_ from Stolte et al.^46^

c log *K*_mw_ predicted by COSMOmic.

d log *K*_mw_ from Timmer and Droge.^59^

e average of log *K*_albumin/w_ from Yan et al. (0.22)^61^ and Huang et al. (0.72).^62^

f log *K*_albumin/w_ from Zhou et al.^63^

g log *K*_albumin/w_ extrapolated from homologous series.

h log *K*_cell/w_ measured in the serum-free medium.

i log *K*_cell/w_ measured in the serum-containing medium.

To fill the gap in *K*_mw_ data, four different prediction models were evaluated (see Texts S1 and S2 together with Tables S8 and S9 for the analysis of model performance). COSMOmic showed the highest accuracy in predicting the *K*_mw_ of the IL cations among the four models investigated (Figure S7). Therefore, the *K*_mw_ values predicted by COSMOmic were preferably used in the following sections when experimental data were not available (i.e., IM1-16 Cl, N11-14-1Ph Cl, or N11-16-1Ph Cl in Table 1).

### 3.4 Albumin–Water Partition Coefficients (*K*_albumin/w_)

*K*_albumin/w_ values were experimentally determined for 13 IL cations in this study while additional experimental values of 6 IL cations were collected from the literature (listed in Tables 1 and S7). The sorption isotherms for five of the IL cations were measured using the standard TRANSIL HSA Binding Kit, and for eight of the IL cations, they were measured using the TRANSIL HSA Binding Kit for low binders, as shown in Figures S8 and S9, respectively. IL cations with a short alkyl chain (*C* ≤ 4) showed negligible sorption to human serum albumin; therefore, the *K*_albumin/w_ could only be reliably quantified for compounds substituted with a sufficiently long side chain (*C* ≥ 6). The log *K*_albumin/w_ values increased linearly with the side chain length within the homologue series for N11-R-1Ph Cl (*R*^2^ = 0.98) and IM1-R Cl (*R*^2^ = 0.98) (Figure S10). This linear relationship was used to extrapolate the log *K*_albumin/w_ for N11-16-1Ph Cl due to the limitations of experimental methods for such strongly binding compounds (binding fraction >99.5%), yielding log *K*_albumin/w_ = 4.47 (Table 1). Generally, N11-R-1Ph Cl showed approximately 0.4 log unit higher affinity to albumin compared to IM1-R Cl bearing the same alkyl chain. An average increase of log *K*_albumin/w_ by 0.36 and 0.38 log units per CH_2_ segment for IM1-R Cl and N11-R-1Ph Cl, respectively, was noted.

**Figure 2:**
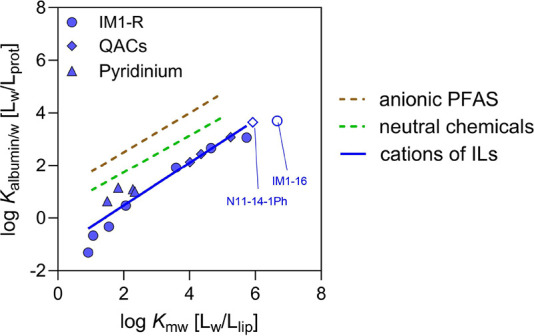
Linear relationship of experimental membrane lipid–water partition coefficients (log *K*_mw_ in *L*_w_/*L*_lip_) against measured albumin–water partition coefficients (log *K*_albumin/w_ in *L*_w_/*L*_protein_) for IL cations carrying various head groups (e.g., imidazolium (IM1-R), quaternary ammonium compounds (QACs including N11-R-1Ph), and pyridinium) shown with different symbols. N11-14-1Ph and IM1-16 (empty symbols) were not included in the regression (eq 22) since their *K*_mw_ values were predicted by COSMOmic (Table 1). For comparison, linear relationships of *K*_mw_–*K*_albumin/w_ for neutral chemicals^49^ and anionic PFAS^51^ were plotted as dotted lines. Since *K*_albumin/w_ of anionic PFAS are concentration-dependent, *K*_albumin/w_ of anionic PFAS at the concentration level causing 10% cell viability inhibition (IC_10_) were used.^51^

The log *K*_albumin/w_ correlated linearly with the measured log *K*_mw_ for 14 IL cations, giving eq 22 (Figure 2). Note that the *K*_mw_ for IM1-16 Cl, N11-14-1Ph Cl was predicted by COSMOmic.^42^
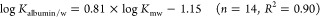
22

A linear relationship between *K*_albumin/w_ and *K*_mw_ was previously reported for neutral chemicals^64^ and anionic PFAS^51^ (Figure 2). Regression models showed similar slopes for neutral chemicals and anionic PFAS but different intercepts depending on the chemical species, confirming that the affinity for serum proteins is not a simple function of hydrophobicity and can be dependent on charges or chemical classes (Figure 2). For the IL cations, the *K*_mw_ is up to 3 orders of magnitude higher than *K*_albumin/w_. A similar trend has previously been observed for quaternary ammonium compounds carrying a long alkyl chain, which was attributed to their structural similarity to phospholipids.^33^ In contrast, anionic compounds were found to bind more strongly to serum albumin than to lipids.^65^ Serum albumin is known to have at least three high-affinity binding sites for anionic compounds.^66^ Since the binding data of anionic PFAS used in Figure 2 were obtained at the IC_10_ level where binding site saturation was not observed, the specific interaction at the high-affinity binding sites of serum albumin may account for the observed high *K*_albumin/w_ of anionic PFAS.

### 3.5 Determination of Cell–Water Partition Coefficients (*K*_cell/w_)

The cell–water partition coefficients (*K*_cell/w_) were determined in the AREc32 and AhR-CALUX cell lines in two types of medium: serum-free and serum-containing medium. Due to the significant binding to the plastic plate (> 40%), the very hydrophobic IL cations (i.e., IM1-14 Cl, IM1-16 Cl, and N11-16-1Ph Cl) were excluded from the data analysis in the test with serum-free medium. Several concentration points for other less hydrophobic IL cations (i.e., all concentration points for IM1-12 Cl or the lowest concentration for N11-10-1Ph Cl in the AhR-CALUX cell) were additionally omitted when the medium concentration was reduced by more than 40% in a control plate. To minimize the non-specific binding to a plastic plate interfering with the determination of *K*_cell/w_, the measurements of *K*_cell/w_ were also conducted in the bioassay test medium containing serum (10% FBS). Given the low volatility and the absence of cell metabolism of IL cations, *n*_cell_ can be calculated from MBM (eq 8) and used to derive the corresponding *K*_cell/w_ by eq 12. Since IM1–8 Cl did not show significant differences in medium concentrations over 24 h of cell exposure in both assays, which indicates low cell affinity, its *n*_cell_ was not calculated. The *K*_cell/w_ of all four N11-R-1Ph Cl in the AhR-CALUX cells were not measured in the serum-containing medium because this cell line is capable of metabolizing N11-R-1Ph Cl to a substantial extent,^42^ resulting in the loss of chemicals, thereby reducing concentrations. Since the decreased concentration resulting from cell metabolism cannot be distinguished from the loss caused by cell partitioning, the determination of *K*_cell/w_ by eq 12 can lead to an overestimation.

The observed tendency for *K*_cell/w_ values to be generally lower in the serum-free medium than in the serum-containing medium may be attributed to the slower cellular uptake in the absence of FBS (Figure S11 for AREc32 cells and Figure S12 for AhR-CALUX cells). In a recent work by Fischer et al., the time required to reach a steady state in the cellular uptake of a cationic chemical (*N*,*N*,*N*-trimethyl-4-(6-phenyl-1,3,5-hexatrien-1-yl)phenylammonium *p*-toluene sulfonate) in AREc32 cells increased from 7.5 to 16.4 h when the medium FBS concentration decreased from 10 to 0.5%, indicating that the medium FBS facilitates uptake into cells.^67^ Considering that charged chemicals generally require a longer time to be taken up by cells than neutral organic chemicals,^67^ it is conceivable that the absence of FBS in the medium impedes the attainment of equilibrium between the medium and viable cells, resulting in lower apparent partition coefficients. In this context, only the *K*_cell/w_ values obtained from the serum-containing medium were used. The *K*_cell/w_ of IM1-8 Cl measured in the serum-free medium was exceptionally included for both assays since this compound is expected to reach equilibrium in cellular uptake even without medium serum due to its small molecular size.

**Figure 3:**
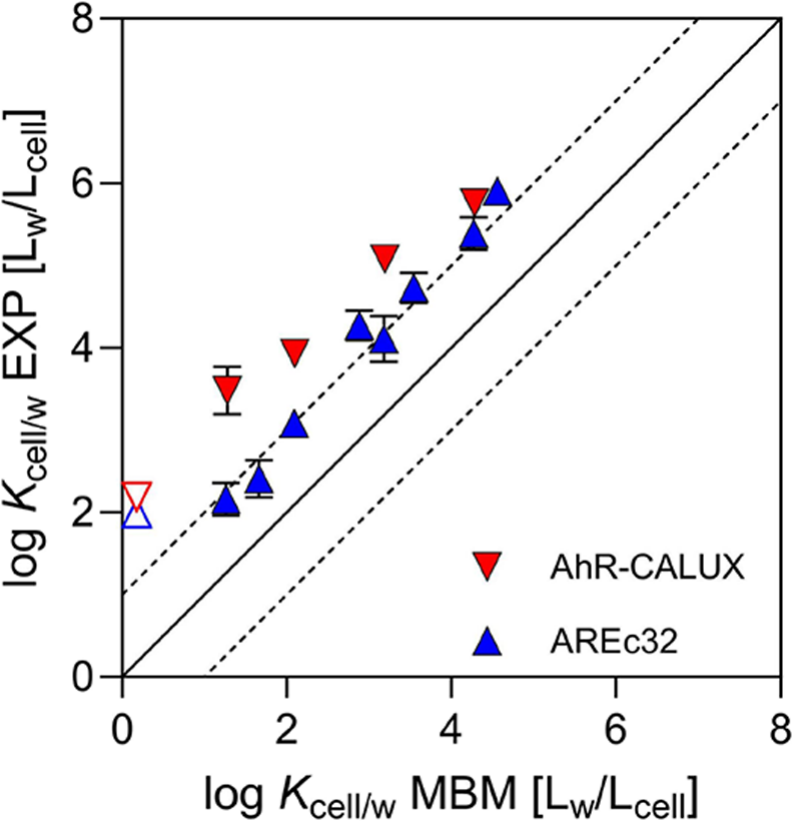
Experimental cell–water partition coefficient (log *K*_cell/w_) in comparison with log *K*_cell/w_ predicted by a mass balance model (MBM) using eq 20. The blue triangles indicate the (*K*_cell/w_) derived from the AREc32 cell line, and the red inverted triangles correspond to the values from the AhR-CALUX cell line. The open symbols indicate the *K*_cell/w_ values of IM1-8 Cl, which were measured in the serum-free medium. The black solid line denotes an ideal agreement between the MBM prediction and the experimental data, and the dotted lines correspond to a deviation of one log unit from the model prediction.

The experimental log *K*_cell/w_ measured at four to six concentrations were averaged (Table 1) and compared with those predicted by MBM (eq 20). The affinity of the IL cations for cells is higher than MBM prediction (Figure 3) and also different between cell lines (RMSE for the AREc32 cells was 1.19 in log unit, and that for the AhR-CALUX cells was 1.90 in log unit). Using phosphatidylcholine (POPC) and serum albumin as sole surrogates for cells in the model may not be sufficient to represent the diversity of biomolecules found in the cells and their interactions with ionic compounds. For instance, the type of phospholipids may influence the affinity for biological membranes. It has been shown that IL cations interact more with membranes composed of a mixture of POPC and phosphatidylglycerol (PG) (8:2) than those made of a mixture of POPC, PG, and cholesterol (6:2:2),^68^ or with 100% dipalmitoylphosphatidylglycerol (DPPG) than 100% dipalmitoylphosphatidylcholine (DPPC) or pure cholesterol.^69^ Thus, the *K*_mw_ values derived from POPC bilayers may not accurately reflect the potential interactions of IL cations with the lipids present in cell membranes. Moreover, the negatively charged outer membrane of living cells may contribute to the enhanced interactions with cations compared to neutral compounds as shown in the study with cationic molecules and charged bilayers.^70,71^ Additionally, *K*_albumin/w_ may not be an accurate proxy for the cell protein affinity. In fact, the proteins of mammalian cells have more of a nature of structural protein than serum proteins.^72^ For anionic compounds that possess a stronger affinity for serum albumin than for structural proteins, using only *K*_albumin/w_ in the MBM leads to an overestimation of *K*_cell/w_.^50^ However, for organic cations, the affinity to structural proteins and serum protein is similar.^73^ Hence, using *K*_albumin/w_ is rather unlikely to be the reason for the deviation between the predicted and experimental *K*_cell/w_ values.

Interestingly, the *K*_cell/w_ values were generally higher in the AhR-CALUX cells than in the AREc32 cells (Figure 3). Plotting the experimental log *K*_cell/w_ against log *K*_mw_ shows a good linear relationship for both cell lines with a similar slope, while an intercept for the AhR-CALUX cells was up to 1.2 log units higher than the AREc32 cells (Figure S13). This may account for the lower IC_10,nom_ of IL cations in the AhR-CALUX than in the AREc32 assay reported previously.^42^ In the study of Bae et al., 20 out of 28 IL cations paired with a halide had IC_10,nom_ in the AhR-CALUX assay at least 1 order of magnitude lower than in the AREc32 assay (maximum difference up to 600-fold).^42^ Because of the higher affinity of the IL cations for the AhR-CALUX cells compared to the AREc32 cells, lower nominal concentrations of the IL cations might be sufficient to yield the same level of toxic effects in the AhR-CALUX.

### 3.6 Quantification of Freely Dissolved Concentration (*C*_free,medium_) in Bioassays

The unbound fraction (*f*_u_ (%)) in the bioassay medium containing 10% FBS was determined using a rapid equilibrium dialysis (RED) device. The results were used to validate the accuracy of the MBM prediction since the freely dissolved fraction in the cell-free medium can be calculated by multiplying *f*_u_ (%) with the total medium concentration (eq 15). A preliminary kinetic test showed that the *f*_u_ (%) of N11-12-1Ph Cl and IM1-16 Cl was constant between 6 and 16 h (*p* > 0.05 in ANOVA), suggesting that both compounds reached equilibrium in less than 6 h in the RED device (Figure S14). Since the equilibrium time in a RED system is dependent on the molecular weight of a chemical,^74^ it was assumed that other compounds with similar or lower molecular weights than N11-12-1Ph Cl and IM1-16 Cl would also reach the equilibrium within 6 h.

The resulting *f*_u_ (%, eq 13) for all IL cations as a function of the total concentrations added in the RED system (*C*_total,RED_ μmol/L) is illustrated in Figure S15. The experimental and modeled *f*_u_ (%) values together with the corresponding recoveries are listed in Table S10. All chemicals exhibited good recoveries (>75%) within the test concentration range (Table S10). The lower *f*_u_ (%) was observed for the IL cations with a longer side chain within the same homologous series due to the higher affinity for serum constituents. In the case of N11-16-1Ph Cl, with the highest affinity for albumin and lipids, less than 0.01% was present in the freely dissolved form.

A concentration-dependent relationship of *f*_u_ (%) was observed for IM1-14 Cl, N11-10-1Ph Cl, and N11-12-1Ph Cl (Figure S15), with *f*_u_ (%) values increasing as *C*_total,RED_ increased (*p* < 0.05 in ANOVA test for linear trend). This may be attributed to the progressing saturation of binding sites in serum albumin.^69^ However, the differences in *f*_u_ (%) were less than 2-fold within the tested concentration range, which is of low practical importance. Therefore, the mean values of *f*_u_ (%) were used for comparison with the MBM prediction. In general, the experimental *f*_u_ (%) agreed well with the *f*_u_ (%) predicted by MBM (Table S10), with differences within 1 order of magnitude. The exception was N11-16-1Ph Cl with the experimental *f*_u_ (%) being 70 times lower than the prediction. Considering that the log *K*_mw_ and log *K*_albumin/w_ of N11-16-1Ph Cl used in the MBM calculation are extrapolated from a correlation in homologous series (Table 1), the predicted *f*_u_ of this compound bears rather high uncertainty. Moreover, the very low test concentration (<10 μM) of this highly medium-binding compound resulted in *C*_PBS,RED_ close to the limit of quantification and a relatively large error (coefficient of variation >50%). Therefore, the large deviation for N11-16-1Ph Cl may stem from either a prediction error, analytical artifacts, or both.

The *C*_free,medium_ values in the AREc32 and AhR-CALUX assays were determined using the experimental *f*_u_ (eq 15) and compared with the MBM prediction (eq 19). In the case of four N11-R-1Ph Cl in the AhR-CALUX assay, the *C*_free,medium_ was not predicted because the experimental *K*_cell/w_ values were not available. The IC_10,free_ values (Table S11) were derived from the CRCs based on the experimental *C*_free,medium_ (eq 15) and compared with the IC_10,nom,corr_ values that were derived from CRCs based on the *C*_nom,corr_ (full CRCs can be found in Figure S16). Note that the IC_10,nom_, which was derived from nominal concentrations without correction for plate binding losses, was also listed in Table S11, demonstrating that disregarding the impacts of plate binding on *C*_nom_ results in a difference of less than an order of magnitude in IC_10_ values. Therefore, in this study the impacts of plate binding can be considered low for cytotoxicity reporting, but IC_10,nom,corr_ was still used for higher accuracy. The experimental *C*_free,medium_ values for IL cations generally agreed well with the MBM prediction in both assays, with a deviation within one log unit (Figure S17). An exception was N11-16-1Ph Cl, for which the quantification of *f*_u_ (%) was based on extrapolated values. Nevertheless, the overall good agreement between the experimental *C*_free,medium_ and MBM prediction indicates that the existing MBM built on the experimental partition coefficients can reliably predict the *C*_free,medium_ of the IL cations in *in vitro* cell assays. Including the experimental *K*_cell/w_ in eq 19 yielded a better prediction of IC_10,free_ compared to IC_10,free_ predicted using the modeled *K*_cell/w_ (Figure S18); the RMSE decreases from 0.80 to 0.70 in the AREc32 and 0.59 to 0.46 in the AhR-CALUX assay, respectively. Although using the *K*_cell/w_ modeled from *K*_mw_ and *K*_albumin/w_ can still reasonably predict IC_10,free_ (RMSE in log unit <1.0), the measured cell affinity can bring a more accurate MBM prediction, especially for hydrophobic compounds.

**Figure 4:**
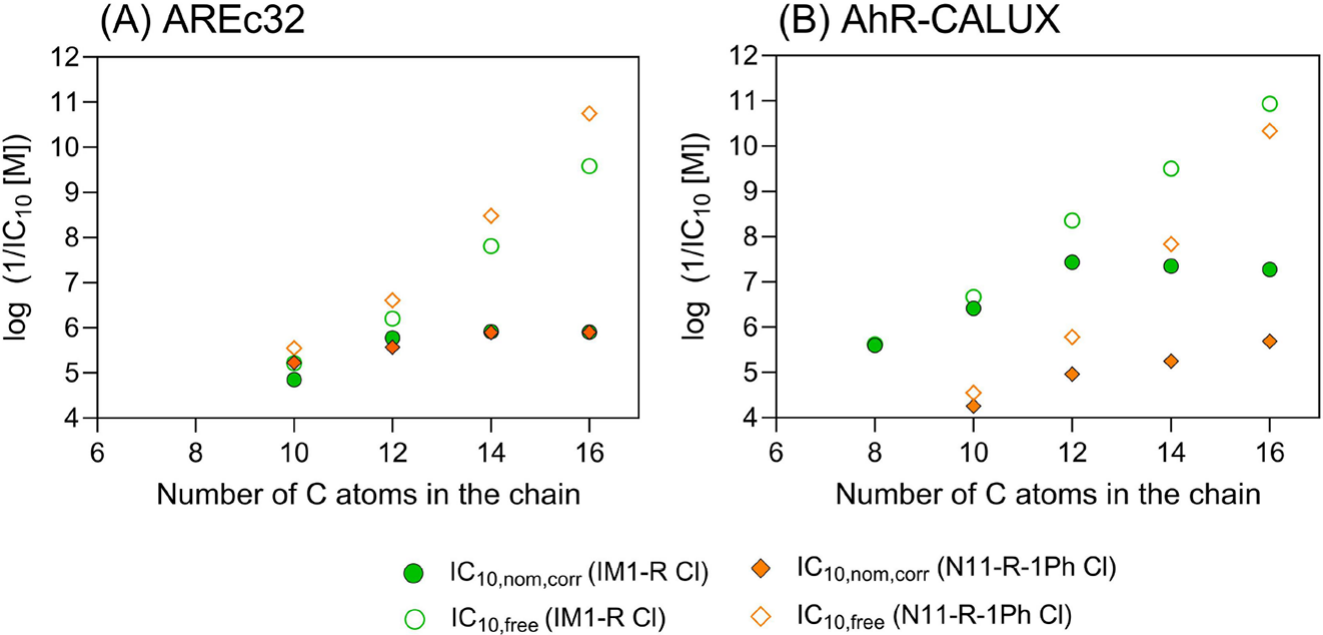
Cytotoxicity of IM1-R Cl (R:8-16) and N11-R-1Ph Cl (R:10-16) in the AREc32 (A) and AhR-CALUX (B) cell lines was plotted against the carbon number in the chain. The nominal concentration corrected with the plate binding-based IC_10_ (IC_10,nom,corr_) was represented in the filled symbols, while the freely dissolved concentration-based IC_10_ (IC_10,free_) was marked in the empty symbols. No cut-off in cytotoxicity can be observed when effective concentrations are expressed as freely dissolved concentrations.

Figure 4 illustrates how IC_10,free_ differs from IC_10,nom,corr_ depending on the structure of the IL cations. For IM1-8 Cl carrying the shortest side chain, the *C*_free,medium_ was found to be equal to the *C*_nom_ in both bioassays. In contrast, the *C*_free,medium_ deviated from the *C*_nom_ by up to 4 orders of magnitude for IM1-16 Cl and N11-16-1Ph Cl, which have the longest side chains, in both assays (Table S11). When freely dissolved concentrations (IC_10,free_) are used as a dose metric, the cytotoxicity increased linearly with the length of the alkyl chain within a homologous series, while the cytotoxicity based on IC_10,nom,corr_ levels off for IL cations having alkyl chains containing more than 12 carbon atoms (Figure 4). Such a leveling off was observed for ILs^48,75^ and cationic surfactants^56^ when (cyto)toxicity was reported on the nominal concentration-basis for compounds substituted with longer alkyl side chains (*C* > 12). This phenomenon, the so-called “side chain cut-off”, has been attributed to factors such as reduced (bio)availability of IL molecules above the CMC level^43^ or the less detrimental impacts on membrane functions due to the structural similarity between long alkyl chain and membrane lipids.^76^ In this study, we demonstrate that cytotoxicity is not reduced for long-chain homologues but keeps increasing if IC_10,free_ is considered instead of IC_10,nom_. For instance, the IC_10,nom_ of IM1-14 Cl and IM1-16 Cl in the AREc32 assay were very similar (Figure 4), but the IC_10,free_ of IM1-16 Cl was 60 times lower than the IC_10,free_ of IM1-14 Cl, indicating a significantly higher toxicity of the former compound. This highlights that a similar IC_10,nom_ does not necessarily represent an equivalent level of effects of the IL cation with high affinity for bioassay medium constituents and that the *C*_free,medium_ serves as a better dose metric. A similar phenomenon is expected to occur when other phases influencing the *C*_free,medium_ are present in the test medium.

### 3.7 Cell Membrane Concentrations of IL Cations

Critical membrane burden (CMB) leading to baseline toxicity is known to be constant and independent of chemical or organism type.^38,39^ Thus, the CMB can be used as an anchor to distinguish between baseline and excess toxicants.^38^ The IC_10,membrane_ values of the IL cations were calculated from IC_10,free_ by eq 21 and compared with the known CMB (26 mmol/L_lip_).^40^

**Figure 5:**
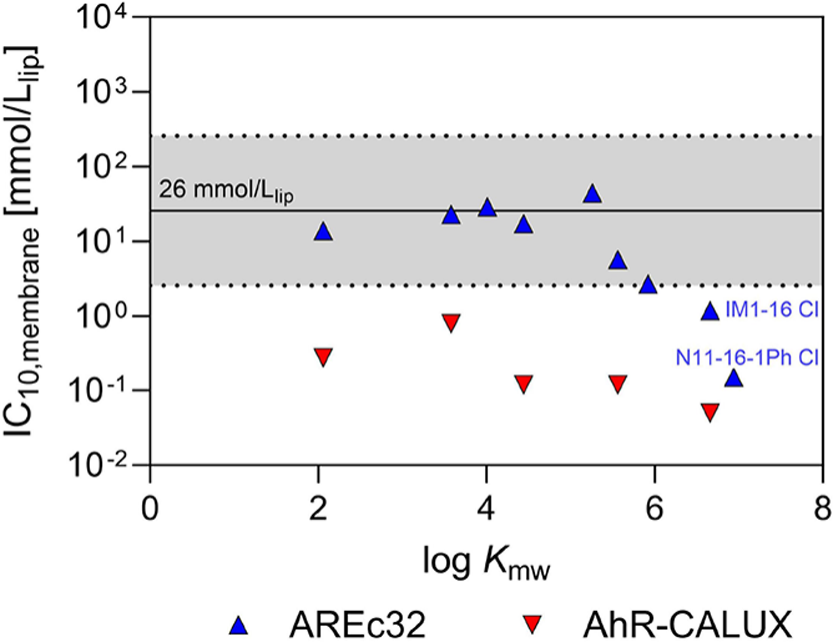
Cell membrane concentration (IC_10,membrane_) calculated from the experimental freely dissolved concentration (IC_10,free_) and the membrane lipid–water partition coefficient (log *K*_mw_) using eq 21 in the AREc32 (blue triangles) and AhR-CALUX assays (red inverted triangles) plotted against log *K*_mw_. The dotted lines represent a factor of 10 in each direction from the critical membrane burden (CMB) of 26 mmol/L_lip_ (denoted by the solid line).

Figure 5 shows the IC_10,membrane_ values for nine IL cations in the AREc32 and five in the AhR-CALUX assays. The IC_10,membrane_ ranged from 0.15 to 44.5 mmol/L_lip_ with an average of 15.3 mmol/L_lip_ in the AREc32 assay while it ranged from 0.05 to 0.80 mmol/L_lip_ with a mean of 0.27 mmol/L_lip_ in the AhR-CALUX assay (Table S11). The two most hydrophobic IL cations (IM1-16 Cl and N11-16-1Ph Cl) in the AREc32 assay and all IM1-R Cl in the AhR-CALUX assay showed the IC_10,membrane_ to be 1 order of magnitude lower than the CMB (= 26 mmol/L_lip_), classifying them as excess toxicants. This finding aligns well with previous studies where those compounds showed much higher cytotoxicity than baseline toxicity.^42^ On the contrary, all IL cations with 10–14 carbons in the side chain in the AREc32 assay fell within the range of CMB (gray area), whereas they were previously identified as excess toxicants based on the CMB of 69 mmol/L_lip_.^42^

Considering that the CMB of 26 mmol/L_lip_ derived from the direct measurement of IC_10,free_ (eq 21) is more robust than the earlier CMB of 69 mmol/L_lip_ that was simply modeled from the nominal concentration,^40,64^ the results obtained in this study should be considered as more reliable. However, the CMB calculation (eq 21) relies on the assumption that chemical partitioning to the cell membrane can be described by *K*_mw_. The *K*_mw_ values used in this study were determined exclusively with neutral POPC, whereas actual cell membranes can comprise a diverse range of phospholipids with variable affinities for charged compounds. For example, the fraction of anionic phospholipids in rat tissue varied from 0.06 to 18% depending on the tissue origin.^77^ The electrostatic interactions between the negative charge of the anionic phospholipids and positively charged molecules may influence the affinity of organic cations to cell membranes containing large proportions of anionic lipids. Several studies have indicated that cationic compounds showed a higher binding affinity for negatively charged lipid bilayers than for 100% neutral POPC bilayers.^44,78,79^ If cell membranes contain lipids that reduce or enhance interactions with IL cations compared to what is expected from POPC, the use of *K*_mw_ derived solely from POPC may lead to the incorrect calculation of IC_10,membrane_ in eq 21. Indeed, the experimental *K*_cell/w_ of the IL cations were higher than the MBM prediction by more than an order of magnitude in the AREc32 cells and by up to two orders of magnitude in the AhR-CALUX cells. These findings imply that POPC and serum albumin are insufficient surrogates for cellular lipids and proteins. If the cellular lipids are the phase responsible for the observed deviation and the primary driver of enhanced partitioning into cells, then the IC_10,membrane_ calculated using POPC-derived *K*_mw_ in eq 21 may be underestimated.

## 4 Conclusions

Our study demonstrated that the partitioning behavior of (permanently charged) organic cations of ionic liquids in *in vitro* cell-based bioassays can be distinctly different from those of neutral or anionic chemicals. Their properties, such as affinity for various biological (e.g., serum albumin, phospholipids, or cells) or synthetic materials, such as plate plastics or micelle formation, influence the distribution of these compounds and, therefore, are needed to precisely describe the fate in bioassays and develop a toxicity prediction model.

Freely dissolved concentrations (*C*_free_) are better metrics for toxicological effects as they inherently account for compound losses, e.g., due to sorption to the test vessel or components of the assay medium and are therefore preferred over nominal concentrations (*C*_nom_), yet the latter are far more often reported. The mass balance model (MBM) could predict the *C*_free,medium_ of the IL cations in a bioassay medium. As literature cytotoxicity data of IL, have almost exclusively been reported in nominal concentrations, the MBM allows recalculation of *C*_nom_ into *C*_free,medium_, provided that reliable partition coefficients (*K*_albumin/w_, *K*_mw_, and *K*_cell/w_) and assay information (e.g., the composition of medium and cells) are available. In this study, significant differences between *C*_nom_ and *C*_free,medium_ started to occur for compounds with a log *K*_mw_ of approximately 4.5 (chain length ≥10 carbons) and reached up to 4 orders of magnitude for compounds with a log *K*_mw_ of 7 (chain length = 16 carbons). While cytotoxicity expressed in terms of C_free,medium_ continued to increase with increasing *K*_mw_ (showing no signs of leveling off), cytotoxicity expressed in terms of *C*_nominal_ leveled off (showing so-called “toxicity cut-off”) for more hydrophobic compounds (log*K*_mw_ > 4.5). Our results demonstrate that the reduction of the freely available fraction is responsible for the leveling off of cytotoxicity expressed in terms of nominal concentrations and that the toxicity cut-off is an artifact of this. Thus, reporting effects in nominal concentration falsifies toxicity evaluation for compounds with a high affinity for the assay medium. This has important implications for hazard assessment of compounds that experience high deviations between *C*_nom_ and *C*_free_, since such compounds appear to be less toxic than they actually are, with differences amounting to several orders of magnitude. Using such data, for example, the development of predictive models or the definition of toxicity thresholds, will be inherently flawed.

The affinity of the IL cations for AREc32 and AhR-CALUX cells (*K*_cell/w_) was higher than predicted by MBM based on the partition coefficients between membrane lipid and water (*K*_mw_) and serum albumin and water (*K*_albumin/w_). This implies that serum albumin and phosphatidylcholine may not be suitable surrogates for cell membranes when describing the affinity of the IL cations for cells. Moreover, discrepancies in cell affinity between cell lines may explain why the cytotoxicity of IL cations was generally higher in the AhR-CALUX assay than in the AREc32 assay. This finding may also account for large differences in the sensitivity of various cell lines to ILs often observed in other studies.^80,81^ The cell type-dependent affinity may hinder the direct comparison of effect data between different bioassays. It is, therefore, necessary to elucidate the origin of differing affinity for various cell lines to identify more suitable surrogate biomolecules (e.g., different lipids, proteins, or other biomolecules), which could satisfactorily describe the affinity of the IL cation for cells.

The IC_10,membrane_ calculated from the measured IC_10,free_ can be used to distinguish baseline toxicant from excess toxicant. The MOA classification of the IL cations based on the CMB of 26 mmol/L_lip_ in this study agreed with our previous study based on a CMB of 69 mmol/L_lip_^42^ while a few cations changed their MOA from excess toxicity to baseline toxicity. Considering the current CMB of 26 mmol/L_lip_ was directly derived from the experimental IC_10,free_ and required fewer assumptions than the earlier CMB of 69 mmol/L_lip_, the results obtained in this study should be considered more robust. However, it is uncertain if the *K*_mw_ of the IL cations measured using POPC bilayers is sufficient to describe the partitioning to cellular lipids since the MBM using POPC and serum albumin as surrogates for cell lipid and proteins failed to accurately predict the *K*_cell/w_ in both cell lines. However, it remains unclear which specific cellular phase is responsible for this deviation. Therefore, it is crucial to unravel the factors that drive the cell affinity of IL cations to refine the CMB approach in distinguishing baseline toxicants from excess toxicants.

## Data Availability

Data will be made available upon request.

## Supporting Information Available

Chemicals and bioassay information; details of determination of critical micelle concentration, plate binding, serum albumin binding, membrane lipid binding, and cell binding; details on *K*_mw_ prediction models; results of unbound fraction and *C*_free_ measurement and the comparison with modeled values; and concentration–response curves (PDF)

## References

[ref1] Greer A. J.; Jacquemin J.; Hardacre C. Industrial Applications of Ionic Liquids. Molecules 2020, 25, 5207 10.3390/molecules25215207. 33182328 PMC7664896

[ref2] Kalb R. S. Toward Industrialization of Ionic Liquids BT - Commercial Applications of Ionic Liquids; Shiflett M. B., Ed.; Springer International Publishing: Cham, 2020; pp 261–282.

[ref3] Chevron Corporation. 2021. https://www.chevron.com/newsroom/2021/q2/chevron-and-honeywell-announce-start-up-of-isoalky-ionic-liquids-alkylation-unit (accessed April 18, 2024).

[ref4] Well-Resources. Ionikylation. https://www.wellresources.ca/ionikylation (accessed April 18, 2024).

[ref5] Haverhals L. M.; Reichert W. M.; De Long H. C.; Trulove P. C. Natural Fiber Welding. Macromol. Mater. Eng. 2010, 295 (5), 425–430. 10.1002/mame.201000005.

[ref6] Ioniqa. Ioniqa takes first 10 kiloton PET upcycling factory into operation. https://ioniqa.com/ioniqa-takes-first-10-kiloton-pet-upcycling-factory-into-operation/ (accessed April 18, 2024).

[ref7] Solvionic. https://solvionic.com/en/ (accessed June 24, 2024).

[ref8] Innovations E. Electrolyte for supercapacitors. https://e-lyte.de/products/electrolytes-for-batteries/electrolyte-for-supercapacitors/ (accessed April 18, 2024).

[ref9] IoLiTec. Ionic Liquids as Electroytes: Dye-sensitised solar cells. https://iolitec.de/en/technology/energy-cleantech/dye-sensitised-solar-cells (accessed April 18, 2024).

[ref10] Flector Patch (diclofenac epolamine). https://flector.com/ (accessed Nov 18, 2024).

[ref11] Grand view research. https://www.grandviewresearch.com/industry-analysis/ionic-liquids-market (accessed April 18, 2024).

[ref12] Pati S. G.; Arnold W. A. Comprehensive Screening of Quaternary Ammonium Surfactants and Ionic Liquids in Wastewater Effluents and Lake Sediments. Environ. Sci. Process. Impacts 2020, 22 (2), 430–441. 10.1039/C9EM00554D. 32003378

[ref13] Probert P. M.; Leitch A. C.; Dunn M. P.; Meyer S. K.; Palmer J. M.; Abdelghany T. M.; Lakey A. F.; Cooke M. P.; Talbot H.; Wills C.; McFarlane W.; Blake L. I.; Rosenmai A. K.; Oskarsson A.; Figueiredo R.; Wilson C.; Kass G. E.; Jones D. E.; Blain P. G.; Wright M. C. Identification of a Xenobiotic as a Potential Environmental Trigger in Primary Biliary Cholangitis. J. Hepatol. 2018, 69 (5), 1123–1135. 10.1016/j.jhep.2018.06.027. 30006067 PMC6192827

[ref14] Neuwald I.; Muschket M.; Zahn D.; Berger U.; Seiwert B.; Meier T.; Kuckelkorn J.; Strobel C.; Knepper T. P.; Reemtsma T. Filling the Knowledge Gap: A Suspect Screening Study for 1310 Potentially Persistent and Mobile Chemicals with SFC- and HILIC-HRMS in Two German River Systems. Water Res. 2021, 204, 117645 10.1016/j.watres.2021.117645. 34547688

[ref15] Leitch A. C.; Ibrahim I.; Abdelghany T. M.; Charlton A.; Roper C.; Vidler D.; Palmer J. M.; Wilson C.; Jones D. E.; Blain P. G.; Wright M. C. The Methylimidazolium Ionic Liquid M8OI Is Detectable in Human Sera and Is Subject to Biliary Excretion in Perfused Human Liver. Toxicology 2021, 459 (June), 152854 10.1016/j.tox.2021.152854. 34271081 PMC8366605

[ref16] Ventura S. P. M.; Gonçalves A. M. M.; Sintra T.; Pereira J. L.; Gonçalves F.; Coutinho J. A. P. Designing Ionic Liquids: The Chemical Structure Role in the Toxicity. Ecotoxicology 2013, 22 (1), 1–12. 10.1007/s10646-012-0997-x. 23010869

[ref17] Beil S.; Markiewicz M.; Pereira C. S.; Stepnowski P.; Th J.; Stolte S. Toward the Proactive Design of Sustainable Chemicals: Ionic Liquids as a Prime Example. Chem. Rev. 2020, 121, 13132–13173. 10.1021/acs.chemrev.0c01265. 34523909

[ref18] Cho C. W.; Pham T. P. T.; Zhao Y.; Stolte S.; Yun Y. S. Review of the Toxic Effects of Ionic Liquids. Sci. Total Environ. 2021, 786, 147309 10.1016/j.scitotenv.2021.147309. 33975102

[ref19] Krewski D.; Acosta D. Jr; Andersen M.; Anderson H.; Bailar J. C. III; Boekelheide K.; Brent R.; Charnley G.; Cheung V. G.; Green S. Jr; Kelsey K. T.; Kerkvliet N. I.; Li A. A.; McCray L.; Meyer O. Toxicity Testing in the 21st Centry: A Vision and a Strategy. Toxicol. Environ. Health B Crit. Rev. 2010, 13 (2–4), 51–138. 10.1080/10937404.2010.483176. PMC4410863 20574894

[ref20] Yoon M.; Blaauboer B. J.; Clewell H. J. Quantitative in Vitro to in Vivo Extrapolation (QIVIVE): An Essential Element for in Vitro-Based Risk Assessment. Toxicology 2015, 332, 1–3. 10.1016/j.tox.2015.02.002. 25680635

[ref21] Blaauboer B. J. Biokinetic Modeling and in Vitro-in Vivo Extrapolations. *J. Toxicol. Environ. Heal. - Part B*. Crit. Rev. 2010, 13 (2–4), 242–252. 10.1080/10937404.2010.483940. 20574900

[ref22] Henneberger L.; Huchthausen J.; Wojtysiak N.; Escher B. I. Quantitative in Vitro-to- In Vivo Extrapolation: Nominal versus Freely Dissolved Concentration. Chem. Res. Toxicol. 2021, 34 (4), 1175–1182. 10.1021/acs.chemrestox.1c00037. 33759508

[ref23] Groothuis F. A.; Heringa M. B.; Nicol B.; Hermens J. L. M.; Blaauboer B. J.; Kramer N. I. Dose Metric Considerations in in Vitro Assays to Improve Quantitative in Vitro-in Vivo Dose Extrapolations. Toxicology 2015, 332, 30–40. 10.1016/j.tox.2013.08.012. 23978460

[ref24] Proença S.; Escher B. I.; Fischer F. C.; Fisher C.; Grégoire S.; Hewitt N. J.; Nicol B.; Paini A.; Kramer N. I. Effective Exposure of Chemicals in in Vitro Cell Systems: A Review of Chemical Distribution Models. Toxicol. In Vitro 2021, 73, 105133 10.1016/j.tiv.2021.105133. 33662518

[ref25] Escher B. I.; Glauch L.; König M.; Mayer P.; Schlichting R. Baseline Toxicity and Volatility Cutoff in Reporter Gene Assays Used for High-Throughput Screening. Chem. Res. Toxicol. 2019, 32 (8), 1646–1655. 10.1021/acs.chemrestox.9b00182. 31313575

[ref26] Riedl J.; Altenburger R. Physicochemical Substance Properties as Indicators for Unreliable Exposure in Microplate-Based Bioassays. Chemosphere 2007, 67 (11), 2210–2220. 10.1016/j.chemosphere.2006.12.022. 17275879

[ref27] Bourez S.; Van den Daelen C.; Le Lay S.; Poupaert J.; Larondelle Y.; Thomé J. P.; Schneider Y. J.; Dugail I.; Debier C. The Dynamics of Accumulation of PCBs in Cultured Adipocytes Vary with the Cell Lipid Content and the Lipophilicity of the Congener. Toxicol. Lett. 2013, 216 (1), 40–46. 10.1016/j.toxlet.2012.09.027. 23164672

[ref28] Fischer F. C.; Cirpka O. A.; Goss K. U.; Henneberger L.; Escher B. I. Application of Experimental Polystyrene Partition Constants and Diffusion Coefficients to Predict the Sorption of Neutral Organic Chemicals to Multiwell Plates in in Vivo and in Vitro Bioassays. Environ. Sci. Technol. 2018, 52 (22), 13511–13522. 10.1021/acs.est.8b04246. 30298728

[ref29] Fischer F. C.; Abele C.; Henneberger L.; Klüver N.; König M.; Mühlenbrink M.; Schlichting R.; Escher B. I. Cellular Metabolism in High-Throughput in Vitro Reporter Gene Assays and Implications for the Quantitative in Vitro- In Vivo Extrapolation. Chem. Res. Toxicol. 2020, 33 (7), 1770–1779. 10.1021/acs.chemrestox.0c00037. 32227843

[ref30] Gülden M.; Seibert H. In Vitro-in Vivo Extrapolation: Estimation of Human Serum Concentrations of Chemicals Equivalent to Cytotoxic Concentrations in Vitro. Toxicology 2003, 189 (3), 211–222. 10.1016/S0300-483X(03)00146-X. 12832154

[ref31] Gülden M.; Mörchel S.; Seibert H. Factors Influencing Nominal Effective Concentrations of Chemical Compounds in Vitro: Cell Concentration. Toxicol. Vitr. 2001, 15 (3), 233–243. 10.1016/S0887-2333(01)00008-X. 11377096

[ref32] Armitage J. M.; Wania F.; Arnot J. A. Application of Mass Balance Models and the Chemical Activity Concept to Facilitate the Use of in Vitro Toxicity Data for Risk Assessment. Environ. Sci. Technol. 2014, 48 (16), 9770–9779. 10.1021/es501955g. 25014875

[ref33] Fischer F. C.; Henneberger L.; König M.; Bittermann K.; Linden L.; Goss K. U.; Escher B. I. Modeling Exposure in the Tox21 in Vitro Bioassays. Chem. Res. Toxicol. 2017, 30 (5), 1197–1208. 10.1021/acs.chemrestox.7b00023. 28316234

[ref34] Henneberger L.; Mühlenbrink M.; König M.; Schlichting R.; Fischer F. C.; Escher B. I. Quantification of Freely Dissolved Effect Concentrations in in Vitro Cell-Based Bioassays. Arch. Toxicol. 2019, 93 (8), 2295–2305. 10.1007/s00204-019-02498-3. 31230094

[ref35] Qin W.; Henneberger L.; Huchthausen J.; König M.; Escher B. I. Role of Bioavailability and Protein Binding of Four Anionic Perfluoroalkyl Substances in Cell-Based Bioassays for Quantitative in Vitro to in Vivo Extrapolations. Environ. Int. 2023, 173, 107857 10.1016/j.envint.2023.107857. 36881956

[ref36] Łuczak J.; Hupka J.; Thöming J.; Jungnickel C. Self-Organization of Imidazolium Ionic Liquids in Aqueous Solution. Colloids Surfaces A Physicochem. Eng. Asp. 2008, 329 (3), 125–133. 10.1016/j.colsurfa.2008.07.012.

[ref37] Kalam S.; Abu-Khamsin S. A.; Kamal M. S.; Patil S. Surfactant Adsorption Isotherms: A Review. ACS Omega 2021, 6 (48), 32342–32348. 10.1021/acsomega.1c04661. 34901587 PMC8655760

[ref38] Escher B. I.; Hermens J. L. M. Modes of Action in Ecotoxicology: Their Role in Body Burdens, Species Sensitivity, QSARs, and Mixture Effects. Environ. Sci. Technol. 2002, 36 (20), 4201–4217. 10.1021/es015848h. 12387389

[ref39] Escher B. I.; Ashauer R.; Dyer S.; Hermens J. L. M.; Lee J. H.; Leslie H. A.; Mayer P.; Meador J. P.; Warnekk M. S. J. Crucial Role of Mechanisms and Modes of Toxic Action for Understanding Tissue Residue Toxicity and Internal Effect Concentrations of Organic Chemicals. Integr. Environ. Assess. Manag. 2011, 7 (1), 28–49. 10.1002/ieam.100. 21184568

[ref40] Huchthausen J.; Braasch J.; Escher B. I.; Maria K.; Henneberger L. Effects of Chemicals in Reporter Gene Bioassays with Different Metabolic Activities Compared to Baseline Toxicity. Chem. Res. Toxicol. 2024, 37, 744–756. 10.1021/acs.chemrestox.4c00017. 38652132 PMC11110108

[ref41] Jungnickel C.; Łuczak J.; Ranke J.; Fernández J. F.; Müller A.; Thöming J. Micelle Formation of Imidazolium Ionic Liquids in Aqueous Solution. Colloids Surfaces A Physicochem. Eng. Asp. 2008, 316 (1–3), 278–284. 10.1016/j.colsurfa.2007.09.020.

[ref42] Bae E.; Beil S.; König M.; Stolte S.; Escher B. I.; Markiewicz M. The Mode of Toxic Action of Ionic Liquids: Narrowing down Possibilities Using High-Throughput in Vitro Cell-Based Bioassays. Environ. Int. 2024, 193, 109089 10.1016/j.envint.2024.109089. 39500119

[ref43] Dołżonek J.; Cho C. W.; Stepnowski P.; Markiewicz M.; Thöming J.; Stolte S. Membrane Partitioning of Ionic Liquid Cations, Anions and Ion Pairs–Estimating the Bioconcentration Potential of Organic Ions. Environ. Pollut. 2017, 228, 378–389. 10.1016/j.envpol.2017.04.079. 28554027

[ref44] Loidl-Stahlhofen A.; Hartmann T.; Schöttner M.; Rhöring C.; Brodowsky H.; Schmitt J.; Keldenich J. Multilamellar Liposomes and Solid-Supported Lipid Membranes (TRANSIL): Screening of Lipid-Water Partitioning toward a High-Throughput Scale. Pharm. Res. 2001, 18 (12), 1782–1788. 10.1023/A:1013343117979. 11785701

[ref45] Sovicell. TRANSIL ASSAY KITS Frequently Asked Questions (FAQs).

[ref46] Stolte S.; Matzke M.; Arning J.; Böschen A.; Pitner W. R.; Welz-Biermann U.; Jastorff B.; Ranke J. Effects of Different Head Groups and Functionalised Side Chains on the Aquatic Toxicity of Ionic Liquids. Green Chem. 2007, 9 (11), 1170–1179. 10.1039/b711119c.

[ref47] Loidl-stahlhofen B. A.; Schmitt J.; Nöller J.; Hartmann T.; Brodowsky H.; Schmitt W.; Keldenich J. Solid-Supported Biomolecules on Modified Silica SurfacesĐA Tool for Fast Physicochemical Characterization and High-Throughput Screening**. Adv. Mater. 2001, 13, 1829–1834. 10.1002/1521-4095(200112)13:233.0.CO;2-3.

[ref48] Ranke J.; Müller A.; Bottin-Weber U.; Stock F.; Stolte S.; Arning J.; Störmann R.; Jastorff B. Lipophilicity Parameters for Ionic Liquid Cations and Their Correlation to in Vitro Cytotoxicity. Ecotoxicol. Environ. Saf. 2007, 67 (3), 430–438. 10.1016/j.ecoenv.2006.08.008. 17034854

[ref49] Endo S.; Goss K. U. Serum Albumin Binding of Structurally Diverse Neutral Organic Compounds: Data and Models. Chem. Res. Toxicol. 2011, 24 (12), 2293–2301. 10.1021/tx200431b. 22070391

[ref50] Henneberger L.; Mühlenbrink M.; Fischer F. C.; Escher B. I. C18-Coated Solid-Phase Microextraction Fibers for the Quantification of Partitioning of Organic Acids to Proteins, Lipids, and Cells. Chem. Res. Toxicol. 2019, 32 (1), 168–178. 10.1021/acs.chemrestox.8b00249. 30585484

[ref51] Qin W.; Henneberger L.; Glüge J.; König M.; Escher B. I. Baseline Toxicity Model to Identify the Specific and Nonspecific Effects of Per- and Polyfluoroalkyl Substances in Cell-Based Bioassays. Environ. Sci. Technol. 2024, 58, 5727 10.1021/acs.est.3c09950. 38394616 PMC10993398

[ref52] Escher B. I.; Neale P. A.; Villeneuve D. L. The Advantages of Linear Concentration–Response Curves for in Vitro Bioassays with Environmental Samples. Environ. Toxicol. Chem. 2018, 37 (9), 2273–2280. 10.1002/etc.4178. 29846006 PMC6150494

[ref53] Lasch J. Interaction of Detergents with Lipid Vesicles. BBA - Rev. Biomembr. 1995, 1241 (2), 269–292. 10.1016/0304-4157(95)00010-O. 7640298

[ref54] Kowalska D.; Maculewicz J.; Stepnowski P.; Dołżonek J. Interaction of Pharmaceutical Metabolites with Blood Proteins and Membrane Lipids in the View of Bioconcentration: A Preliminary Study Based on in Vitro Assessment. Sci. Total Environ. 2021, 783, 146987 10.1016/j.scitotenv.2021.146987. 33866166

[ref55] Klevens H. B. Structure and Aggregation in Dilate Solution of Surface Active Agents. J. Am. Oil Chem. Soc. 1953, 30 (2), 74–80. 10.1007/BF02635002.

[ref56] Groothuis F. A.; Timmer N.; Opsahl E.; Nicol B.; Droge S. T. J.; Blaauboer B. J.; Kramer N. I. Influence of in Vitro Assay Setup on the Apparent Cytotoxic Potency of Benzalkonium Chlorides. Chem. Res. Toxicol. 2019, 32 (6), 1103–1114. 10.1021/acs.chemrestox.8b00412. 31012305 PMC6584903

[ref57] Somasundaran P.; Fuerstenau D. W. Mechanisms of Alkyl Sulfonate Adsorption at the Alumina-Water Interface. J. Phys. Chem. 1966, 70 (1), 90–96. 10.1021/j100873a014.

[ref58] Markiewicz M.; Mrozik W.; Rezwan K.; Thöming J.; Hupka J.; Jungnickel C. Changes in Zeta Potential of Imidazolium Ionic Liquids Modified Minerals - Implications for Determining Mechanism of Adsorption. Chemosphere 2013, 90 (2), 706–712. 10.1016/j.chemosphere.2012.09.053. 23079161

[ref59] Timmer N.; Droge S. T. J. Sorption of Cationic Surfactants to Artificial Cell Membranes: Comparing Phospholipid Bilayers with Monolayer Coatings and Molecular Simulations. Environ. Sci. Technol. 2017, 51 (5), 2890–2898. 10.1021/acs.est.6b05662. 28187261 PMC5343551

[ref60] Droge S. T. J. Membrane-Water Partition Coefficients to Aid Risk Assessment of Perfluoroalkyl Anions and Alkyl Sulfates. Environ. Sci. Technol. 2019, 53 (2), 760–770. 10.1021/acs.est.8b05052. 30572703

[ref61] Yan H.; Wu J.; Dai G.; Zhong A.; Chen H.; Yang J.; Han D. Interaction Mechanisms of Ionic Liquids [C Nmim]Br (N = 4, 6, 8, 10) with Bovine Serum Albumin. J. Lumin. 2012, 132 (3), 622–628. 10.1016/j.jlumin.2011.10.026.

[ref62] Huang R.; Zhang S.; Pan L.; Li J.; Liu F.; Liu H. Spectroscopic Studies on the Interactions between Imidazolium Chloride Ionic Liquids and Bovine Serum Albumin. Spectrochim. Acta - Part A Mol. Biomol. Spectrosc. 2013, 104, 377–382. 10.1016/j.saa.2012.11.087. 23274265

[ref63] Zhou T.; Ao M.; Xu G.; Liu T.; Zhang J. Interactions of Bovine Serum Albumin with Cationic Imidazolium and Quaternary Ammonium Gemini Surfactants: Effects of Surfactant Architecture. J. Colloid Interface Sci. 2013, 389 (1), 175–181. 10.1016/j.jcis.2012.08.067. 23044272

[ref64] Lee J.; Braun G.; Henneberger L.; König M.; Schlichting R.; Scholz S.; Escher B. I. Critical Membrane Concentration and Mass-Balance Model to Identify Baseline Cytotoxicity of Hydrophobic and Ionizable Organic Chemicals in Mammalian Cell Lines. Chem. Res. Toxicol. 2021, 34, 2100 10.1021/acs.chemrestox.1c00182. 34357765

[ref65] Henneberger L.; Goss K. U.; Endo S. Equilibrium Sorption of Structurally Diverse Organic Ions to Bovine Serum Albumin. Environ. Sci. Technol. 2016, 50 (10), 5119–5126. 10.1021/acs.est.5b06176. 27098963

[ref66] Zsila F. Subdomain IB Is the Third Major Drug Binding Region of Human Serum Albumin: Toward the Three-Sites Model. Mol. Pharmaceutics 2013, 10 (5), 1668–1682. 10.1021/mp400027q. 23473402

[ref67] Fischer F. C.; Abele C.; Droge S. T. J.; Henneberger L.; König M.; Schlichting R.; Scholz S.; Escher B. I. Cellular Uptake Kinetics of Neutral and Charged Chemicals in in Vitro Assays Measured by Fluorescence Microscopy. Chem. Res. Toxicol. 2018, 31 (8), 646–657. 10.1021/acs.chemrestox.8b00019. 29939727

[ref68] Kontro I.; Svedström K.; Duša F.; Ahvenainen P.; Ruokonen S. K.; Witos J.; Wiedmer S. K. Effects of Phosphonium-Based Ionic Liquids on Phospholipid Membranes Studied by Small-Angle X-Ray Scattering. Chem. Phys. Lipids 2016, 201, 59–66. 10.1016/j.chemphyslip.2016.11.003. 27836694

[ref69] Mendonca C. M. N.; Balogh D. T.; Barbosa S. C.; Sintra T. E.; Ventura S. P. M.; Martins L. F. G.; Morgado P.; Filipe E. J. M.; Coutinho J. A. P.; Oliveira O. N.; Barros-Timmons A. Understanding the Interactions of Imidazolium-Based Ionic Liquids with Cell Membrane Models. Phys. Chem. Chem. Phys. 2018, 20 (47), 29764–29777. 10.1039/C8CP05035J. 30462106

[ref70] Krämer S. D.; Jakits-Deiser C.; Wunderli-Allenspach H. Free Fatty Acids Cause PH Dependent Changes in Drug-Lipid Membrane Interactions around Pyisiological PH. Pharm. Res. 1997, 14, 827–832. 10.1023/a:1012175111401. 9210205

[ref71] Krämer S. D.; Braun A.; Jakits-Deiser C.; Wunderli-Allenspach H. Towards the Predictability of Drug-Lipid Membrane Interactions: The PH-Dependent Affinity of Propranolol to Phosphatidylinositol Containing Liposomes. Pharm. Res. 1998, 15 (5), 739–744. 10.1023/A:1011923103938. 9619783

[ref72] Goss K. U.; Bittermann K.; Henneberger L.; Linden L. Equilibrium Biopartitioning of Organic Anions–A Case Study for Humans and Fish. Chemosphere 2018, 199, 174–181. 10.1016/j.chemosphere.2018.02.026. 29438944

[ref73] Henneberger L.; Goss K. U.; Endo S. Partitioning of Organic Ions to Muscle Protein: Experimental Data, Modeling, and Implications for in Vivo Distribution of Organic Ions. Environ. Sci. Technol. 2016, 50 (13), 7029–7036. 10.1021/acs.est.6b01417. 27265315

[ref74] Bae C.; Chung G.; Chung S. J. Prediction of the Time to Reach Equilibrium for Improved Estimation of the Unbound Fraction of Compounds in Equilibrium Dialysis Using Kinetic Modeling. J. Pharm. Sci. 2023, 112 (11), 2901–2909. 10.1016/j.xphs.2023.06.015. 37392902

[ref75] Łuczak J.; Jungnickel C.; Łącka I.; Stolte S.; Hupka J. Antimicrobial and Surface Activity of 1-Alkyl-3-Methylimidazolium Derivatives. Green Chem. 2010, 12 (4), 593–601. 10.1039/b921805j.

[ref76] Drücker P.; Rühling A.; Grill D.; Wang D.; Draeger A.; Gerke V.; Glorius F.; Galla H. J. Imidazolium Salts Mimicking the Structure of Natural Lipids Exploit Remarkable Properties Forming Lamellar Phases and Giant Vesicles. Langmuir 2017, 33 (6), 1333–1342. 10.1021/acs.langmuir.6b03182. 27935708

[ref77] Rodgers T.; Leahy D.; Rowland M. Tissue Distribution of Basic Drugs: Accounting for Enantiomeric, Compound and Regional Differences amongst β-Blocking Drugs in Rat. J. Pharm. Sci. 2005, 94 (6), 1237–1248. 10.1002/jps.20323. 15858851

[ref78] Krämer S. D.; Jakits-Deiser C.; Wunderli-Allenspach H. Free Fatty Acids Cause PH Dependent Chnages in Lipid-Membrane Interactions. Pharm. Res. 1997, 14 (6), 827–832. 10.1023/A:1012175111401. 9210205

[ref79] Krämer S. D.; Braun A.; Jakits-Deiser C.; Wunderli-Allenspach H. Towards the Predictability of Drug-Lipid Membrane Interactions: The PH- Dependent Affinity of Propranolol to Phosphatidylinositol Containing Liposomes. Pharm. Res. 1998, 15, 739–744. 10.1023/A:1011923103938. 9619783

[ref80] Dzhemileva L. U.; D’Yakonov V. A.; Seitkalieva M. M.; Kulikovskaya N. S.; Egorova K. S.; Ananikov V. P. A Large-Scale Study of Ionic Liquids Employed in Chemistry and Energy Research to Reveal Cytotoxicity Mechanisms and to Develop a Safe Design Guide. Green Chem. 2021, 23 (17), 6414–6430. 10.1039/D1GC01520F.

[ref81] Kumar V.; Malhotra S. V. Synthesis of Nucleoside-Based Antiviral Drugs in Ionic Liquids. Bioorg. Med. Chem. Lett. 2008, 18 (20), 5640–5642. 10.1016/j.bmcl.2008.08.090. 18796352

