# Supplementary material for: Assessing Modes of Toxic Action of Organic Cations in In Vitro Cell-Based Bioassays: the Critical Role of Partitioning to Cells and Medium Components
Source: Chem Res Toxicol. 2025 Mar 4;38(3):488–502. doi: 10.1021/acs.chemrestox.4c00527 (PMC11921022; doi:10.1021/acs.chemrestox.4c00527)
Supplement: Supplementary file 1 — tx4c00527_si_001.pdf [file tx4c00527_si_001.pdf]

Supporting information for "Assessing modes of toxic action of organic cations in *in vitro* cell-based bioassays: the critical role of partitioning to cells and medium components"

Eunhye Bae[†], Stephan Beil[†], Maria König[‡], Stefan Stolte[†], Beate I. Escher[‡, §], and Marta Markiewicz[†*]

[†]Institute of Water Chemistry, Dresden University of Technology, D-01062 Dresden, Germany

[‡]Department of Cell Toxicology, Helmholtz Centre for Environmental Research-UFZ, D-04318, Leipzig, Germany

[§]Environmental Toxicology, Department of Geosciences, Eberhard Karls University Tübingen, D-72076 Tübingen, Germany

Corresponding author

*Address: Institute of Water Chemistry, Dresden University of Technology, D-01062 Dresden, Germany, Phone +49 463-33872, E-mail: marta.markiewicz@tu-dresden.de

Table of Content

Table S1. List of compounds used in this study for LC/MS/MS analysis 4

Table S2. Molecular formular, molecular weight and structure of ionic liquids tested.. 5

Table S3. LC-MS/MS parameters.. 7

Figure S1. Experimental workflow of cell-water partition coefficient ($K_{cell/w}$) measurement 8

Table S4. Volume fractions of protein, lipids, and water in medium used in AREc32 and AhR-CALUX assay.. 9

Table S5. Volume fractions of protein, lipids, and water in cells used in AREc32 and AhR-CALUX bioassays.. 9

Table S6. Critical micelle concentration determined by the Nile red 9

Figure S2. Plots of fluorescence intensity of the Nile red against ionic liquids concentration... 10

Figure S3. Sorption isotherms to a 96-well plate in the serum-free medium 10

Table S7. List of membrane lipid-water partition constants (K_{mw}) and albumin-water partition constants ($K_{albumin/w}$) of cations of ionic lilquids used in this study 11

Figure S4. Sorption isotherms of six cations of ionic liquids for membrane lipid-water partition constants (K_{mw}) using the standard TRANSIL Intestinal Absorption kit 12

Figure S5. The experimental membrane lipid-water partition constants (log K_{mw}) plotted against carbon number in the alkyl side chain of 1-methyl-3-alkylimidazolium chlorides 13

Figure S6. A sorption isotherm of IM1-12 Cl in the solid-supported lipid membrane assay by differentiating phospholipid/sorbed compound molar ratio and corresponding membrane-water partition coefficient 13

Text S1. Polyparameter Linear free energy relationship (pp-LFERs) models to predict octanol-water partitioning (K_{ow}), chromatographic capacity factor (k_0) and membrane lipid-water partition coefficients (K_{mw}) and the calculation of K_{mw} by COSMOmic. 14

Table S8. Polyparameter Linear free energy relationship (pp-LFER) solute descriptors of single ions of ionic liquids.. 15

Table S9. Octanol-water partition coefficients (K_{ow}), chromatographic capacity factor (k_0) and membrane lipid-water partition coefficients (K_{mw}) predicted or collected from literature... 16

Text S2. Comparison of prediction approaches for membrane lipid-water partitioning (log K_{mw}) of cations of ionic liquids 17

Figure S7. Comparison of accuracy between four prediction models for membrane lipid-water partition constants (log K_{mw}) of cations of ionic liquids 19

Figure S8. Sorption isotherms of five cations of ionic liquids for albumin-water partition constants ($K_{albumin/w}$) using the standard TRANSILXL HSA Binding Kit 20

Figure S9. Sorption isotherms of eight cations of ionic liquids for albumin-water partition constants ($K_{albumin/w}$) using the TRANSILXL HSA Binding Kit for low binders 21

Figure S10. The experimental albumin-water partition constants ($K_{albumin/w}$) plotted against the number of carbon atoms in the side chain 22

Figure S11. The cell-water partition coefficients ($K_{cell/w}$) measured in AREc32 cells plotted against with the nominal concentration that are corrected with plate binding ($C_{nom,corr}$). 23

Figure S12. The cell-water partition coefficients ($K_{cell/w}$) measured in AhR-CALUX cells plotted against with the nominal concentration that are corrected with plate binding ($C_{nom,corr}$)... 24

Figure S13. Linear relationships of membrane lipid-water (K_{mw}) and cell-water ($K_{cell/w}$) partition coefficients for AREc32 and AhR-CALUX cells 25

Figure S14. Equilibrium kinetics of N11-12-1Ph Cl and IM1-16 Cl in the rapid equilibrium dialysis (RED) system 26

Figure S15. The unbound fraction in medium (f_u %) determined by the rapid equilibrium dialysis (RED) plotted as a function of nominal concentration ($C_{nom,RED}$ μmol/L) 26

Table S10. The unbound fractions inmedium (f_u %) and recoveries of test chemicals in bioassay medium determined by the rapid equilibrium dialysis (RED) and modeled f_u (%) 27

Table S11. Inhibitory concentrations at 10% cytotoxicity based on nominal concentrations ($IC_{10,nom}$), nominal concentration that are corrected with plate binding ($IC_{10,nom,corr}$) freely dissolved cytotoxic concentrations ($IC_{10,free}$), cell membrane concentrations at $IC_{10,nom,corr}$ ($IC_{10,membrane}$) 28

Figure S16. Concentration-response curves of test compounds (based on nominal concentration corrected with plate binding, $C_{nom,corr}$) in AREc32 and AhR-CALUX assays. 29

Figure S17. Freely dissolved concentration in the medium ($C_{free,medium}$) of cations of ionic liquids of after 24 h exposure.. 32

Figure S18. Evaluation of contribution of cell binding to the freely dissolved concentration causing 10% cytotoxicity ($IC_{10,free}$) prediction in AREc32 and AhR-CALUX assay... 33

References... 34

Table S1. List of compounds tested in this study and internal standards used for LC/MS/MS analysis.

CAS-NO.	Compound name	Abbreviation	Purity	Supplier
65039-09-0	1-Methyl-3-ethylimidazolium chloride	IM1-2 Cl	>95%	Merck KGaA
79917-90-1	1-Methyl-3-buthylimidazolium chloride	IM1-4 Cl	99%	IOLITEC
171058-17-6	1-Methyl-3-hexylimidazolium chloride	IM1-6 Cl	>97%	Merck KGaA
64697-40-1	1-Methyl-3-octylimidazolium chloride	IM1-8 Cl	>98%	Merck KGaA
171058-18-7	1-Methyl-3-deylimidazolium chloride	IM1-10 Cl	>98%	Merck KGaA
114569-84-5	1-Methyl-3-dodecyllimidazolium chloride	IM1-12 Cl	>98%	IOLITEC
171058-21-2	1-Methyl-3-tetradecylimidazolium chloride	IM1-14 Cl	>98%	IOLITEC
61546-01-8	1-Methyl-3-hexadecylimidazolium chloride	IM1-16 Cl	>98%	Merck KGaA
N/A	1-Methyl-3-(2-phenylethyl)-3H-imidazolium chloride	IM1-2Ph Cl	N/A	ITUS, University in Jena
965-32-2	Benzyldimethyldecylammonium chloride	N11-10-1Ph Cl	>99%	Fluka Chemie GmbH
139-07-1	Benzyldimethyldodecylammonium chloride	N11-12-1Ph Cl	>99%	Fluka Chemie GmbH
139-08-2	Benzyldimethyltetradecylammonium chloride	N11-14-1Ph Cl	>99%	Fluka Chemie GmbH
122-18-9	Benzyldimethylhexadecylammonium chloride	N11-16-1Ph Cl	>99%	Fluka Chemie GmbH
1643-19-2	Tetrabutylammonium bromide	N4444 Br	>99%	Merck KGaA
3115-68-2	Tetrabutylphosphonium bromide	P4444 Br	98%	Merck KGaA
1124-64-7	1-Butylpyridinium chloride	Py4 Cl	98%	Merck KGaA
6220-15-1	1-Hexylpyridinium chloride	Py6 Cl	97%	Merck KGaA
4086-73-1	1-Octylpyridinium chloride	Py8 Cl	99%	IOLITEC
125652-55-3	1-Methyl-3-butylpyridinium chloride	Py4-3Me Cl	98%	Merck KGaA
N/A	1-Methyl-3-hecylpyridinium chloride	Py6-3Me Cl	N/A	Merck KGaA
N/A	1-Methyl-3-octylpyridinium chloride	Py8-3Me Cl	N/A	Merck KGaA
N/A	1-Methyl-4-octylpyridinium chloride	Py8-4Me Cl	N/A	Merck KGaA
N/A	4-(Dimethylamino)-1-ethylpyridinium bromide	Py2-4NMe2 Br	N/A	Merck KGaA
N/A	4-(Dimethylamino)-1-ethylpyridinium bromide	Py4-4NMe2 Cl	N/A	Merck KGaA
N/A	4-(Dimethylamino)-1-hexylpyridinium bromide	Py6-4NMe2 Cl	N/A	Merck KGaA
94280-72-5	1-Methyl-1-butylpiperidinium bromide	Pip1-4 Br	97%	Merck KGaA
93457-69-3	1-Methyl-1-butylpyrrolidinium bromide	Pyr1-4 Br	99%	Merck KGaA
N/A	1-Methyl-1-hexylpyrrolidinium chloride	Pyr1-6 Cl	N/A	Merck KGaA
160203-52-1	1-methyl-3-ethylimidazolium chloride-d11	D11-IM1-2 Cl	>99%	Santa Cruz Biotechnology,Inc.
N/A	D6-1-methyl-3-octylimidazolium bromide	D6-IM1-8 Cl	>99%	HPC Standards GmbH
N/A	D6-1-methyl-3-hexadecylimidazolium bromide	D6-IM1-16 Cl	>99%	HPC Standards GmbH
N/A	D5-octylpyridinium bromide	D5-Py8 Br	99%	HPC Standards GmbH
N/A	Tetra-n-butyl-d36-ammonium bromide	D36-N4444 Br	>98%	LGC standards
N/A	Benzyl-2,3,4,5,6,-d5-dimethyl-n-dodecylammonium chloride	D5-N11-12-1Ph Cl	>98%	LGC standards
1219178-72-9	Benzyldimethyltetradecylammonium-d7 chloride	D7-N11-14-1Ph Cl	>96%	LGC standards

*N/A indicates the customized chemicals without provided purity information

Table S2. Molecular formular, molecular weight and structure of compounds tested for partition coefficient determination and bioassays

Abbreviation	Molecular formula	M.W. [g/mol]	Structure	Abbreviation	Molecular formula	M.W. [g/mol]	Structure
IM1-2 Cl	C6H11ClN2	146.2	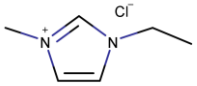	P4444 Br	C16H36PBr	339.3	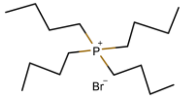
IM1-4 Cl	C8H15ClN2	174.7	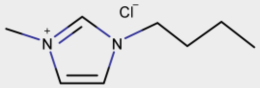	Py4 Cl	C9H14ClN	171.7	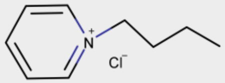
IM1-6 Cl	C10H19ClN2	202.7	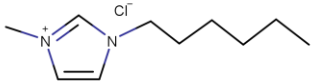	Py6 Cl	C11H18ClN	199.7	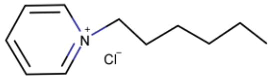
IM1-8 Cl	C12H23ClN2	230.8	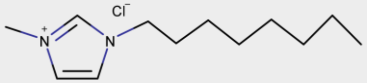	Py8 Cl	C13H22ClN	227.8	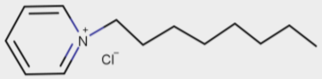
IM1-10 Cl	C14H27ClN2	258.8	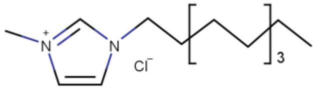	Py4-3Me Cl	C10H16ClN	185.7	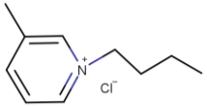
IM1-12 Cl	C16H31ClN2	286.9	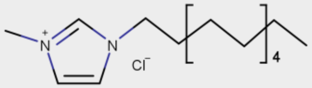	Py6-3Me Cl	C12H20ClN	213.8	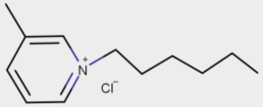
IM1-14 Cl	C18H35ClN2	314.9	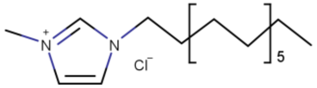	Py8-3Me Cl	C14H24ClN	241.8	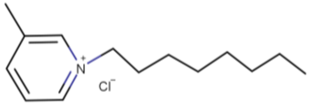

IM1-16 Cl	C20H39ClN2	343.0	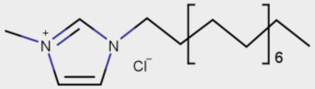	Py8-4Me Cl	C14H24ClN	241.8	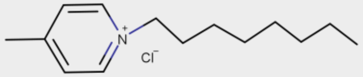
IM1-2Ph Cl	C12H15ClN2	222.7	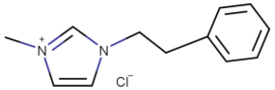	Py2-4NMe2 Br	C9H15BrN2	231.14	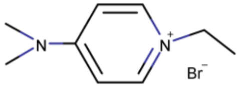
N11-10-1Ph Cl	C19H34ClN	311.9	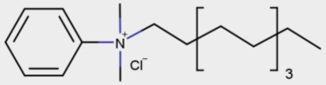	Py4-4NMe2 Cl	C11H19ClN2	214.7	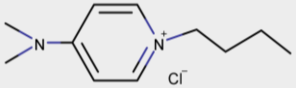
N11-12-1Ph Cl	C21H38ClN	340.0	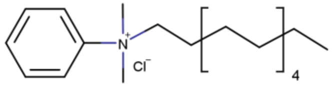	Py6-4NMe2 Cl	C13H23ClN2	242.8	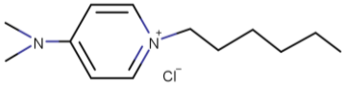
N11-14-1Ph Cl	C23H42ClN	368.0	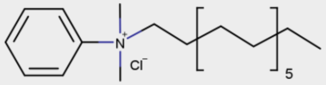	Pip1-4 Br	C10H22BrN	236.2	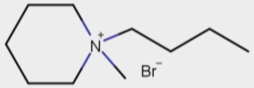
N11-16-1Ph Cl	C25H46ClN	396.1	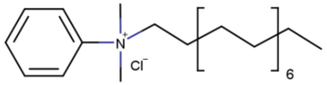	Pyr1-4 Br	C9H20BrN	222.2	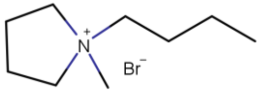
N4444 Br	C16H36BrN	322.4	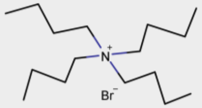	Pyr1-6 Cl	C11H24ClN	205.8	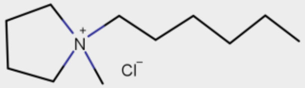

Table S3. LC/MS/MS parameters.

A linear solvent gradient composed of eluent A (1.25% formic acid, 5% acetonitrile, and 10 mM ammonium formate in water) and eluent B (1.25% formic acid, 5% water in acetonitrile) was used as described below. The same MS parameters (curtain gas (CUR) = 55 psi; collision gas (CAD) = 9 psi; collision cell entrance potential (EP) = 10 V) were set for all compounds. Compound-specific MS parameters are listed in Table S2 – declustering potential (DP); collision energy (CE); collision cell exit potential (CXP).

**Chemical**	**Elution method [%Eluent B]**	**RT [min]**	**LOD [ng/L]**	**LOQ [ng/L]**	**Q1 [m/z]**	**Q3 [m/z]**	**DP [V]**	**CE [V]**	**CXP [V]**
IM1-2 Cl	0-1.5 min: 95%<br>1.5-1.8 min: 20%<br>1.8-5 min: 20%<br>5-5.5 min: 95%<br>5.5-6.5 min: 95%	3.6	28.5	90.1	111.1	83.0	36	23	6
IM1-4 Cl		3.2	20.0	65.0	139.1	83.0	31	23	8
IM1-6 Cl		2.5	19.4	63.1	167.1	82.9	46	27	8
IM1-8 Cl		2.2	21.5	69.5	195.1	83.0	46	29	8
IM1-10 Cl		2.0	29.6	93.3	223.1	83.0	56	31	8
IM1-12 Cl		1.9	23.1	74.1	251.1	83.1	41	33	8
IM1-14 Cl		1.8	22.2	71.6	179.1	82.9	41	35	8
IM1-16 Cl		1.7	25.6	81.5	307.2	83.0	86	39	8
IM1-2Ph Cl		2.9	18.1	59.2	187.1	105.0	36	27	8
N11-10-1Ph Cl		1.8	2.3	7.4	276.2	90.9	1	49	8
N11-12-1Ph Cl		1.7	3.7	11.5	304.2	91.0	71	59	8
N11-14-1Ph Cl		1.7	3.0	9.4	332.2	91.0	31	65	8
N11-16-1Ph Cl		1.6	2.6	8.1	360.3	90.9	51	69	8
N4444 Br		1.8	3.8	11.5	242.2	142.1	56	33	8
P4444 Br		1.7	3.7	11.3	259.2	75.9	16	57	6
Py4 Cl	0-2 min: 95%<br>2-2.3 min: 20%<br>2.3-5.5 min: 20%<br>5.5-6 min: 95%<br>6-7.5 min: 95%	3.5	32.1	100.4	136.1	80.0	46	23	8
Py6 Cl		2.6	29.3	92.2	164.1	80.0	1	23	8
Py8 Cl		2.2	31.9	99.6	192.1	80.0	21	25	8
Py4-3Me Cl		3.0	32.3	100.1	150.0	94.0	26	25	8
Py6-3Me Cl		2.3	31.0	97.3	177.9	93.8	31	27	6
Py8-3(4)Me Cl		2.0	33.9	105.5	206.1	93.9	66	29	8
Py2-4NMe2 Br		3.6	36.9	113.8	151.1	123.0	41	29	8
Py4-4NMe2 Cl		2.6	37.0	113.9	179.1	123.1	31	31	6
Py6-4NMe2 Cl		2.1	29.4	92.5	207.1	123.0	16	33	10
Pip1-4 Br		3.2	23.5	75.3	156.1	86.0	21	31	8
Pyr1-4 Br		3.0	27.2	86.3	142.1	100.0	6	29	8

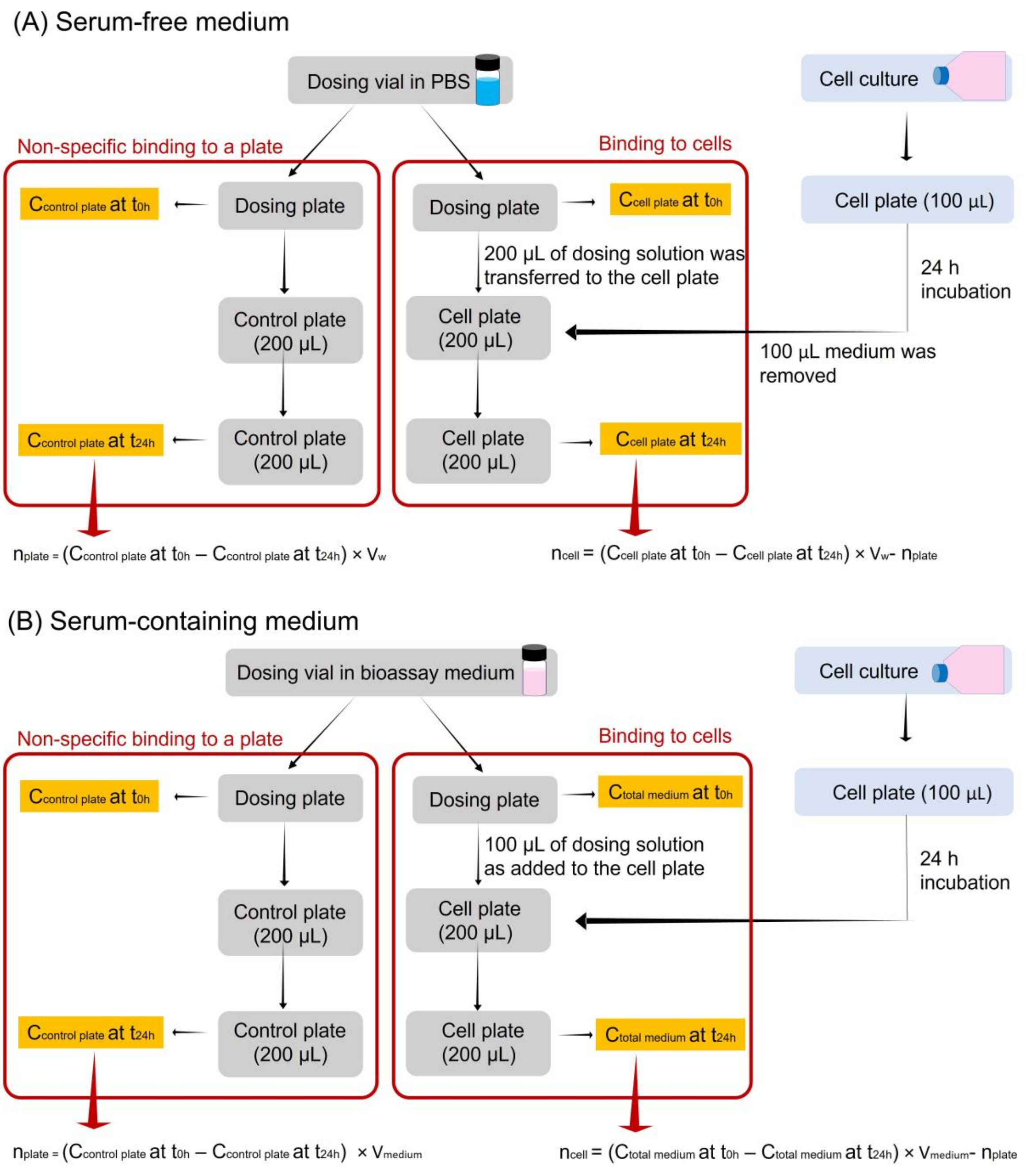

Figure S1. Experimental work flow of cell-water partition coefficient ($K_{cell/w}$) measurement in the serum-free medium (PBS) (A) and in the serum-containing (10% fetal bovine serum supplement) medium (B). $C_{control\ plate}$ is the concentration measured in the control plate without cells. $C_{cell\ plate}$ and $C_{total\ medium}$ are the concentrations measured in the plate containing cells but former concentration corresponds to the plate containing PBS (serum-free) and latter concentration corresponds to the plate with the assay medium (serum-containing). All initial concentrations (t_{0h}) are measured in the remnant of the dosing plate while the concentrations at t_{24h} were quantified after cell confluency detection. The chemical amounts sorbed in the test plate (n_{plate}) and bound in the cells (n_{cell}) are then calculated from the quantified concentrations with corresponding volume of the PBS (V_w) and medium (V_{medium}).

Table S4**.** Volume fractions of protein, lipids, and water in medium used in AREc32 and AhR-CALUX assay. Data were taken from Qin et al.[1]

Assay	Assay medium	$Vf_{protein,medium}$ [mL/L]	$Vf_{lip,medium}$ [mL/L]	$Vf_{w,medium}$ [L/L]
AREc32 assay	90% DMEM+10% FBS	3.02	7.44×10^{-2}	0.997
AhR-CALUX assay	90% DMEM+10% FBS	3.02	7.44×10^{-2}	0.997

Table S5**.** Volume fractions of protein, lipids, and water in cells used in AREc32 and AhR-CALUX bioassays. Data were taken from Qin et al.[1]

Assay	Cell line	$C_{protein}$ [mL/10^6 cell]	$C_{lip,cell}$ [mL/10^6 cell]	$V_{total,cell}$ [mL/10^6 cell]	$Vf_{protein,cell}$ [mL/L]	$Vf_{lip,cell}$ [mL/L]	$Vf_{w,cell}$ [L/L]
AREc32 assay	MCF7	1.87×10^{-4}	4.09×10^{-5}	1.00×10^{-2}	18.7	4.1	0.977
AhR-CALUX assay	H4IIE	9.00×10^{-5}	1.46×10^{-5}	3.53×10^{-3}	25.5	4.1	0.970

Table S6**.** Critical micelle concentration (CMC in mM) of 1-alkyl-3-methylimidazolium chlorides (IM1-R Cl) and benzalkonium chlorides (N11-R-1Ph Cl) determined by a fluorescent probe (9-diethylamino-5-benzo [a]phenoxazinone, Nile red). Tests were performed in PBS buffer (pH=7.4) at 25 °C.

Compounds	CMC [mM]	Compounds	CMC [mM]
IM1-10 Cl	21.42[2]	N11-10-1Ph Cl	9.6
IM1-12 Cl	2.88[2]<br>1.9	N11-12-1Ph Cl	1.0
IM1-14 Cl	0.2	N11-14-1Ph Cl	0.1
IM1-16 Cl	0.03*	N11-16-1Ph Cl	0.022*

[2]Kowalska et al. 2022

* In the plastic sorption experiment, 50% of IM1-16 Cl was lost at concentration below the CMC and approximately 70% of N11-16-1Ph Cl was lost at similar concentration due to sorption to plastic. Therefore, thes values should be treated as approximations of the CMC.

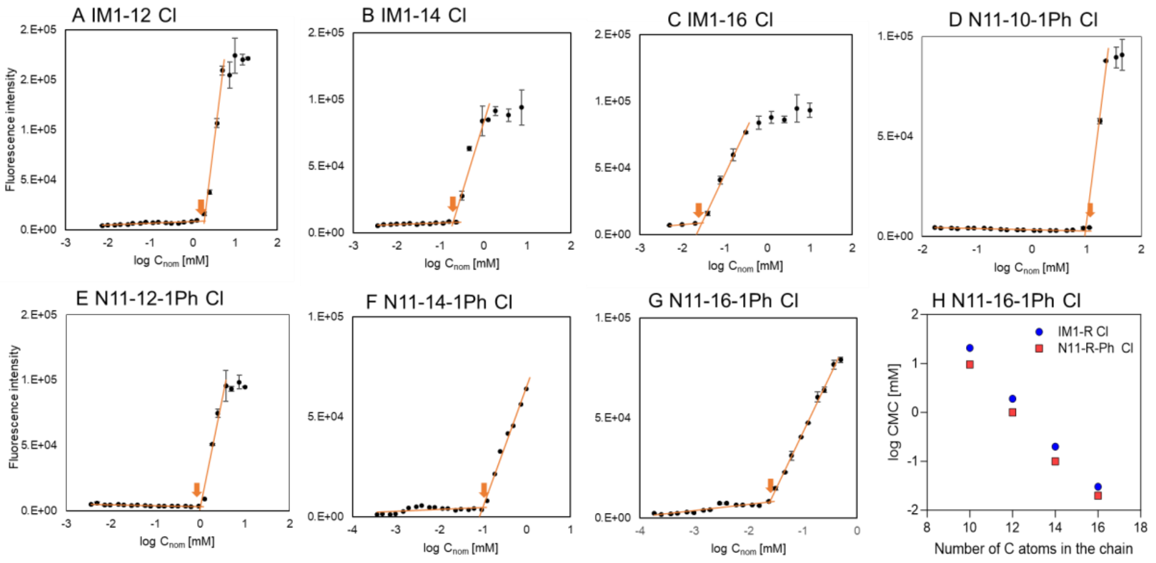

Figure S2. (A-G) The change of the fluorescence intensity against test concentration of cations of ionic liquids (ILs) in PBS buffer (pH = 7.4) at 25 °C. The inflection point on the fluorescence-concentration curve indicates the onset of micelle formation (CMC). (H) A linear decrease of CMC with increasing side chain length in IM1-R Cl and N11-R-1Ph Cl. A linear regression model was fitted to each group of cation, giving $R^2 > 0.99$.

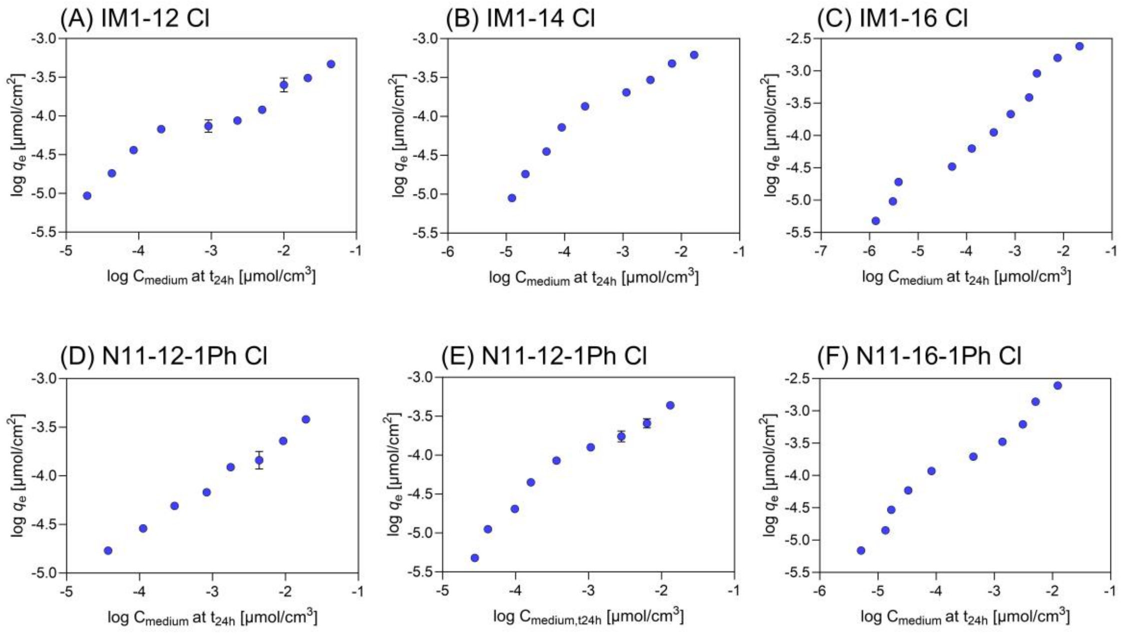

Figure S3. Sorption isotherms of IM1-R Cl and N11-R-1Ph Cl (R:12-16) to a 96-well plate in the serum-free medium.

Table S7. Membrane lipid-water partition constants (K_{mw}) and albumin-water partition constants ($K_{albumin/w}$) of cations of ionic liquids (ILs). Experimental values were obtained either in this study or collected from literature. Values in italic indicate predicted data (model denoted in the column 'source'). If more than one data point was available for $K_{albumin/w}$, the mean value was used.

Compounds	Log K_{mw} [L_w/L_{lip}]	Source	log $K_{albumin/w}$ [$L_w/L_{protein}$] (pH 7.4, 25 °C)	Source
IM1-2 Cl	0.92 ± 0.04	This study	-1.31	Satish et al. 2017[3]
IM1-4 Cl	1.07 ± 0.04	This study	-1.59<br>-0.62<br>0.20	Satish et al. 2017[3]<br>Huang et al. 2013[4]<br>Shu et al. 2011[5]
IM1-6 Cl	1.55 ± 0.02	This study	-0.33	Huang et al. 2013[4]
IM1-2Ph Cl	1.30 ± 0.01	This study	-	
IM1-1Ph-4Me Cl	*1.39*	Predicted by COSMOmic	-	
P4444 Br	<1.50	This study	1.11 ± 0.06	This study
N4444 Br	<1.50	This study	0.86 ± 0.05	This study
N1118 Br	2.18	Timmer and Droge, 2017[6]	-	
N111-10 Br	3.34	Timmer and Droge, 2017[6]	-	
N111-12 Cl	4.35	Timmer and Droge, 2017[6]	2.44	Zhou et al. 2013[7]
N111-14 Cl	5.46	Timmer and Droge, 2017[6]	-	
N11-6-1Ph Cl	2.12	Timmer and Droge, 2017[6]	-	
N11-8-1Ph Cl	3.11	Timmer and Droge, 2017[6]	-	
Py2 Cl	*-0.01*	Predicted by COSMOmic	-	
Py4 Cl	1.11 ± 0.02	This study	-	
Py6 Cl	1.50 ± 0.01	This study	0.64 ± 0.04	This study
Py8 Cl	2.28	Dołżonek et al. 2017[8]	1.09 ± 0.03	This study
Py12 Cl	4.89	Timmer and Droge, 2017[6]	-	
Py4-2Me cl	*0.68*	Predicted by COSMOmic	-	
Py4-3Me Cl	1.29 ± 0.01	This study	-	
Py4-4Me Cl	*0.75*	Predicted by COSMOmic	-	
Py6-3Me cl	-	-	1.01 ± 0.07	This study
Py8-3Me Cl	2.34	Dołżonek et al. 2017[8]	1.00 ± 0.06	This study
Py8-4Me Cl	2.41 ± 0.07	This study	-	
Py2-4NMe2 Br	1.19 ± 0.01	This study	-	
Py4-4NMe2 Cl	1.33 ± 0.04	This study	-	
Py6-4NMe2 Cl	1.79 ± 0.04	This study	1.15 ± 0.06	This study
Pip1-4 Br	1.15 ± 0.01	This study	-	
Pyr1-4 Br	1.06 ± 0.02	This study	-	
Pyr1-8 Br	2.18	Dołżonek et al. 2017[8]	-	

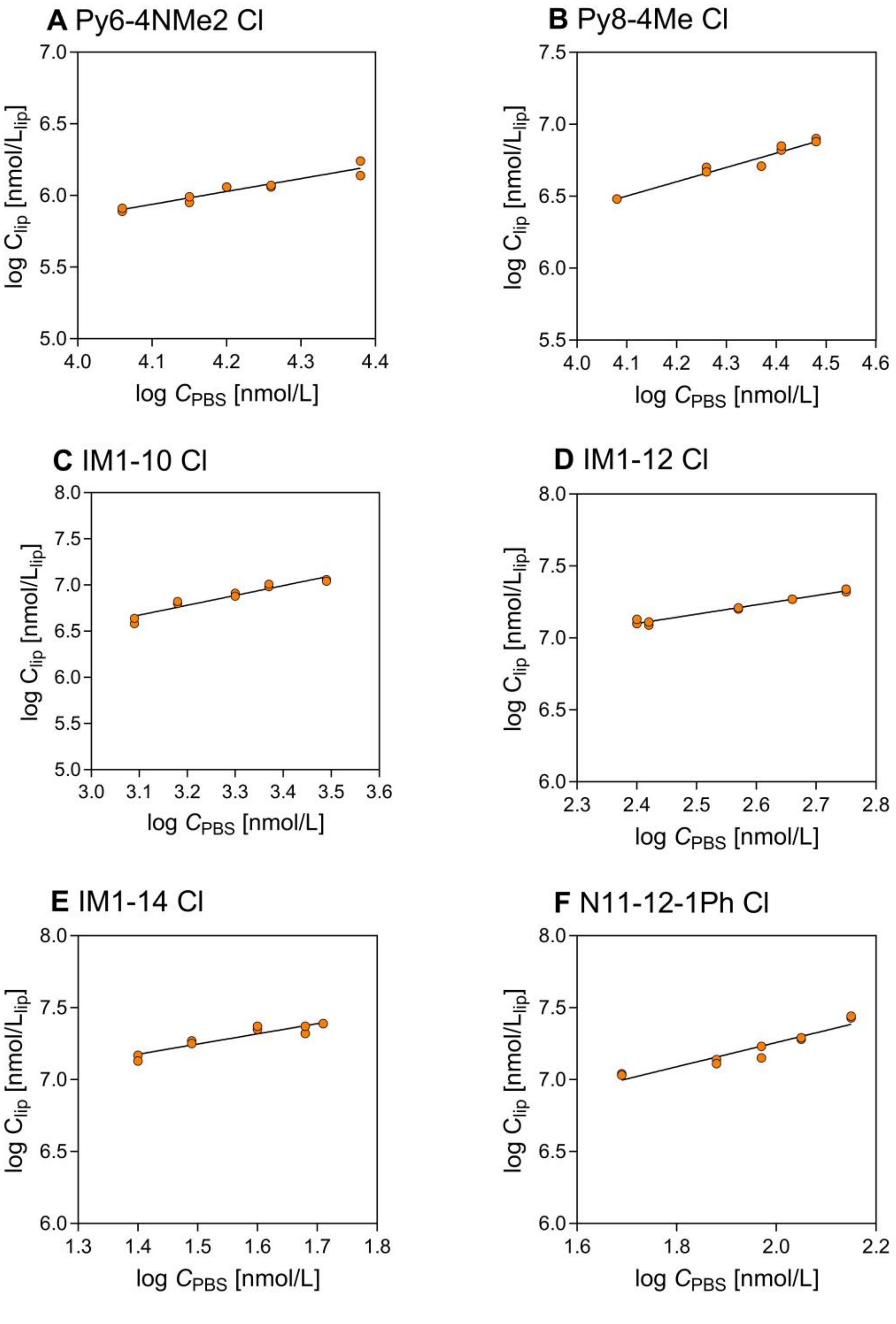

Figure S4. Sorption isotherms of six IL cations on lipid membrane made of egg phosphatidylcholine (POPC) obtained using the standard TRANSIL Intestinal Absorption kit. Concentrations bound on lipid membrane ($C_{lipid,TRANSIL}$) were plotted against the aqueous concentrations measured in the supernatant ($C_{supernatant,TRANSIL}$) at the end of the test. Linear regression lines were fitted into the isotherm

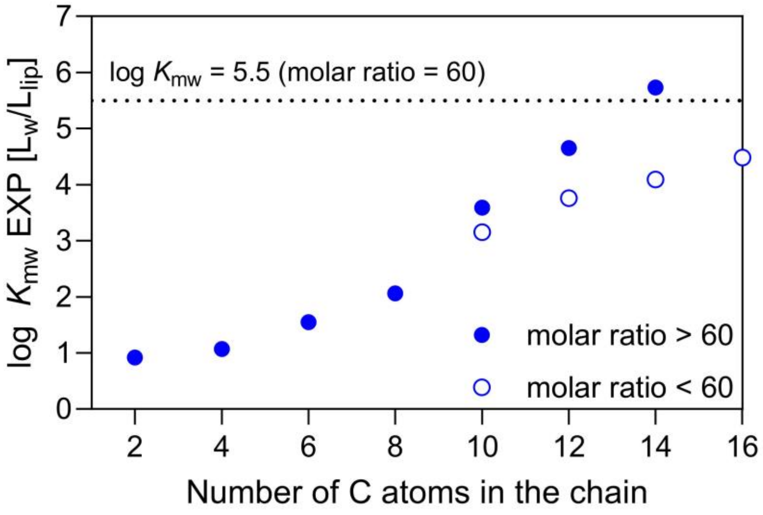

Figure S5. The experimental log K_{mw} of 1-methyl-3-alkylimidazolium chlorides (IM1-R Cl, R: 2-16) measured in this study or collected from literature were plotted against carbon number in the alkyl side chain. Log K_{mw} values obtained in tests in which a lipid/sorbed compound molar ratio was kept above 60 are remarked as closed circles while log K_{mw} from tests where the molar ratio was below 60 are denoted as open circles. The dotted line indicates the log K_{mw} where a lipid/sorbed compound ratio equals 60.[6]

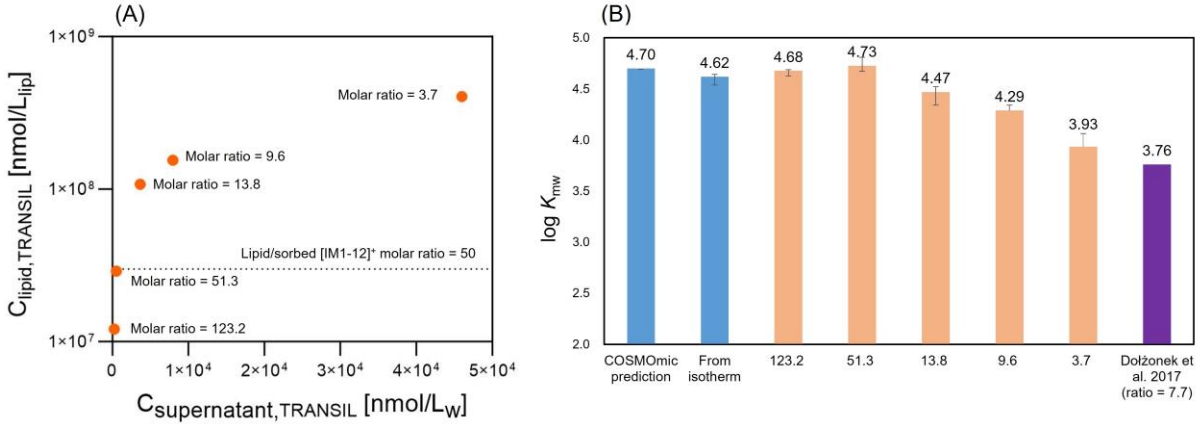

Figure S6. (A) A sorption isotherm of IM1-12 Cl on SSLMs obtained with different lipid/sorbed compound molar ratio (4 – 123); (B) log K_{mw} value obtained as a slope of the part of the isotherm where lipid/sorbed compound molar ratio > 60 or predicted by COSMOmic (in blue) together with log K_{mw} that were measured at different lipid/sorbed compound molar ratio conditions (in organge) as well as K_{mw} value collected from Dołżonek et al. 2017[8] where a lipid/[IM1-12]$^+$ molar ratio was estimated to be 7.7 (in purple).

Text S1. Polyparameter Linear free energy relationship (pp-LFERs) models to predict octanol-water partitioning (K_{ow}), chromatographic capacity factor (k_0) and membrane lipid-water partition coefficients (K_{mw}) and the calculation of K_{mw} by COSMOmic.

A pp-LFER is a multiple linear regression model and has been used to characterize partitioning into various environmental and biological phases. The model accounts for several molecular interactions between the solutes and solvents/solid phases. Experimental octanol-water partition coefficient (log K_{ow}) values were collected from literature and preferably used over predicted values. When no experimental value was found, the log K_{ow} was predicted by a pp-LFER model developed by Cho et al. 2018[9] (eq A1). All retrieved LFER solute descriptors of single cations and anions of ILs are included in Table S5. As a result, experimental log K_{ow} for 14 compounds and the predicted log K_{ow} for 12 compounds were obtained and are summarized in Table S6.

$$\log K_{ow} = 0.932\ E_c + 2.881\ V_c - 0.811\ B_a + 1.538\ V_a - 5.609 \qquad \text{(A1)}$$

A HPLC-derived lipophilicity parameter that was determined based on chemicals' retention behavior on a reverse phase HPLC column (log k_0) was correlated with log K_{mw} for 26 ILs.[10] The experimental log k_0 was collected from literature and preferably used while a pp-LFER model (eq A2) was used to predict log k_0 for chemicals for which measurement data were not available (listed in Table S6) [10,11].

$$\log k_0 = -1.929\ B_c + 1.95\ V_c - 2.001 \qquad \text{(A2)}$$

A pp-LFER equation extended for ionic species by Abraham and Acree[12] was used in combination with fitting procedure proposed by Bittermann et al. 2016 to predict K_{mw}.[13] This is extended for the description of the partitioning of ions via the inclusion of a j^+J^+ term for cations and j^-J^- term for anions. Since in our study we only delt with partitioning cations, the term j^-J^- for anions (halides) was omitted[14,15] leading to the eq A3:

$$\log K_{mw} = 0.96\ E - 0.60\ S - 0.41\ A - 2.64\ B + 2.74\ V - 1.00\ J^+ \qquad \text{(A3)}$$

As a result, log K_{mw} of 21 cations of ILs were predicted by Eq. (A3) and listed in Table S6.The K_{mw} was predicted using the COnductor-like Screening MOdel for Realistic Solvation (COSMO-RS) theory to describe the partitioning of compounds into the lipid bilayer by virtually deconstructing the complex structure of such membranes into multiple homogeneous layers (COSMOmic).[16] The predicted K_{mw} by COSMOmic for 21 ILs (cation) were obtained from literature and 8 compounds were predicted as described in Bae et al. (2024) (Table S6).[17] Briefly, by using COSMOtherm software (COSMOtherm Version 18.0.2) with TURBOMOLE (TURBOMOLE V7.3), DFT/COSMO calculation based on BP density functional theory with triple-zeta valence polarization (TZVP) was performed to generate the structure files of cations of ILs. The prediction was based on a published 1-palmitoyl-2-oleoyl-*sn*-glycero-3-phosphochloine (POPC) micelle structure. In COSMOmic calculation, dipole interactions of charged ions were separately considered by a correction term. An offset of 0.32 log units was deducted from COSMOmic calculations according to Bittermann et al. 2016.[13]

Table S8. Polyparameter Linear free energy relationship (pp-LFER) solute descriptors of single ions of ionic liquids (E: Excess molar refraction; S: dipolarity/polarizability; A: hydrogen bonding acidity; B: hydrogen bond basicity; V: McGowan volume; J: ionic interaction).

Abbreviation	Ion	E (ion)	S (ion)	A (ion)	B (ion)	V (ion)	J (ion)	Ref.
IM1-2	cation	0.45	2.35	0.55	-0.03	0.99	0.81	[1]
IM1-4	cation	0.47	2.53	0.59	-0.02	1.27	0.99	[1]
IM1-6	cation	0.49	2.69	0.62	-0.02	1.55	1.14	[1]
IM1-8	cation	0.50	2.84	0.64	-0.01	1.84	1.30	[1]
IM1-10	cation	0.53	3.02	0.67	-0.01	2.11	1.47	[1]
IM1-12	cation	1.37	1.62	0.65	-0.03	2.45	-	[2]
IM1-14	cation	0.58	3.31	0.71	0.00	2.67	1.76	[1]
IM1-16	cation	0.57	3.46	0.74	0.01	2.96	1.92	[1]
IM1-2Ph	cation	1.09	3.11	0.79	-0.02	1.59	1.17	[1]
N1118	cation	0.83	1.45	0.45	-0.01	1.77	N/A	[2]
N111-10	cation	0.95	1.52	0.45	-0.01	2.07	N/A	[2]
N111-12	cation	1.07	1.59	0.45	0.02	2.37	N/A	[2]
N111-14	cation	1.19	1.65	0.45	0.03	2.67	N/A	[2]
N11-10-1Ph	cation	0.47	3.44	0376	0.00	2.64	1.95	[1]
N11-12-1Ph	cation	0.49	3.60	0.77	0.01	2.91	2.10	[3]
N11-14-1Ph	cation	1.65	1.98	0.47	0.07	3.18	N/A	[4]
N11-16-1Ph	cation	1.99	2.06	0.53	0.04	3.32	N/A	[4]
Py2	cation	0.51	2.56	0.67	-0.04	0.96	1.03	[1]
Py4	cation	0.53	2.70	0.66	-0.03	1.24	1.15	[1]
Py6	cation	0.55	2.86	0.69	-0.02	1.52	1.31	[1]
Py8	cation	0.56	3.00	0.72	-0.02	1.81	1.47	[1]
Py4-3Me	cation	0.55	2.68	0.49	0.00	1.38	1.09	[1]
Py6-3Me	cation	0.56	2.85	0.53	0.01	1.66	1.26	[1]
Py8-3Me	cation	0.61	3.01	0.55	0.01	1.93	1.41	[1]
Py8-4Me	cation	0.59	2.97	0.52	0.01	1.94	1.38	[1]
Pip1-4	cation	0.26	2.59	0.50	0.02	1.48	1.15	[1]
Pyr1-4	cation	0.20	2.61	0.60	0.01	1.37	1.20	[1]
Pyr1-8	cation	0.25	2.91	0.64	0.02	1.92	1.50	[1]
Py2-4NMe2	cation	0.64	2.65	0.37	0.01	1.33	1.01	[1]
Py4-4NMe2	cation	0.66	2.8	0.38	0.02	1.61	1.15	[1]
Py6-4NMe2	cation	0.67	2.96	0.41	0.03	1.9	1.32	[1]
Cl^-	anion	0.29	2.20	-0.08	2.31	0.23	2.05	[3]
Br^-	anion	0.52	2.90	-0.07	1.93	0.27	1.78	[3]

[1] Cho et al. 2016,[18] [2] Cho et al. 2011,[11] [3] Cho et al. 2018,[9] [4] Cho et al. 2013[19]

Table S9. Octanol-water partition constant (log K_{ow}) and chromatographic capacity factor (log k_0) that were collected from literature or predicted in this study, as well as membrane-water partitioning (log K_{mw}) predicted by a pp-LFER or COSMOmic and the experimental log K_{mw}.

Compounds	Correlation parameter		log K_{mw} (predicted)		log K_{mw} (experimental)[b]
	log K_{ow}	Log k_0	pp-LFER[a]	COSMOmic	
IM1-2 Cl	-3.85[a]	0.22[g]	1.29	0.07[h]	0.92
IM1-4 Cl	-2.4[c]<br>-2.77[c]	0.67[g]	1.73	0.78[h]	1.07
IM1-6 Cl	-1.73[c]	1.24[g]	2.26	1.70[h]	1.55
IM1-8 Cl	-0.6[c]	1.85[g]	2.78	2.61[a]	2.06[i]
IM1-10 Cl	0.31[c]	2.37[g]	3.26	3.67[a]	3.59
IM1-12 Cl	1.26[a]	2.87[a]	-	4.64[a]	4.65
IM1-14 Cl	1.10[a]	4.60[g]	4.34	5.62[a]	5.73
IM1-16 Cl	1.93[a]	6.08[g]	-	6.66[a]	-
IM1-2Ph Cl	-1.99[c]	1.01[g]	2.60	1.51[h]	1.30
N1118 Br	-0.90[a]	1.45[a]	-	2.18[j]	2.18[j]
N111-10 Br	0.08[a]	2.04[a]	-	3.30[j]	3.34[j]
N111-12 Cl	0.69[e]	2.58[a]	-	4.47[j]	4.35[j]
N111-14 Cl	1.67[a]	3.15[a]	-	5.61[j]	5.46[j]
N11-6-1Ph Cl	-	-	-	1.87[j]	2.12[j]
N11-8-1Ph Cl	-	-	-	2.96[j]	3.11[j]
N11-10-1Ph Cl	1.04[c]	2.93[g]	3.86	4.05[j]	4.01[j]
N11-12-1Ph Cl	1.73[c]	3.49[g]	4.34	4.93[h]	5.26
N11-14-1Ph Cl	-	4.06[g]	-	5.92[h]	-
N11-16-1Ph Cl	-	4.40[g]	-	6.94[a]	-
Py4 Cl	-2.82[c]	0.58[g]	1.44	0.66[h]	1.11
Py6 Cl	-2.23[a]	1.01[a]	1.95	1.58[h]	1.50
Py8 Cl	-0.72[c]	1.56[a]	2.48	2.53[a]	2.28[k]
Py12 Cl	-	-	-	4.77[f]	4.89[j]
Py4-3Me Cl	-2.62[c]	0.73[g]	1.91	0.79[h]	1.29
Py8-3Me Cl	-0.99[a]	1.74[a]	2.91	2.67[a]	2.34[k]
Py8-4Me Cl	-0.98[a]	1.77[a]	2.99	2.63[a]	2.40
Pip1-4 Br	-2.27[a]	0.68[g]	1.82	0.57[h]	1.15
Pyr1-4 Br	-2.63[a]	0.57[g]	1.39	0.49[h]	1.06
Pyr1-8 Br	-0.59[f]	1.87[g]	2.44	-	2.18[k]
Py2-4NMe2 Br	-2.32[a]	0.51[g]	1.99	0.31[h]	1.19
Py4-4NMe2 Cl	-2.13[c]	1.08[g]	2.51	1.13[h]	1.33
Py6-4NMe2 Cl	-1.15[c]	1.80[g]	3.01	2.04[h]	1.83

[a]calculated in this study as described above,; [b]measured in this study unless stated otherwise,;[c]Cho et al. 2011,;[11] [d]Lee and Lee, 2009,;[20] [e]Hodges et al. 2019,;[21] [f]Cho et al. 2018,;[9] [g]Ranke et al. 2007;,[10] [h]Bae et al. 2024.[17]; [i]Stolte et al. 2007,;[22] [j]Timmer and Droge, 2017[6],; [k]Dołżonek et al. 2017[8]

Text S2. Comparison of four approaches to predict membrane lipid-water partitioning (log K_{mw}) for cations of ionic liquids.

The accuracy of models predicting membrane lipid-water partition coefficient (K_{mw}) was assessed using data from this study and literature as described in Text S1. First, a correlation of K_{mw} and K_{ow} for 26 cations of ionic liquids (ILs) yields a linear regression equation with a good linearity (log K_{mw} = 0.82 × log K_{ow} + 3.12, R^2 = 0.85, Figure S7A) and a decent predictive power (RMSE = 0.43). However, the absolute values of K_{mw} were up to several orders of magnitude higher than the corresponding K_{ow} values as denoted by the intercept. In contrast, the K_{mw} agrees well with the K_{ow} for 181 neutral organic compounds in terms of absolute magnitude of these values with differences being 0.4 log units on average.[23] This can be attributed to the fact that octanol mimics the chemical environment of the region of lipid membrane where most neutral compounds reside.[13,23] However, octanol is poorly suited to represent the anisotropic structure of membrane lipids, a major sink for charged compounds, which could lead to a substantial underestimation of the lipophilicity of ions.[24,25] Moreover, the surface-active properties of IL cations such as accumulation at interphases or formation of emulsions pose difficulties in K_{ow} measurement,[26] resulting in less reliable K_{ow}. The large differences between the absolute values of the K_{ow} and the K_{mw} for cations of ILs also demonstrate that the K_{ow} values are not an adequate measure of hydrophobicity of IL cations (and charged compounds in general[13]) and should not be used to compare them against thresholds established for neutral organics (e.g., to determine the tendency for bioaccumulation, sorption to biological membranes or plastic).

The chromatographic capacity factor (log k_0) generated using a reverse-phase chromatographic column has a generally good linear correlation with the measured log K_{mw} for 26 IL cations (log K_{mw} = 1.34 × log k_0 + 0.21, R^2 = 0.82, Figure S7B). Nevertheless, there is a clear tendency for log k_0 to underestimate membrane affinity, especially for more hydrophobic compounds (log K_{mw} > 2, Figure S7B), resulting in a slightly lower accuracy (RMSE = 0.54). With a HPLC-based screening, a large amount of data can be generated in a short time while additionally providing a wider measuring range. Cho et al. (2013) developed a high accuracy pp-LFER model for log k_0 prediction (R^2 = 0.99, RMSE = 0.14, n = 63).[19] Thus, theoretically the K_{mw} of IL cations can be obtained by a correlation with log k_0 (either measured or predicted using pp-LFER), yet this approach should be applied with caution for more hydrophobic compounds.

A pp-LFER model developed by Abraham and Acree[12] and a fitting procedure proposed by Bittermann et al. (2016) (eq A1) was used to predict the K_{mw} for 21 IL cations (Figure S7C).[13] While this model has performed rather poorly for cationic and anionic compounds (cationic compounds: R^2=0.37, RMSE = 1.12, anionic compounds: R^2 = 0.59, RMSE = 1.26) in the past,[13] it showed better results for IL cations in this study (R^2 = 0.86, RMSE = 0.73) with a few exceptions showing one order of magnitude deviation between measured and predicted K_{mw} (i.e., IM1-14 Cl, IM1-2 Ph, Py4-4NMe2 Cl, Py6-4NMe2 Cl in Table S7). In the work of Bittermann et al. (2016), the LFER solute parameters for ionic compounds were re-calculated based on parameters of corresponding neutral compounds.[13] On the other hand, solute parameters for ILs determined by Cho et al. (2015) were derived by prediction models that were fine-tuned for ionic compounds,[27] which may explain the superior performance of the pp-LFER model

for ILs. Nevertheless, the predictive power of pp-LFER approach was still worse than other three methods (RMSE = 0.73 in Figure S7C).

COSMOmic was found to be well suited to predict K_{mw} of ionic compounds, with no additional information required beyond the compound structure and the model membrane.[13] COSMOmic predicted the K_{mw} of 30 IL cations with high accuracy (R^2 = 0.92, RMSE = 0.36) (Figure S7D). The largest deviation from the experimental K_{mw} was observed for the most hydrophilic compounds (difference was 0.85 and 0.88 in IM1-2 Cl and Py2-4NMe2 Cl, respectively), and excluding such compounds yielded the same R^2 but a lower RMSE = 0.29. Indeed, for hydrophilic compounds (the alkyl chain containing ≤ 4 carbon atoms, log K_{mw} ≤ 1.0), the slope of experimental vs. predicted log K_{mw} becomes shallower, resulting in a concave curve (Figure S7D). The exact nature of lower increase of membrane affinity per CH_2 fragment observed in the side chain of hydrophilic compounds remains unknown. It is possible that this phenomenon is an experimental artifact caused by the very low affinity of hydrophilic ions for the membrane. Nevertheless, COSMOmic exhibited the highest accuracy compared to other aforementioned approaches. This can be attributed to the fact that this model is able to describe the anisotropic nature of the membrane and explicitly takes into account the dipole moment of phospholipids.[13]

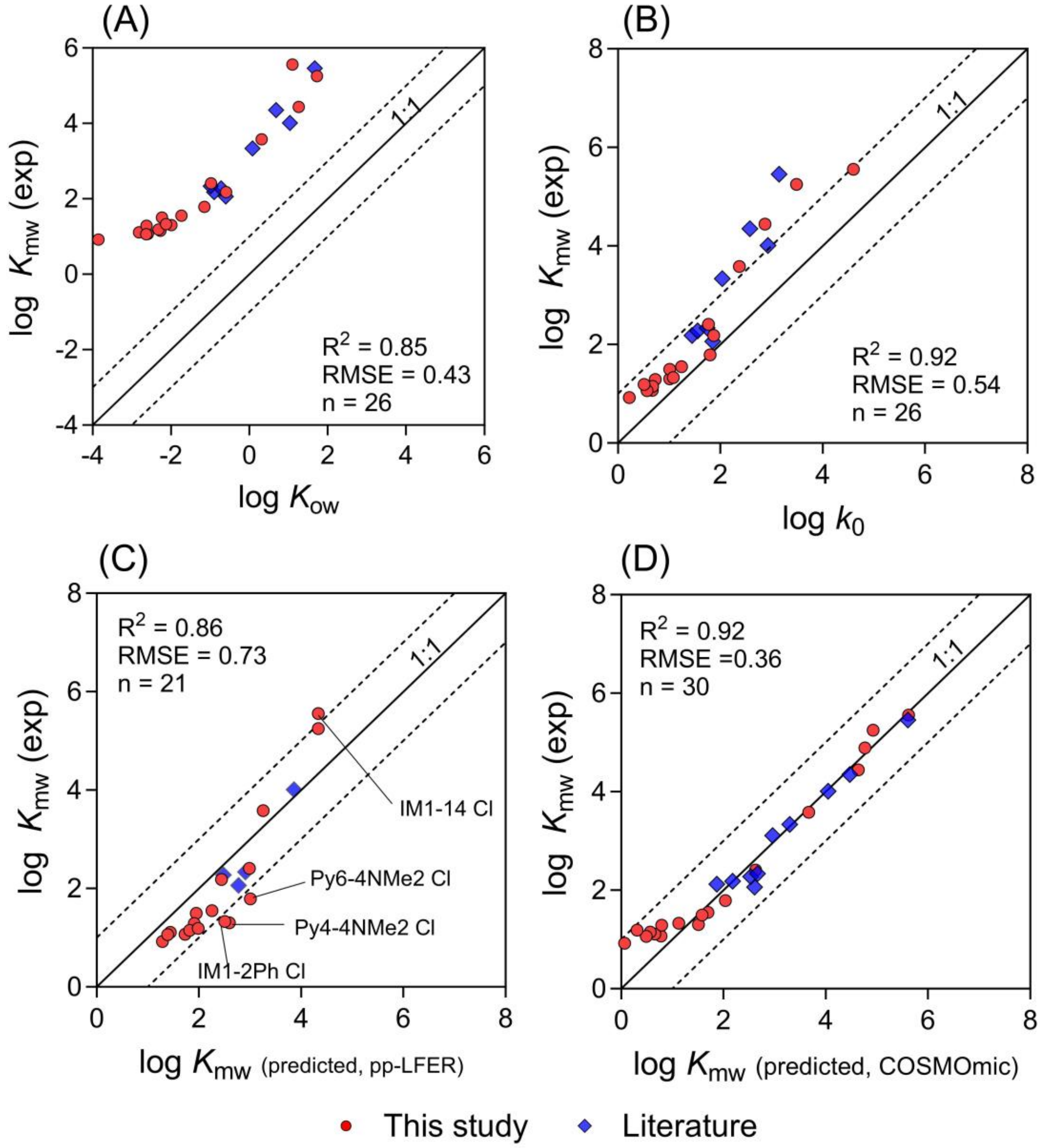

Figure S7. Correlations of the experimental log K_{mw} [L_w/L_{lip}] for IL cations with (A) octanol-water partition coefficients (log K_{ow}), (B) a HPLC-derived chromatographic retention factor (log k_0), (C) log K_{mw} predicted with pp-LFER model and (D) log K_{mw} predicted with COSMOmic. The one-to-one line (black, solid) represents an agreement between independent variables (x-axis) and the predicted log K_{mw} values, the dotted lines are the 1:10 and 10:1 line. The K_{mw} values measured in this study using the TRANSIL absorption kit are shown in red circles, while those compiled from multiple literature sources are remarked in blue diamonds.

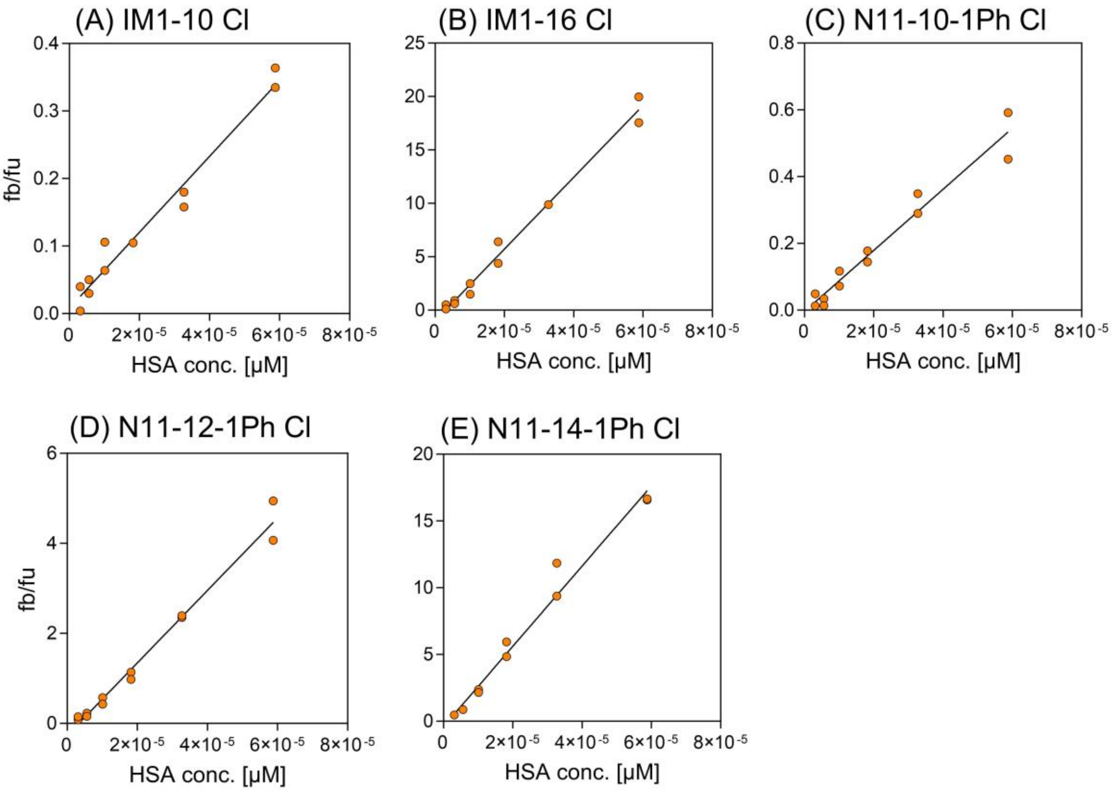

Figure S8. Sorption isotherms of five IL cations on standard TRANSIL^{XL} HSA Binding Kit. HSA concentration on x-axis is the amounts of HSA added in each well of the kit and fb/fu indicates the ratio of chemical fraction bound (fb) to HSA to unbound fraction (fu).

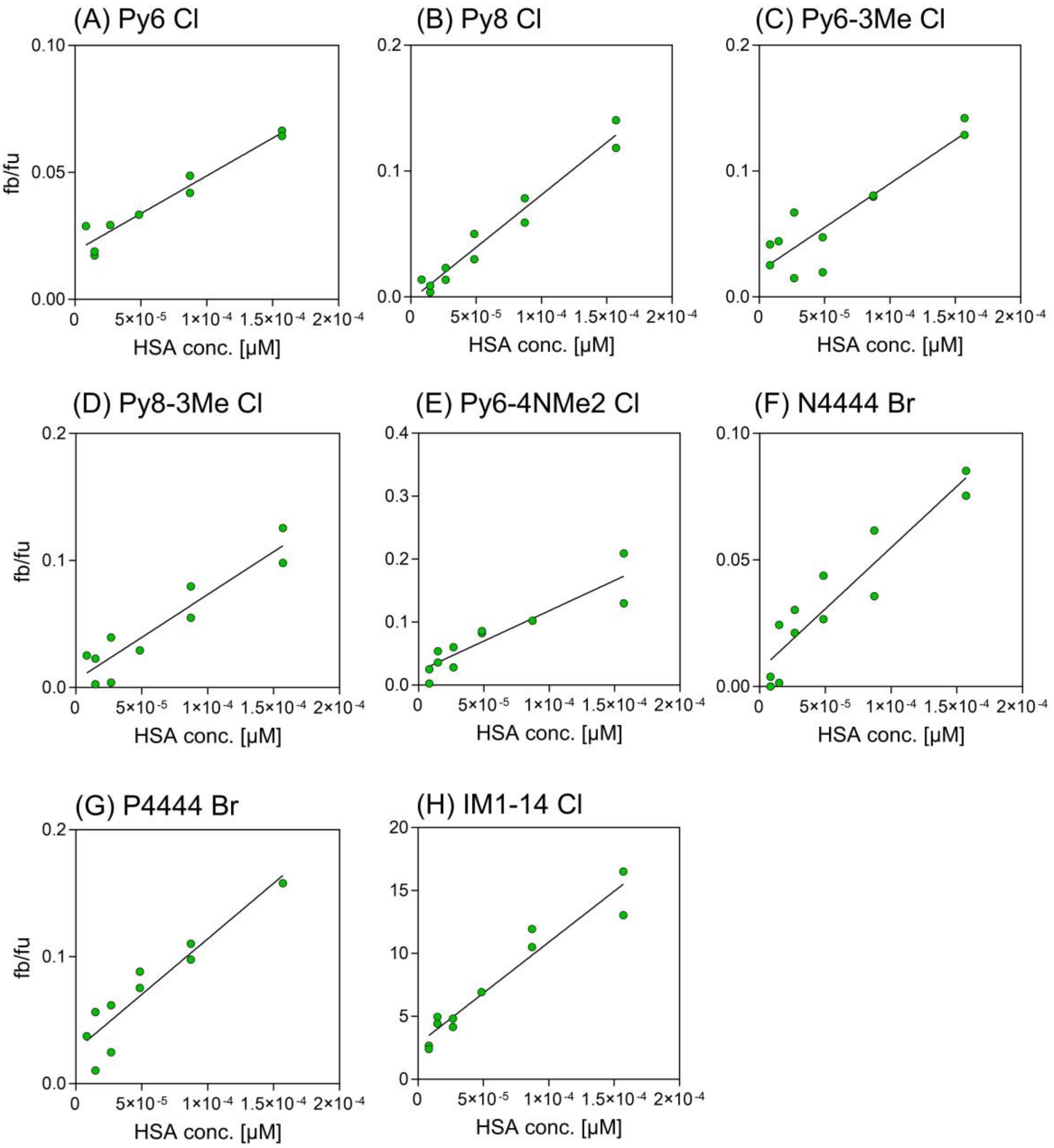

Figure S9. Sorption isotherms of eight IL cations on TRANSIL^{XL} HSA Binding Kit for low-affinity compounds. HSA concentration on x-axis is the amounts of HSA added in each well of the kit and fb/fu indicates the ratio of chemical fraction bound (fb) to HSA to unbound fraction (fu).

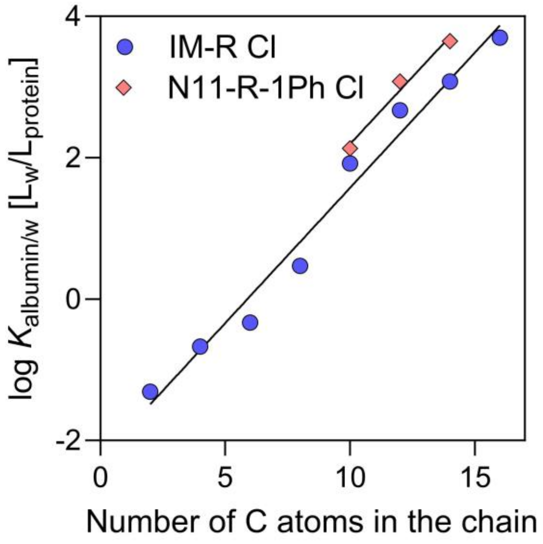

Figure S10. Experimental albumin-water partition coefficients (log $K_{albumin/w}$ in $L_w/L_{protein}$) of IL cations plotted against the number of carbon atoms in the side chain of IM1-R Cl and N11-R-1Ph Cl.

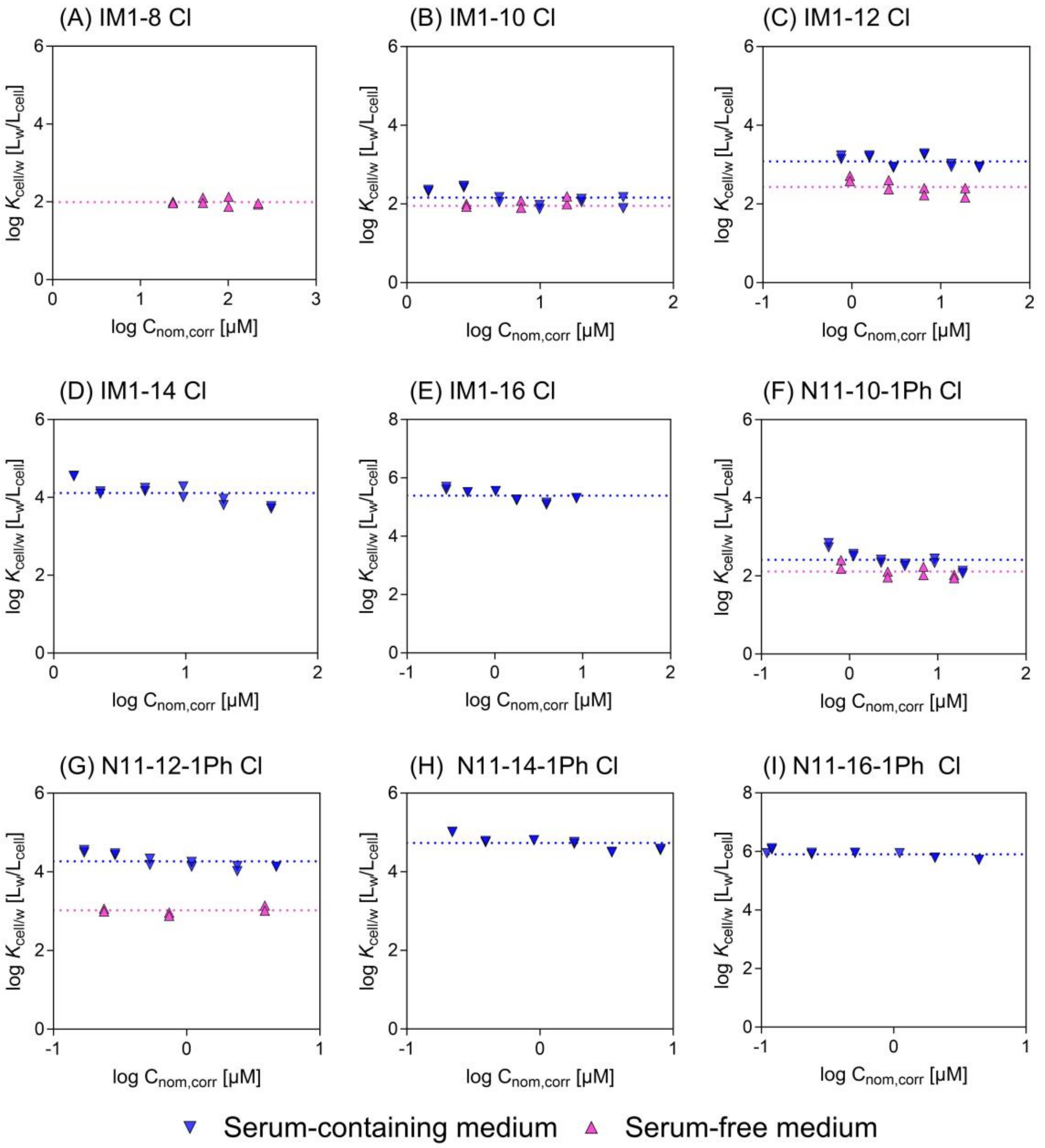

Figure S11. Partition coefficients between cell and water (log $K_{cell/w}$) for AREc32 cells in the serum-free medium (pink triangles) and serum-containing medium (blue inverted triangles) plotted as a function of the nominal concentration that are corrected with plate binding in eq 18 ($C_{nom,nom}$ in µmol/L). The dotted lines indicate the average of log $K_{cell/w}$ determined in each type of medium (denoted in matching color).

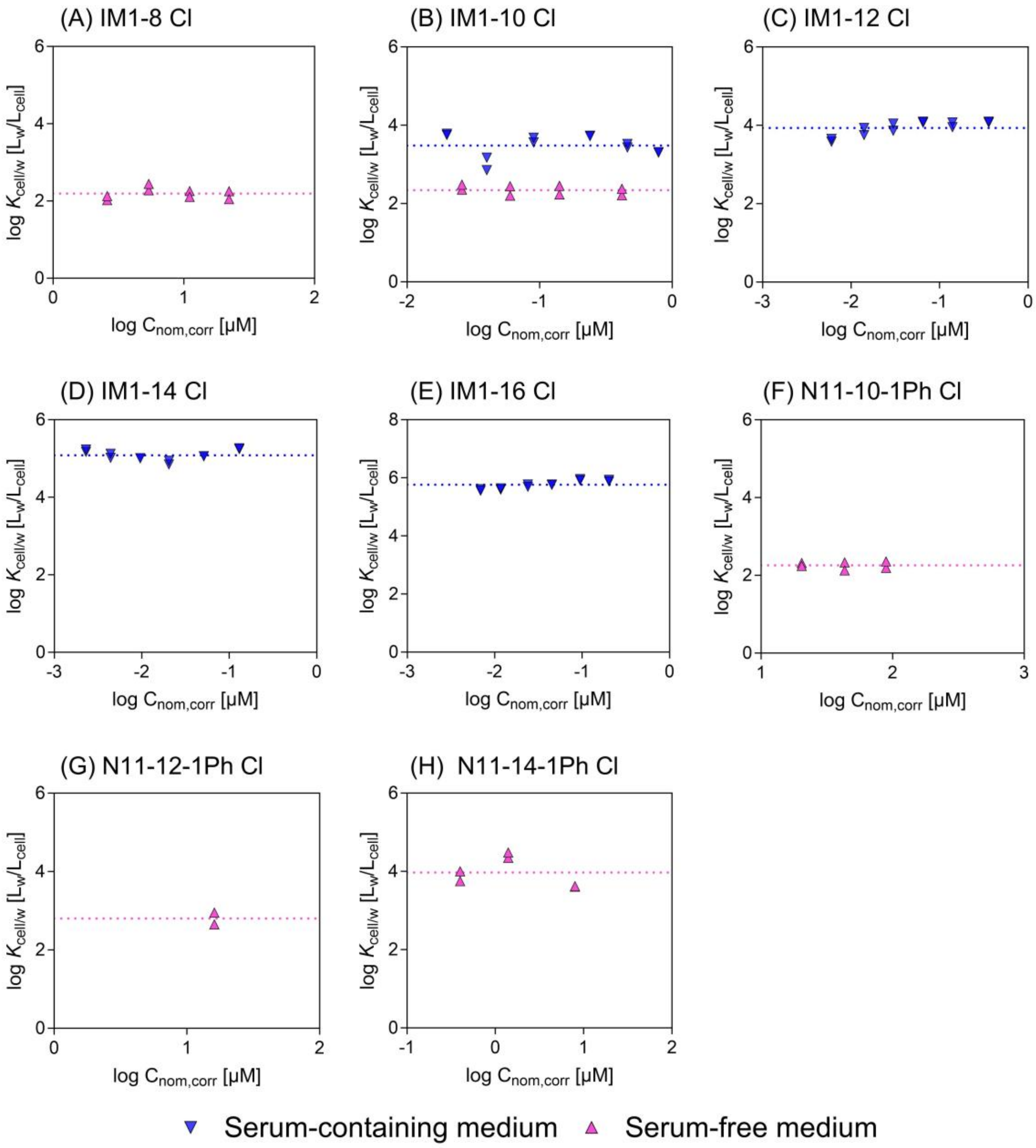

Figure S12. Partition coefficients between cell and water (log $K_{cell/w}$) for AhR-CALUX cells in the serum-free medium (pink triangles) and serum-containing medium (blue inverted triangles) plotted as a function of the nominal concentration that are corrected with plate binding in eq 18 ($C_{nom,corr}$ in μmol/L). The dotted lines indicate the average of log $K_{cell/w}$ determined in each type of medium (denoted in matching color).

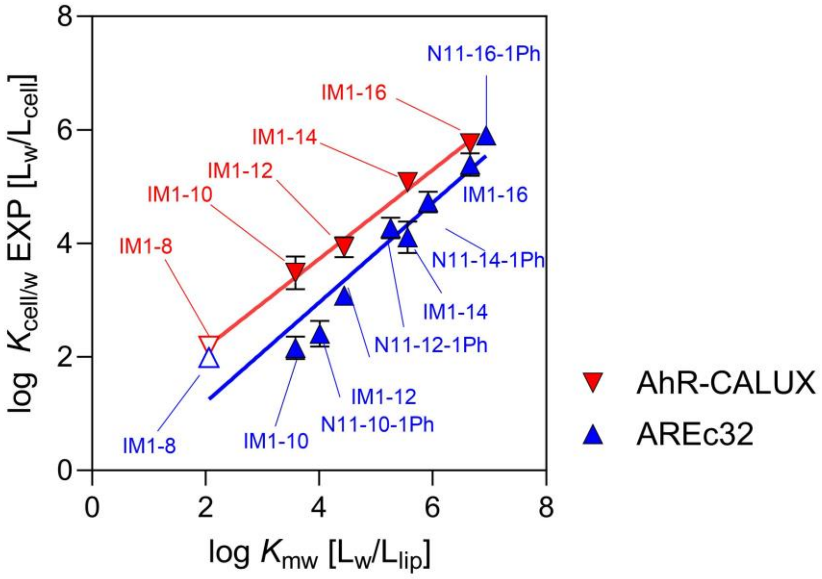

Figure S13. Linear relationships of membrane lipid-water (K_{mw}) and cell-water ($K_{cell/w}$) partition coefficients for AREc32 (blue triangles) and AhR-CALUX cells (red inverted triangles). The open symbols indicate the $K_{cell/w}$ of IM1-8 Cl, which were measured in the serum-free medium. A simple regression analysis was performed for each correlation, giving a model equation for the AREc32 cells (log $K_{cell/w}$ = 0.88 × log K_{mw} - 0.56, R^2 = 0.92) and AhR-CLAUX cells (log $K_{cell/w}$ = 0.78 × log K_{mw} + 0.59, R^2 > 0.92) cells.

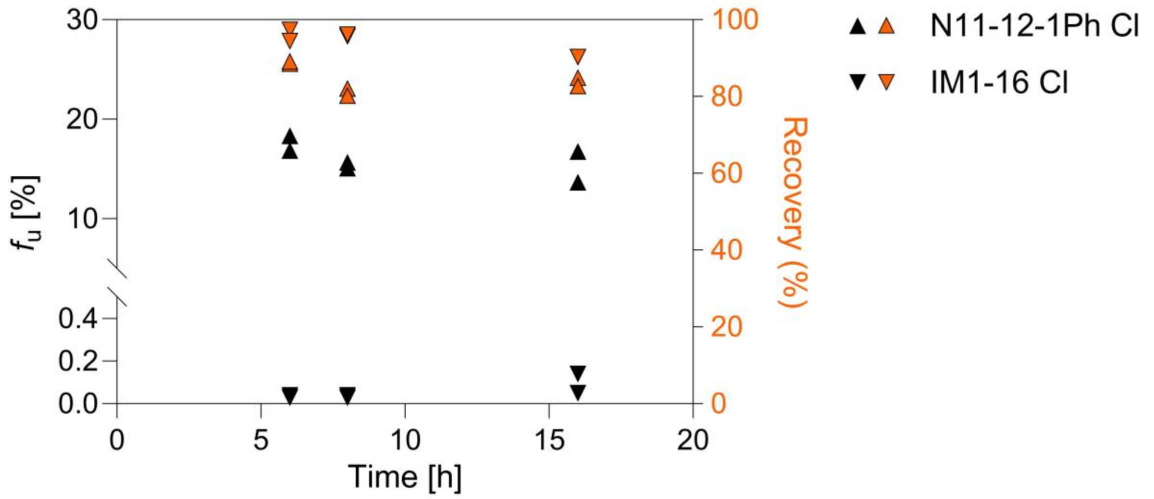

Figure S14. Changes of unbound fraction f_u (%), and recovery (%) of N11-12-1Ph Cl and IM1-16 Cl over time in a rapid equilibrium dialysis (RED) device. Symbols in black and in orange correspond to f_u (%) and recovery (%), respectively. Tests were performed in duplicates. The differences in f_u (%) between time points were analyzed by one-way analysis of variance (ANOVA) ($p > 0.05$).

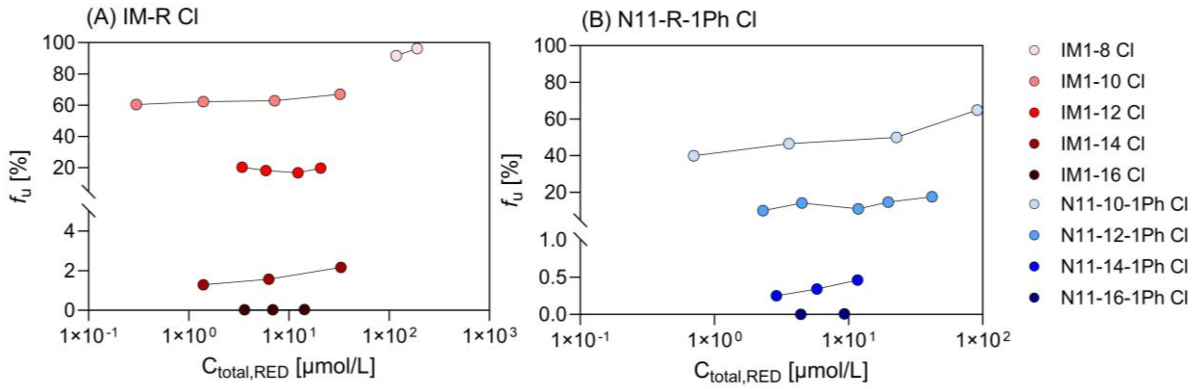

Figure S15 Measured unbound fraction f_u (%) for (A) IM1-R Cl and (B) N11-R-1Ph Cl in the rapid equilibrium dialysis (RED) system was plotted as a function of total concentrations added in the RED system ($C_{total,RED}$ μmol/L). Error bars are plotted for all points but are smaller than data markers.

Table S10. The average ± standard deviation of unbound fraction (f_u (%) eq 13)) and recovery (eq 14)) of the test compounds in the rapid equilibrium dialysis (RED) test. The lowest and highest values obtained within test range were given in bracket. f_u (%) in the cell-free medium containing 10% FBS predicted by a mass balance model (MBM) is given in the last column. Test concentration ranges (μmol/L) in bioassays (AREc32 and AhR-CALUX) are given for comparison.

ILs	Bioassay	Experiment using the RED			MBM
	Test range [μmol/L]	Test range [μmol/L]	f_u [%]	Recovery [%]	f_u [%]
IM1-8 Cl	0.5-230	117-190	94.0 ± 3.3 (91.7-96.3)	96.7 ± 6.3 (92.3-101.1)	98.6
IM1-10 Cl	0.02-42	0.3-32	63.1 ± 2.8 (60.4-67.0)	100.4 ± 3.4 (95.3-102.5)	65.3
IM1-12 Cl	0.01-27	3.4-21	18.7 ± 1.6 (16.7-25.3)	87.8 ± 5.2 (76.8-89.4)	22.4
IM1-14 Cl	0.01-44	1.4-33	1.68 ± 0.5 (1.3-2.2)	82.5 ± 2.7 (80.3-85.6)	3.2
IM1-16 Cl	0.01-9.4	3.6-14	0.034 ± 0.002 (0.03-0.04)	97.2 ± 2.0 (96.1-99.4)	0.28
N11-10-1Ph Cl	0.6-95	0.7-91	50.3 ± 10.6 (39.9-64.8)	91.2 ± 2.4 (87.7-92.7)	46.2
N11-12-1Ph Cl	0.2-24	2.3-42	13.4 ± 3.1 (10.0-17.6)	74.9 ± 8.3 (68.8-88.8)	5.6
N11-14-1Ph Cl	0.3-12	2.9-12	0.35 ± 0.1 (0.3-0.5)	84.4 ± 1.0 (83.6-85.5)	1.3
N11-16-1Ph Cl	0.2-6	4.4-9.3	0.0024 ± 0.0002 (0.002-0.003)	96.7 ± 4.0 (93.8-99.5)	0.14

Table S11. Inhibitory concentrations at 10% cytotoxicity based on nominal concentrations ($IC_{10,nom}$), nominal concentration that are corrected with plate binding ($IC_{10,nom,corr}$), freely dissolved cytotoxic concentrations ($IC_{10,free}$) with corresponding standard error (SE), and cell membrane concentrations at $IC_{10,free}$ ($IC_{10,membrane}$) in the AREc32 and AhR-CALUX assays.

Compounds	AREc32				AhR-CALUX			
	$IC_{10,nom}$ [µmol/L_w]	$IC_{10,nom,corr}$ [µmol/L_w]	$IC_{10,free}$ [µmol/L_w]	$IC_{10,membrane}$ [mmol/L_{lip}]	$IC_{10,nom}$ [µmol/L_w]	$IC_{10,nom,corr}$ [µmol/L_w]	$IC_{10,free}$ [µmol/L_w]	$IC_{10,membrane}$ [mmol/L_{lip}]
IM1-8 Cl	137 ± 30	137 ± 30	121 ± 29	902	2.5 ± 0.3	2.5 ± 0.3	2.4 ± 0.3	28.2
IM1-10 Cl	14.2 ± 6.1	14.2 ± 6.1	6.0 ± 0.9	184	0.38 ± 0.2	0.38 ± 0.2	0.21 ± 0.1	129
IM1-12 Cl	1.7 ± 0.2	1.7 ± 0.2	0.63 ± 0.1	169	0.050 ± 0.005	0.037 ± 0.005	0.0044 ± 0.0005	8.1
IM1-14 Cl	1.4 ± 0.2	1.2 ± 0.1	0.016 ± 0.002	48.5	0.060 ± 0.009	0.045 ± 0.007	$3.2 \times 10^{-4} \pm 4.7 \times 10^{-5}$	9.1
IM1-16 Cl	1.4 ± 0.6	1.2 ± 0.5	$2.6 \times 10^{-4} \pm 8.2 \times 10^{-5}$	15.7	0.083 ± 0.02	0.053 ± 0.01	$1.1 \times 10^{-5} \pm 2.1 \times 10^{-6}$	1.6
N11-10-1Ph Cl	5.9 ± 0.7	5.9 ± 0.7	2.8 ± 0.3	165	55.3 ± 7.5	55.3 ± 7.5	28.2 ± 3.9	N/A
N11-12-1Ph Cl	2.7 ± 0.5	2.7 ± 0.5	0.24 ± 0.04	1080	10.9 ± 2.8	10.9 ± 2.8	1.65 ± 0.4	N/A
N11-14-1Ph Cl	1.3 ± 0.1	1.3 ± 0.1	0.0033 ± 0.0003	42.0	5.7 ± 1.3	5.6 ± 1.3	0.015 ± 0.003	N/A
N11-16-1Ph Cl	1.3 ± 0.1	1.3 ± 0.1	$1.8 \times 10^{-5} \pm 3.7 \times 10^{-6}$	3.4	2.3 ± 0.6	2.1 ± 0.5	$4.5 \times 10^{-5} \pm 1.2 \times 10^{-5}$	N/A

N/A: data not available

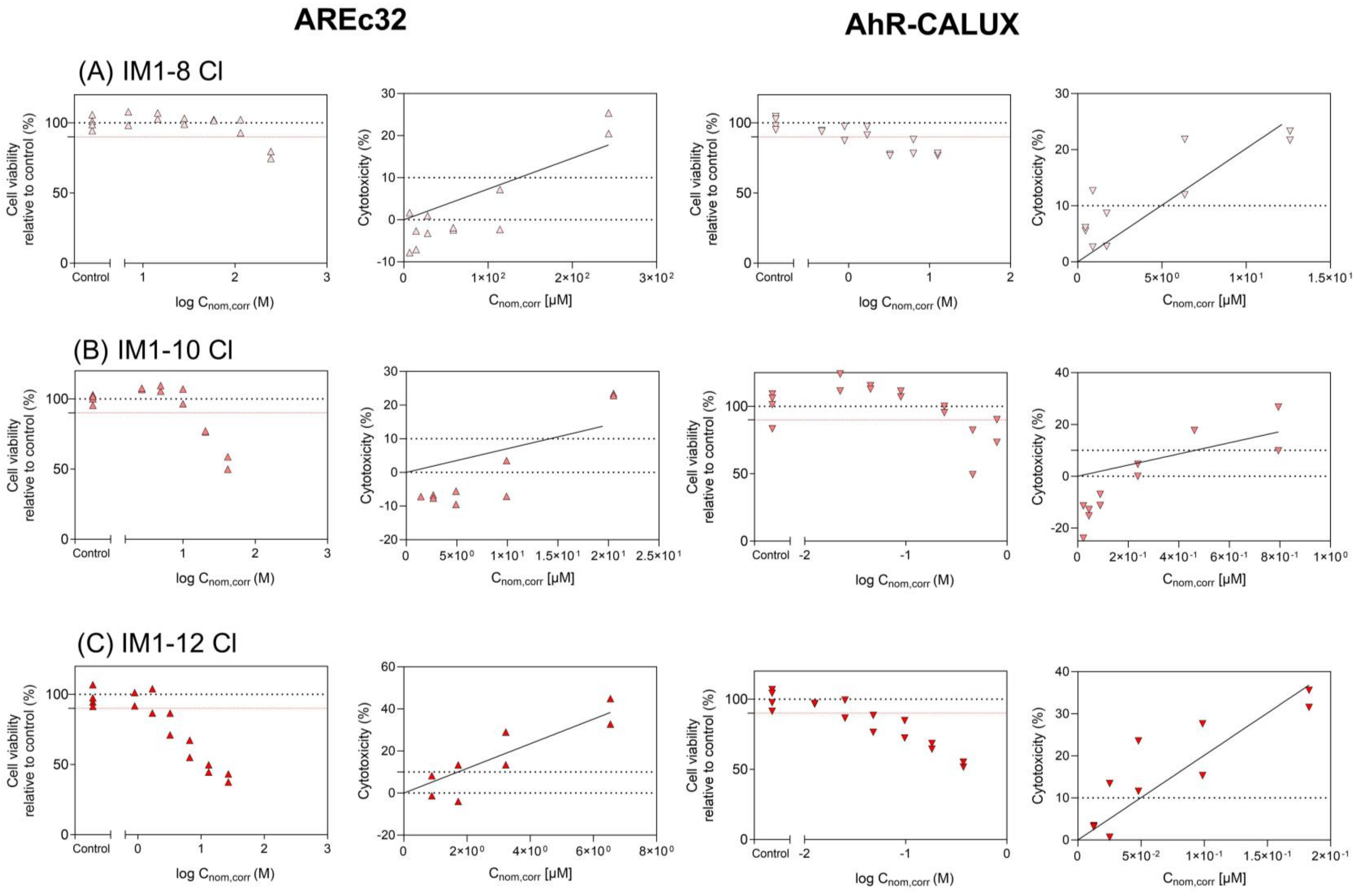
AREc32
AhR-CALUX
(A) IM1-8 Cl
(B) IM1-10 Cl
(C) IM1-12 Cl
Cell viability relative to control (%)
Cytotoxicity (%)
Control
log $C_{nom,corr}$ (M)
$C_{nom,corr}$ [µM]

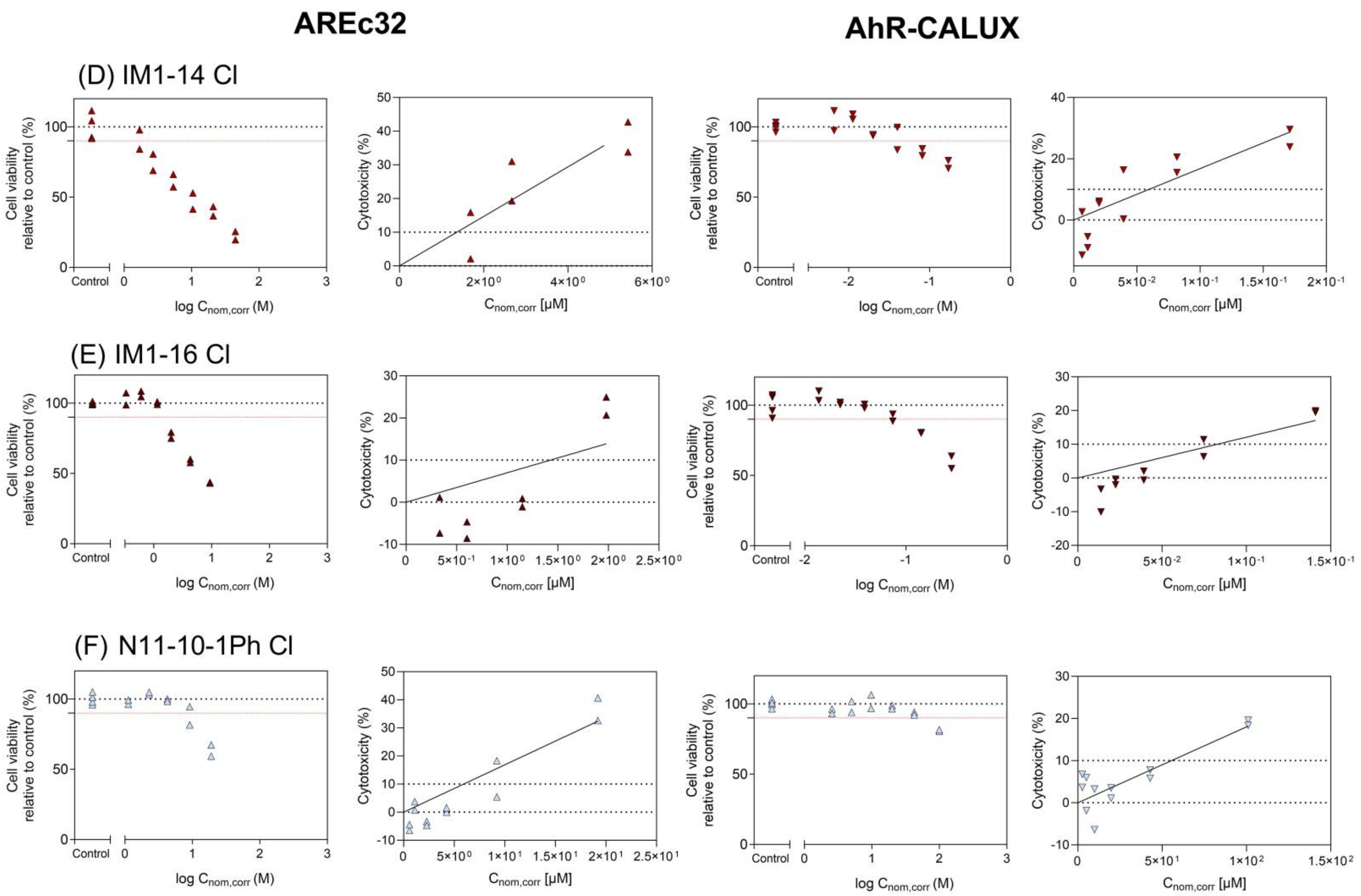

AREc32
AhR-CALUX
(D) IM1-14 Cl
(E) IM1-16 Cl
(F) N11-10-1Ph Cl
Cell viability relative to control (%)
Cytotoxicity (%)
log $C_{nom,corr}$ (M)
$C_{nom,corr}$ [µM]
Control

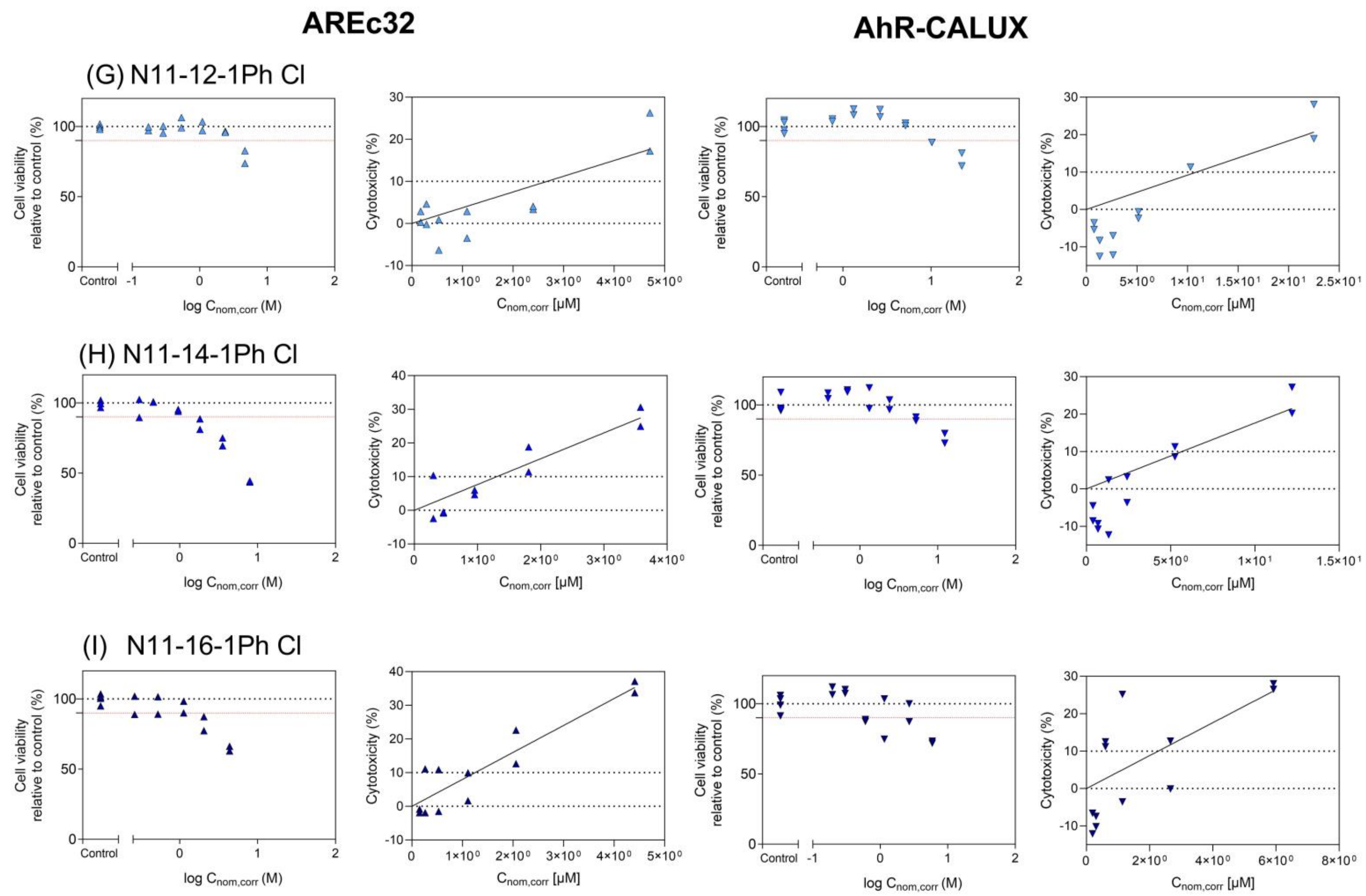

Figure S16. Concentration-response curves (based on nominal concentration corrected with plate binding ($C_{nom,corr}$) using eq 18 and linear range of concentration response curve of all test compounds in AREc32 (two figures on left side) and AhR-CALUX (two figures on right side).

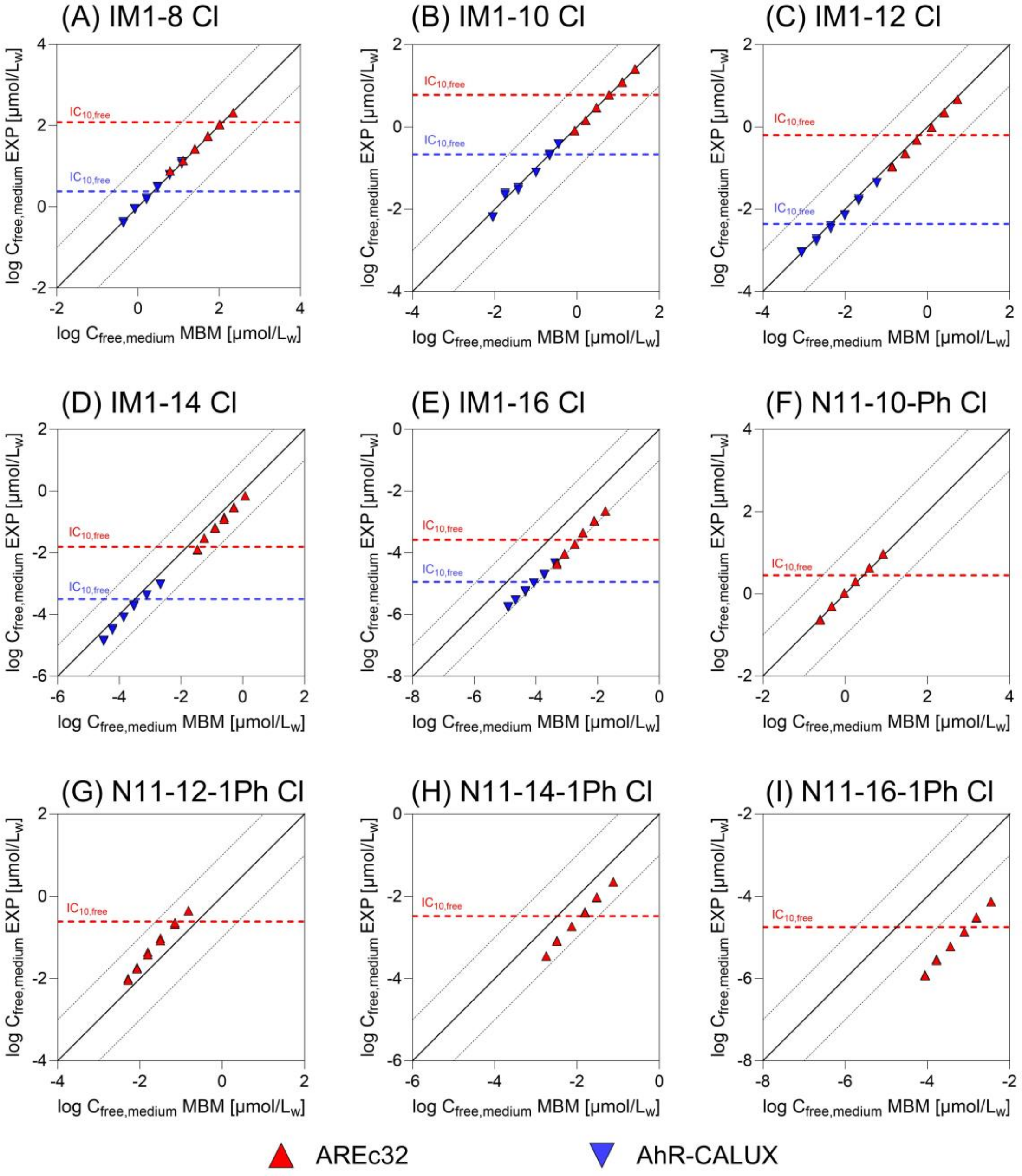

Figure S17. Freely dissolved concentration ($C_{free,medium}$, μmol/L_w) of IL cations in AREc32 (red triangles) and AhR-CALUX assay (blue inverted triangles) medium in the presence of cells after 24 h exposure. The $C_{free,medium}$ [μmol/L_w] values quantified using the experimental unbound fraction f_u (%) in bioassay medium (eq 15) were compared with $C_{free,medium}$ predicted by mass balance model (MBM, eq 19). The $C_{free,medium}$ of four N11-R-1Ph Cl was not predicted by MBM because the experimental cell-water partition coefficients ($K_{cell/w}$) were not available. If the experimental $C_{free,medium}$ agrees well with the prediction, the data points fall on the solid line. The black dotted lines indicate one log unit deviation from ideal agreement. Freely dissolved concentration causing 10% cytotoxicity ($IC_{10,free}$) are remarked with dashed line in matching color.

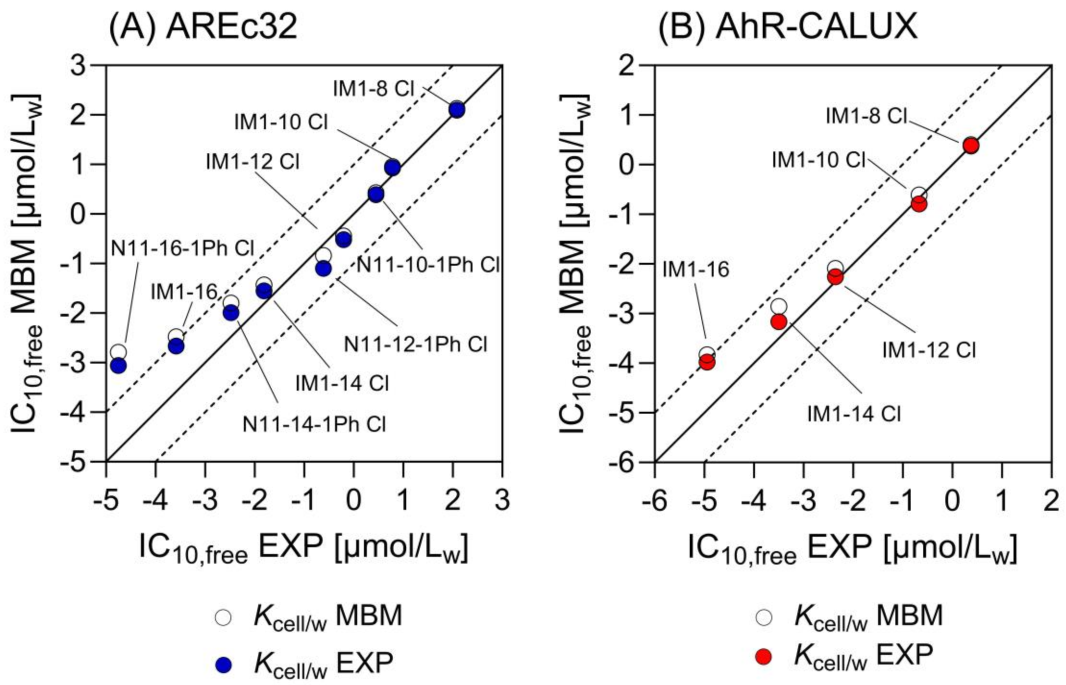

Figure S18. Comparison of the quantified freely dissolved concentration causing 10% cytotoxicity ($IC_{10,free}$) with $IC_{10,free}$ predicted by using $K_{cell/w}$ modeled (eq 20, empty circles) or experimentally quantified (filled circles) in (A) AREc32 assay and (B) AhR-CALUX assay. If the experimental $IC_{10,free}$ are equal to the modeled $IC_{10,free}$, the data points fall on the solid line. The black dotted lines indicate one log unit deviation from agreement.

Reference

(1) Qin, W.; Henneberger, L.; Glüge, J.; König, M.; Escher, B. I. Baseline Toxicity Model to Identify the Specific and Nonspecific Effects of Per- and Polyfluoroalkyl Substances in Cell-Based Bioassays. *Environ. Sci. Technol.* **2023**. https://doi.org/10.1021/acs.est.3c09950.

(2) Kowalska, D.; Stolte, S.; Wyrzykowski, D.; Stepnowski, P.; Dołżonek, J. Interaction of Ionic Liquids with Human Serum Albumin in the View of Bioconcentration: A Preliminary Study. *Chem. Pap.* **2022**, *76* (4), 2405–2417. https://doi.org/10.1007/s11696-021-02021-y.

(3) Satish, L.; Millan, S.; Sahoo, H. Spectroscopic Insight into the Interaction of Bovine Serum Albumin with Imidazolium-Based Ionic Liquids in Aqueous Solution. *Luminescence* **2017**, *32* (5), 695–705. https://doi.org/10.1002/bio.3239.

(4) Huang, R.; Zhang, S.; Pan, L.; Li, J.; Liu, F.; Liu, H. Spectroscopic Studies on the Interactions between Imidazolium Chloride Ionic Liquids and Bovine Serum Albumin. *Spectrochim. Acta - Part A Mol. Biomol. Spectrosc.* **2013**, *104*, 377–382. https://doi.org/10.1016/j.saa.2012.11.087.

(5) Shu, Y.; Liu, M.; Chen, S.; Chen, X.; Wang, J. New Insight into Molecular Interactions of Imidazolium Ionic Liquids with Bovine Serum Albumin. *J. Phys. Chem. B* **2011**, *115* (42), 12306–12314. https://doi.org/10.1021/jp2071925.

(6) Timmer, N.; Droge, S. T. J. Sorption of Cationic Surfactants to Artificial Cell Membranes: Comparing Phospholipid Bilayers with Monolayer Coatings and Molecular Simulations. *Environ. Sci. Technol.* **2017**, *51* (5), 2890–2898. https://doi.org/10.1021/acs.est.6b05662.

(7) Zhou, T.; Ao, M.; Xu, G.; Liu, T.; Zhang, J. Interactions of Bovine Serum Albumin with Cationic Imidazolium and Quaternary Ammonium Gemini Surfactants: Effects of Surfactant Architecture. *J. Colloid Interface Sci.* **2013**, *389* (1), 175–181. https://doi.org/10.1016/j.jcis.2012.08.067.

(8) Dołżonek, J.; Cho, C. W.; Stepnowski, P.; Markiewicz, M.; Thöming, J.; Stolte, S. Membrane Partitioning of Ionic Liquid Cations, Anions and Ion Pairs – Estimating the Bioconcentration Potential of Organic Ions. *Environ. Pollut.* **2017**, *228*, 378–389. https://doi.org/10.1016/j.envpol.2017.04.079.

(9) Cho, C. W.; Stolte, S.; Yun, Y. S. Validation and Updating of QSAR Models for Partitioning Coefficients of Ionic Liquids in Octanol-Water and Development of a New LFER Model. *Sci. Total Environ.* **2018**, *633*, 920–928. https://doi.org/10.1016/j.scitotenv.2018.03.225.

(10) Ranke, J.; Müller, A.; Bottin-Weber, U.; Stock, F.; Stolte, S.; Arning, J.; Störmann, R.; Jastorff, B. Lipophilicity Parameters for Ionic Liquid Cations and Their Correlation to in Vitro Cytotoxicity. *Ecotoxicol. Environ. Saf.* **2007**, *67* (3), 430–438. https://doi.org/10.1016/j.ecoenv.2006.08.008.

(11) Cho, C. W.; Preiss, U.; Jungnickel, C.; Stolte, S.; Arning, J.; Ranke, J.; Klamt, A.; Krossing, I.; Thöming, J. Ionic Liquids: Predictions of Physicochemical Properties with Experimental and/or DFT-Calculated LFER Parameters to Understand Molecular Interactions in Solution. *J. Phys. Chem. B* **2011**, *115* (19), 6040–6050. https://doi.org/10.1021/jp200042f.

(12) Abraham, M. H.; Acree, W. E. Equations for the Transfer of Neutral Molecules and Ionic Species from Water to Organic Phases. *J. Org. Chem.* **2010**, *75* (4), 1006–1015. https://doi.org/10.1021/jo902388n.

(13) Bittermann, K.; Spycher, S.; Goss, K. U. Comparison of Different Models Predicting the Phospholipid-Membrane Water Partition Coefficients of Charged Compounds. *Chemosphere* **2016**, *144*, 382–391. https://doi.org/10.1016/j.chemosphere.2015.08.065.

(14) Stolte, S.; Arning, J.; Bottin-Weber, U.; Matzke, M.; Stock, F.; Thiele, K.; Uerdingen, M.; Welz-Biermann, U.; Jastorff, B.; Ranke, J. Anion Effects on the Cytotoxicity of Ionic Liquids. *Green Chem.* **2006**, *8* (7), 621–629. https://doi.org/10.1039/b602161a.

(15) Stolte, S.; Matzke, M.; Arning, J.; Böschen, A.; Pitner, W. R.; Welz-Biermann, U.; Jastorff, B.;

Ranke, J. Effects of Different Head Groups and Functionalised Side Chains on the Cytotoxicity of Ionic Liquids. *Green Chem.* **2007**, *9* (11), 1170–1179. https://doi.org/10.1039/b711119c.

(16) Klamt, A.; Huniar, U.; Spycher, S.; Keldenich, J. COSMOmic: A Mechanistic Approach to the Calculation of Membrane-Water Partition Coefficients and Internal Distributions within Membranes and Micelles. *J. Phys. Chem. B* **2008**, *112* (38), 12148–12157. https://doi.org/10.1021/jp801736k.

(17) Bae, E.; Beil, S.; König, M.; Stolte, S.; Escher, B. I.; Markiewicz, M. The Mode of Toxic Action of Ionic Liquids: Narrowing down Possibilities Using High-Throughput, in Vitro Cell-Based Bioassays. *Environ. Int.* **2024**. https://doi.org/doi.org/10.1016/j.envint.2024.109089.

(18) Cho, C. W.; Park, J. S.; Stolte, S.; Yun, Y. S. Modelling for Antimicrobial Activities of Ionic Liquids towards Escherichia Coli, Staphylococcus Aureus and Candida Albicans Using Linear Free Energy Relationship Descriptors. *J. Hazard. Mater.* **2016**, *311*, 168–175. https://doi.org/10.1016/j.jhazmat.2016.03.006.

(19) Cho, C. W.; Ranke, J.; Arning, J.; Thöming, J.; Preiss, U.; Jungnickel, C.; Diedenhofen, M.; Krossing, I.; Stolte, S. In Silico Modelling for Predicting the Cationic Hydrophobicity and Cytotoxicity of Ionic Liquids towards the Leukemia Rat Cell Line, Vibrio Fischeri and Scenedesmus Vacuolatus Based on Molecular Interaction Potentials of Ions. *SAR QSAR Environ. Res.* **2013**, *24* (10), 863–882. https://doi.org/10.1080/1062936X.2013.821092.

(20) Lee, S. H.; Lee, S. B. Octanol/Water Partition Coefficients of Ionic Liquids. *J. Chem. Technol. Biotechnol.* **2009**, *84* (2), 202–207. https://doi.org/10.1002/jctb.2025.

(21) Hodges, G.; Eadsforth, C.; Bossuyt, B.; Bouvy, A.; Enrici, M. H.; Geurts, M.; Kotthoff, M.; Michie, E.; Miller, D.; Müller, J.; Oetter, G.; Roberts, J.; Schowanek, D.; Sun, P.; Venzmer, J. A Comparison of Log K Ow (n-Octanol–Water Partition Coefficient) Values for Non-Ionic, Anionic, Cationic and Amphoteric Surfactants Determined Using Predictions and Experimental Methods. *Environ. Sci. Eur.* **2019**, *31* (1), 1–18. https://doi.org/10.1186/s12302-018-0176-7.

(22) Stolte, S.; Matzke, M.; Arning, J.; Böschen, A.; Pitner, W. R.; Welz-Biermann, U.; Jastorff, B.; Ranke, J. Effects of Different Head Groups and Functionalised Side Chains on the Aquatic Toxicity of Ionic Liquids. *Green Chem.* **2007**, *9* (11), 1170–1179. https://doi.org/10.1039/b711119c.

(23) Endo, S.; Escher, B. I.; Goss, K. U. Capacities of Membrane Lipids to Accumulate Neutral Organic Chemicals. *Environ. Sci. Technol.* **2011**, *45* (14), 5912–5921. https://doi.org/10.1021/es200855w.

(24) Escher, B. I.; Schwarzenbach, R. P. Partitioning of Substituted Phenols in Liposome−Water, Biomembrane−Water, and Octanol−Water Systems. *Environ. Sci. Technol.* **1996**, *30* (1), 260–270. https://doi.org/10.1021/es9503084.

(25) Armitage, J. M.; Arnot, J. A.; Wania, F.; Mackay, D. Development and Evaluation of a Mechanistic Bioconcentration Model for Ionogenic Organic Chemicals in Fish. *Environ. Toxicol. Chem.* **2013**, *32* (1), 115–128. https://doi.org/10.1002/etc.2020.

(26) Müller, M. T.; Zehnder, A. J. B.; Escher, B. I. Liposome—Water and Octanol—Water Partitioning of Alcohol Ethoxylates. *Environ. Toxicol. Chem.* **1999**, *18* (10), 2191–2198. https://doi.org/10.1002/etc.5620181011.

(27) Cho, C.-W.; Stolte, S.; Yun, Y.-S.; Krossing, I.; Thöming, J. In Silico Prediction of Linear Free Energy Relationship Descriptors of Neutral and Ionic Compounds. *RSC Adv.* **2015**, *5* (98), 80634–80642. https://doi.org/10.1039/C5RA13595H.
